# Supplementary figures and images for: aCPSF1 cooperates with terminator U-tract to dictate archaeal transcription termination efficacy (part 1 of 2)
Source: eLife. 2021 Dec 29;10:e70464. doi: 10.7554/eLife.70464 (PMC8716108; doi:10.7554/eLife.70464)

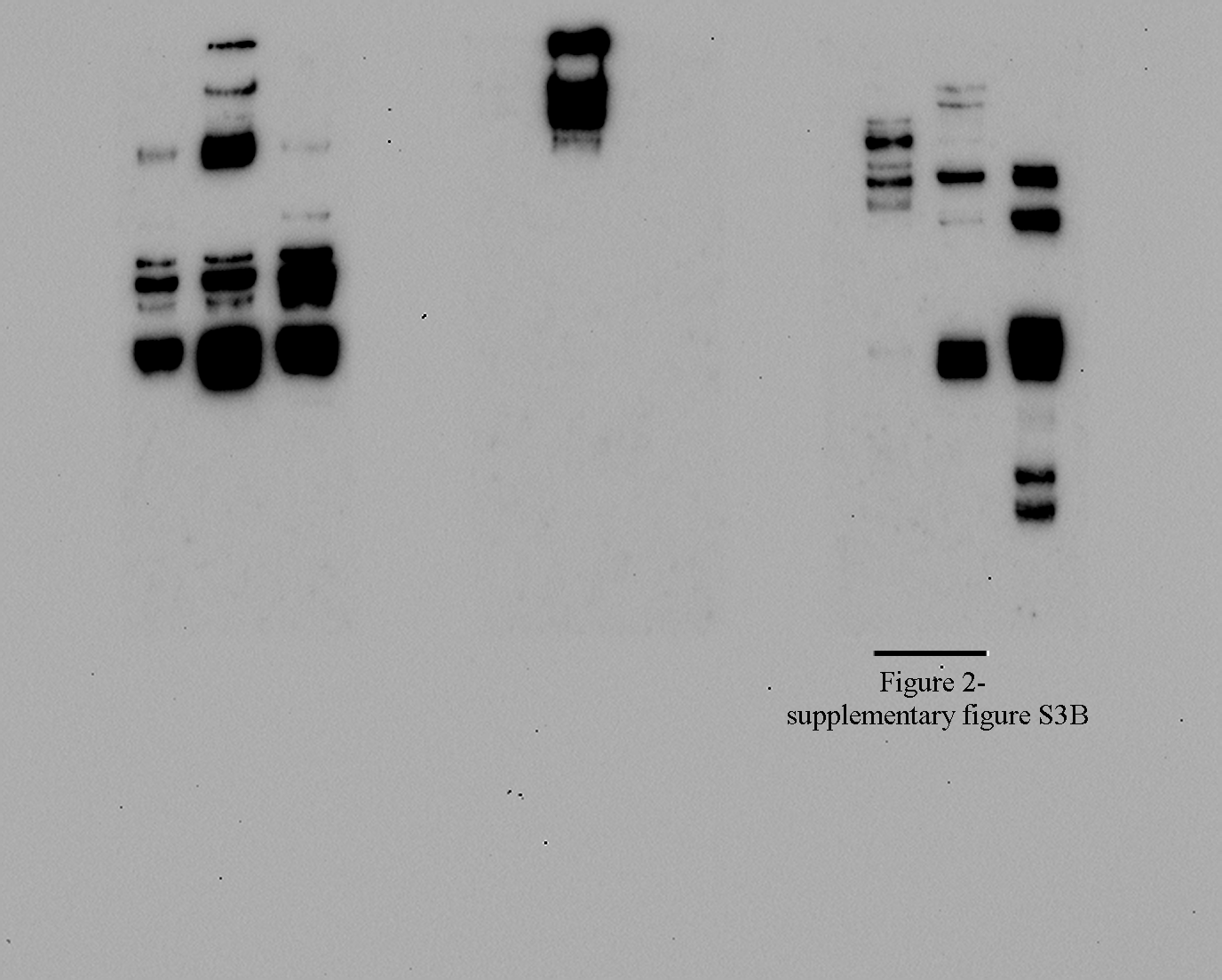

Supplement: Figure 2—figure supplement 3—source data 1. [file elife-70464-fig2-figsupp3-data1.zip › Figure 2-figure supplement 3-source data 1/Figure 2-figure supplement 3B-Labeled.tif]

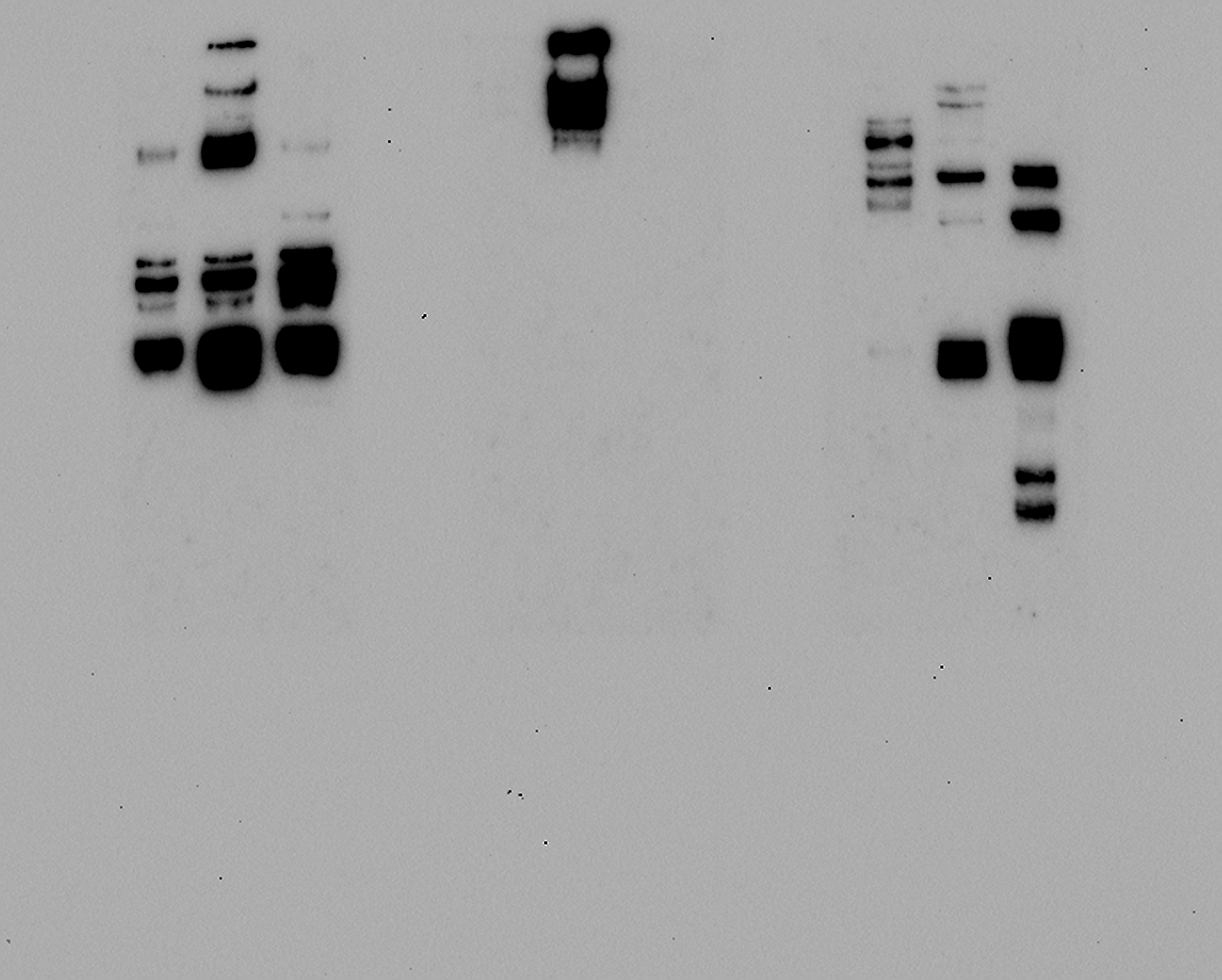

Supplement: Figure 2—figure supplement 3—source data 1. [file elife-70464-fig2-figsupp3-data1.zip › Figure 2-figure supplement 3-source data 1/Figure 2-figure supplement 3B-Original.tif]

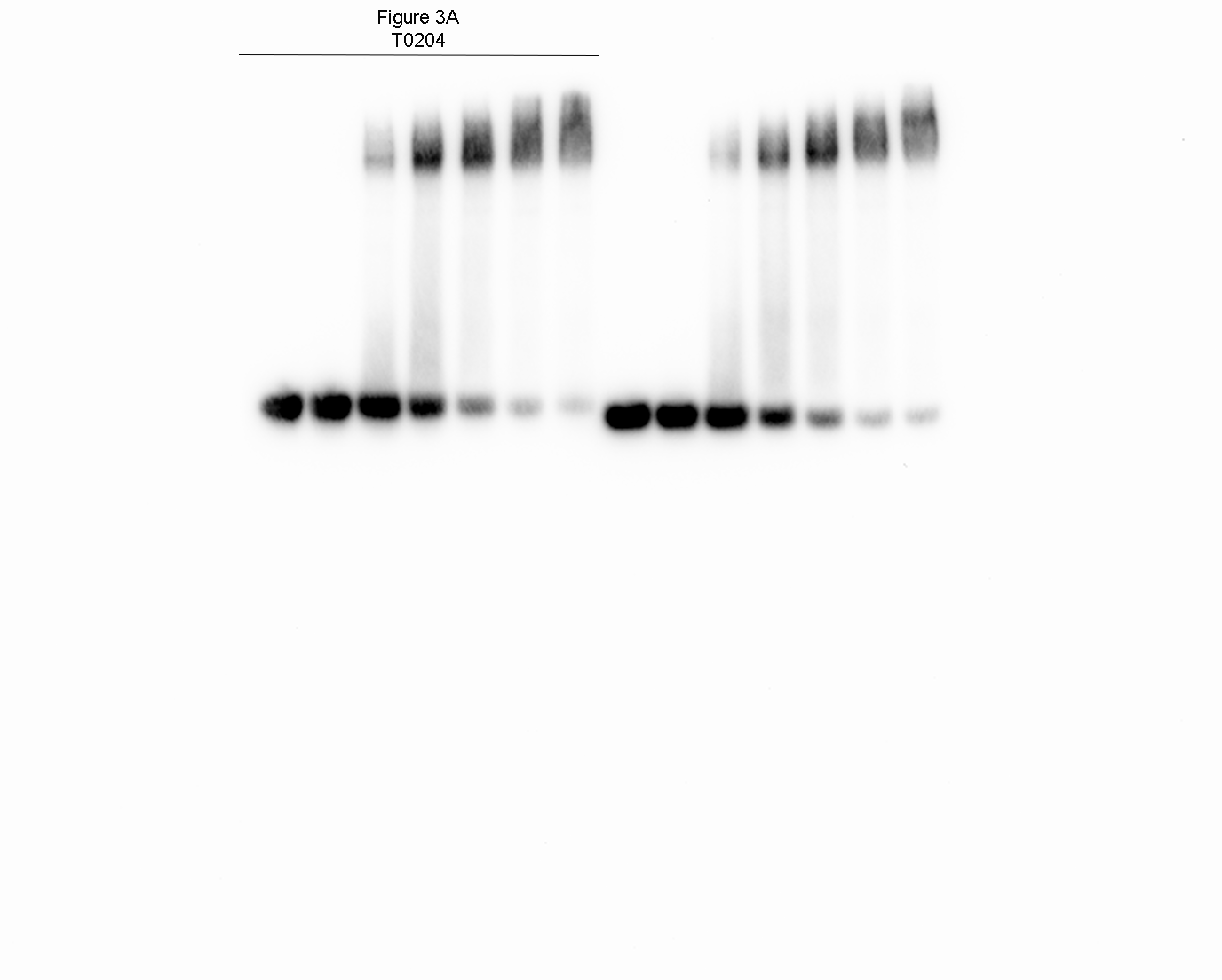

Supplement: Figure 3—source data 1. [file elife-70464-fig3-data1.zip › Figure 3-source data 1/Figure 3A-T0204-Labeled.tif]

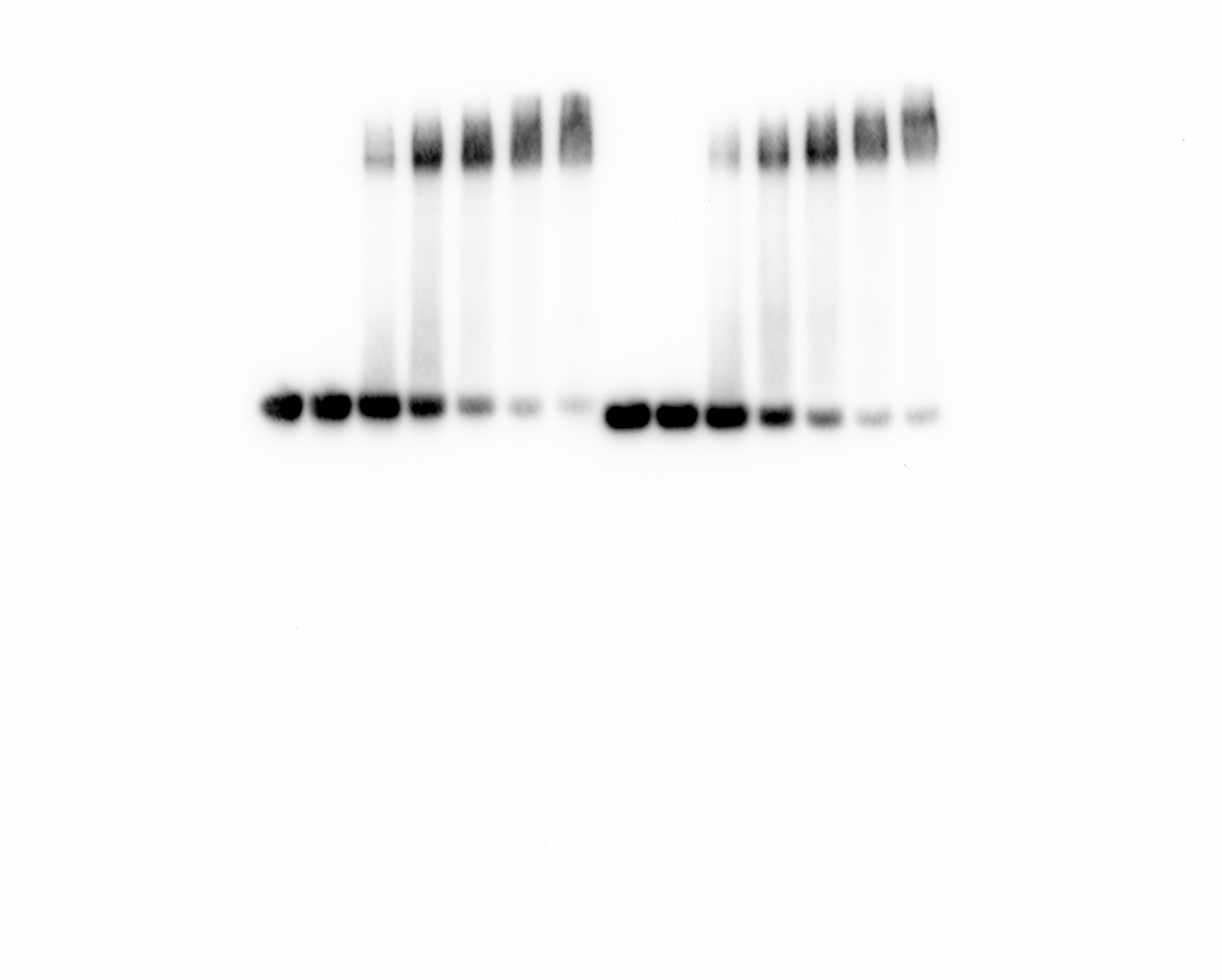

Supplement: Figure 3—source data 1. [file elife-70464-fig3-data1.zip › Figure 3-source data 1/Figure 3A-T0204-Original·.tif]

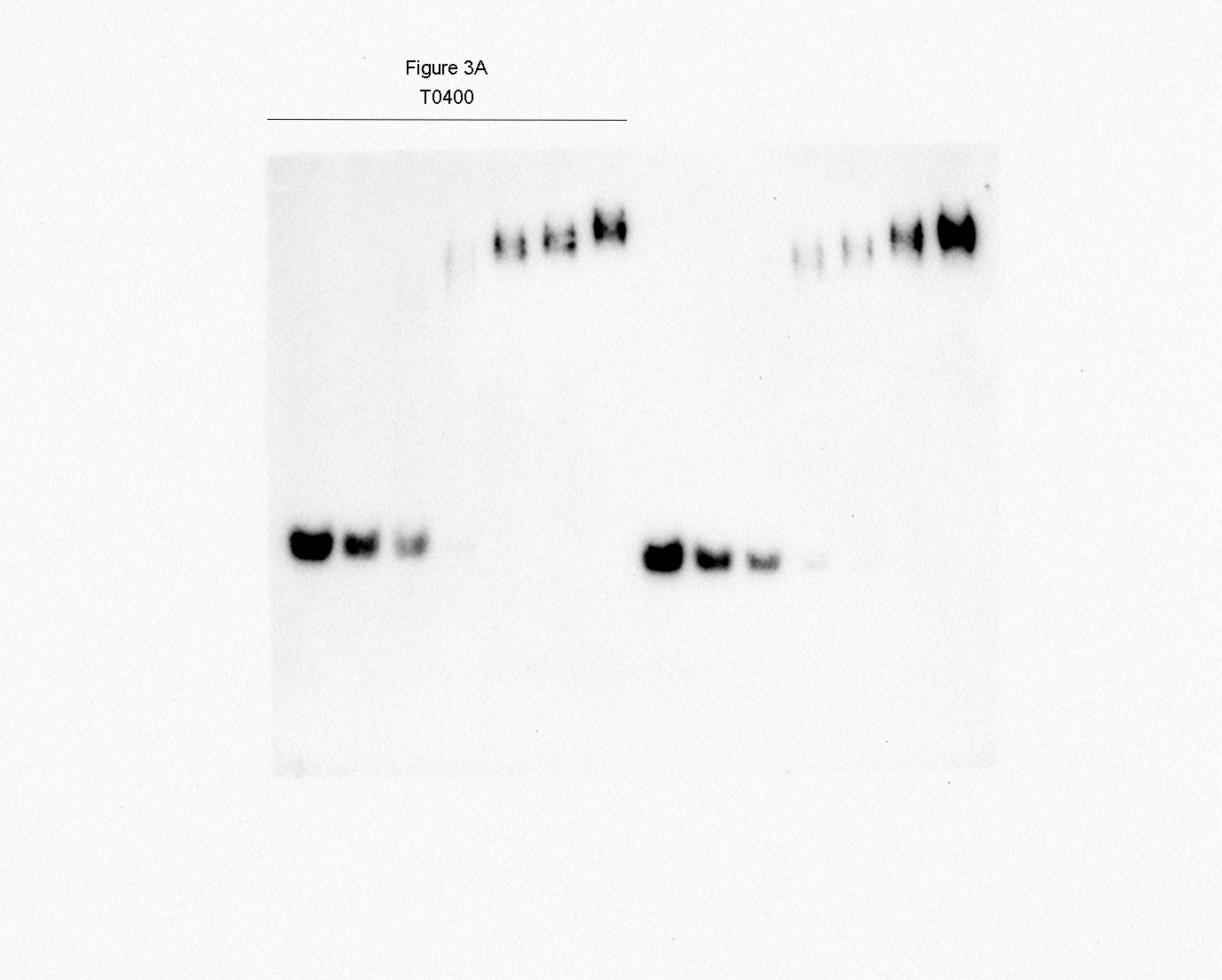

Supplement: Figure 3—source data 1. [file elife-70464-fig3-data1.zip › Figure 3-source data 1/Figure 3A-T0400-Labeled.tif]

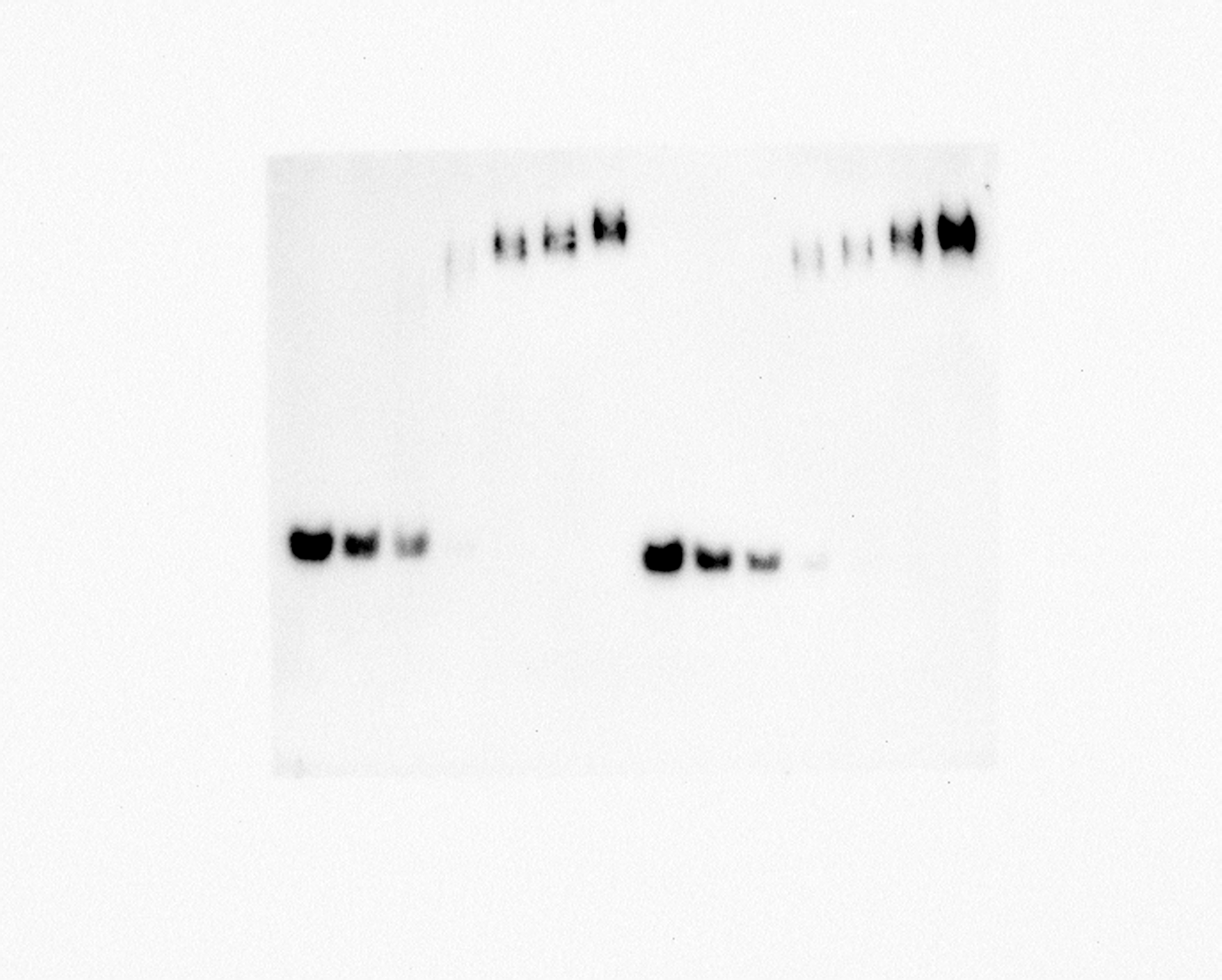

Supplement: Figure 3—source data 1. [file elife-70464-fig3-data1.zip › Figure 3-source data 1/Figure 3A-T0400-Original.tif]

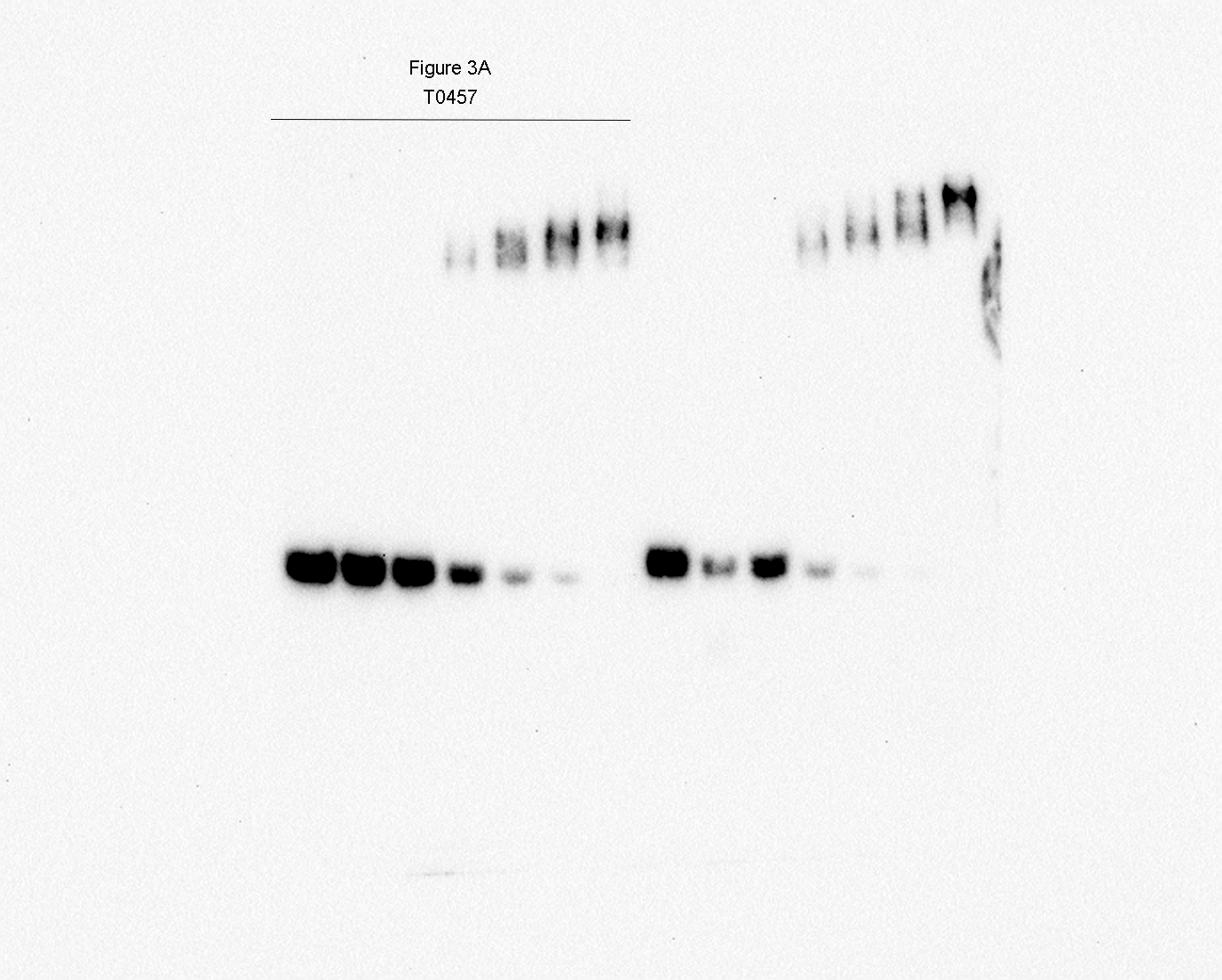

Supplement: Figure 3—source data 1. [file elife-70464-fig3-data1.zip › Figure 3-source data 1/Figure 3A-T0457-Labeled.tif]

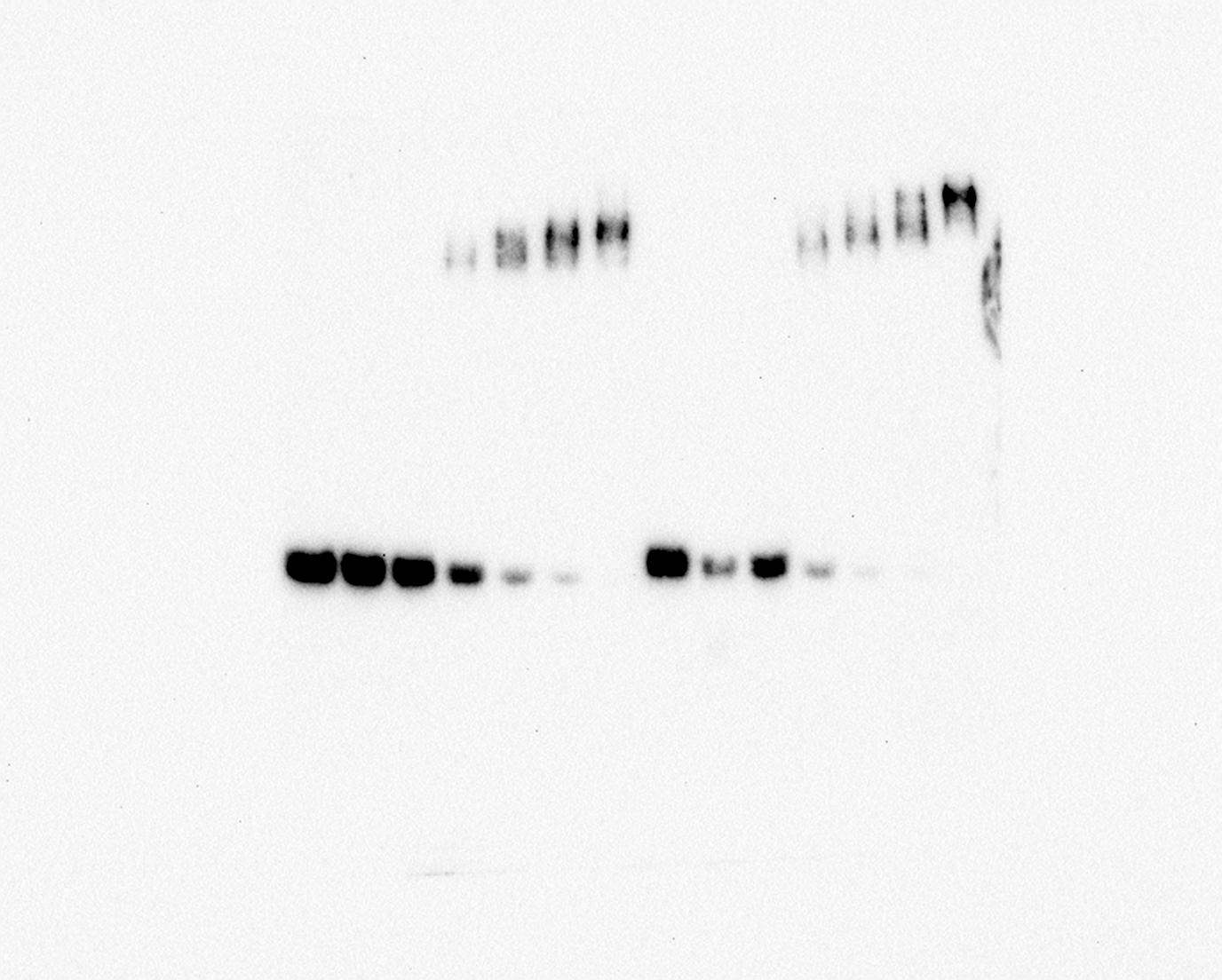

Supplement: Figure 3—source data 1. [file elife-70464-fig3-data1.zip › Figure 3-source data 1/Figure 3A-T0457-Original.tif]

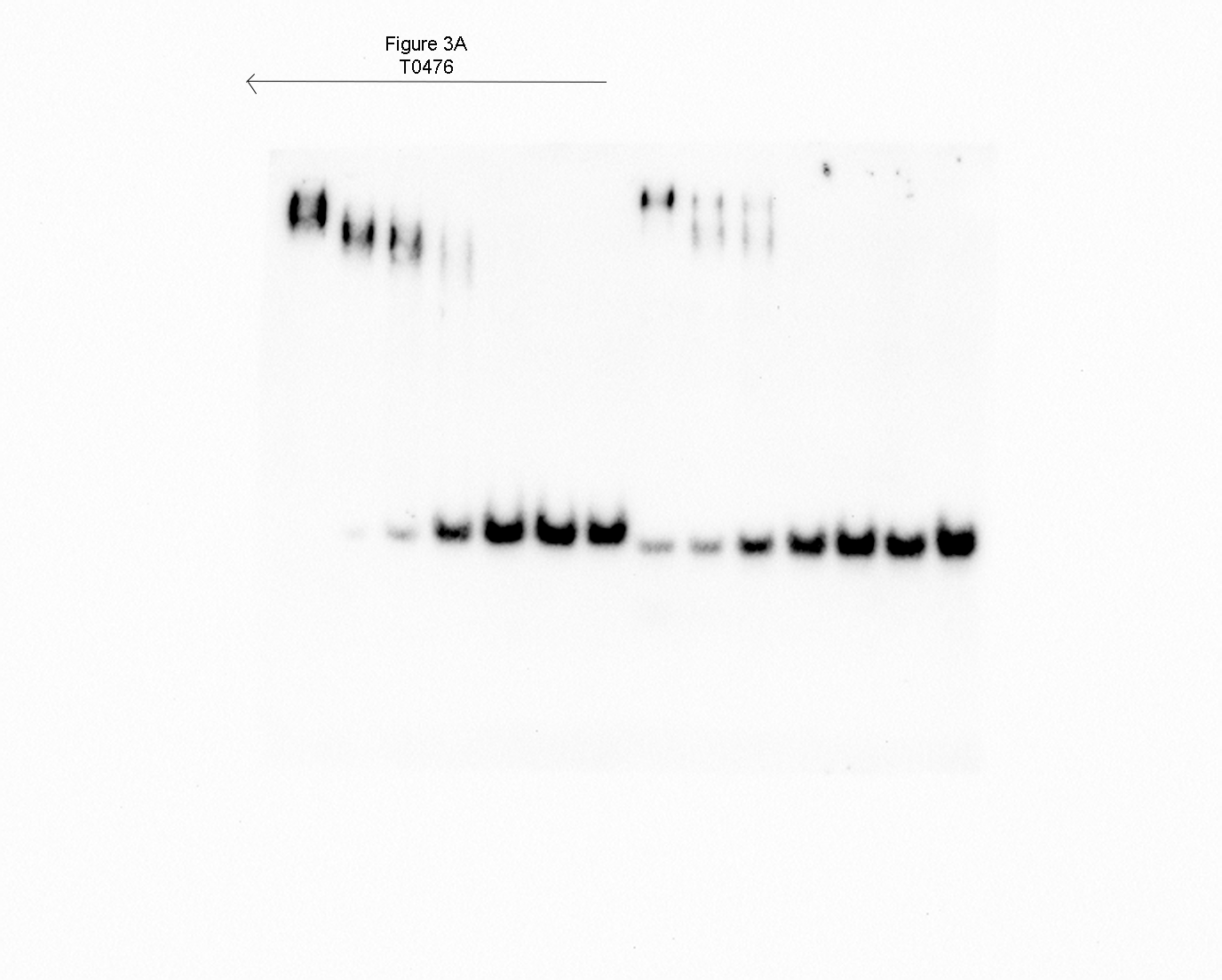

Supplement: Figure 3—source data 1. [file elife-70464-fig3-data1.zip › Figure 3-source data 1/Figure 3A-T0476-Labeled.tif]

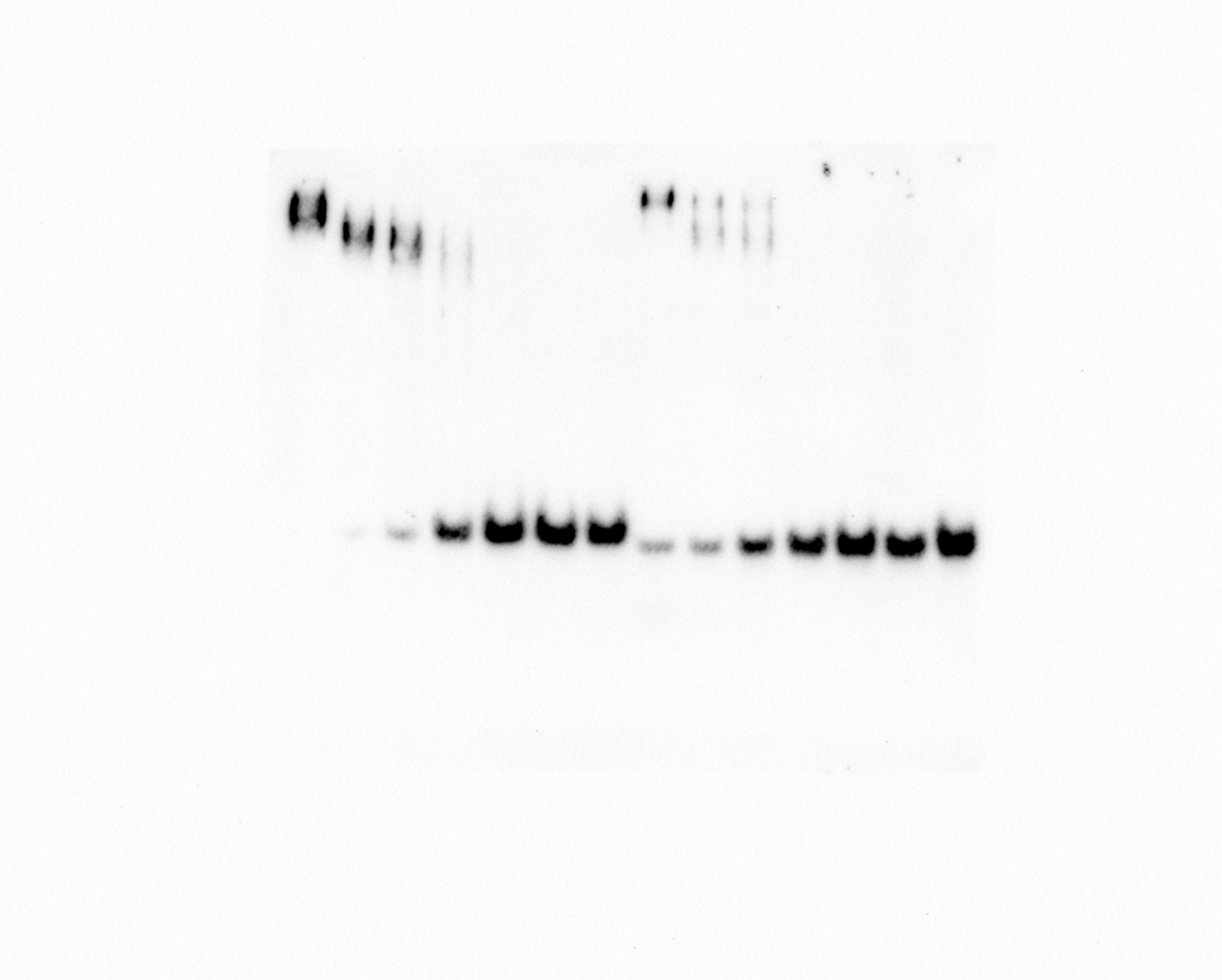

Supplement: Figure 3—source data 1. [file elife-70464-fig3-data1.zip › Figure 3-source data 1/Figure 3A-T0476-Original.tif]

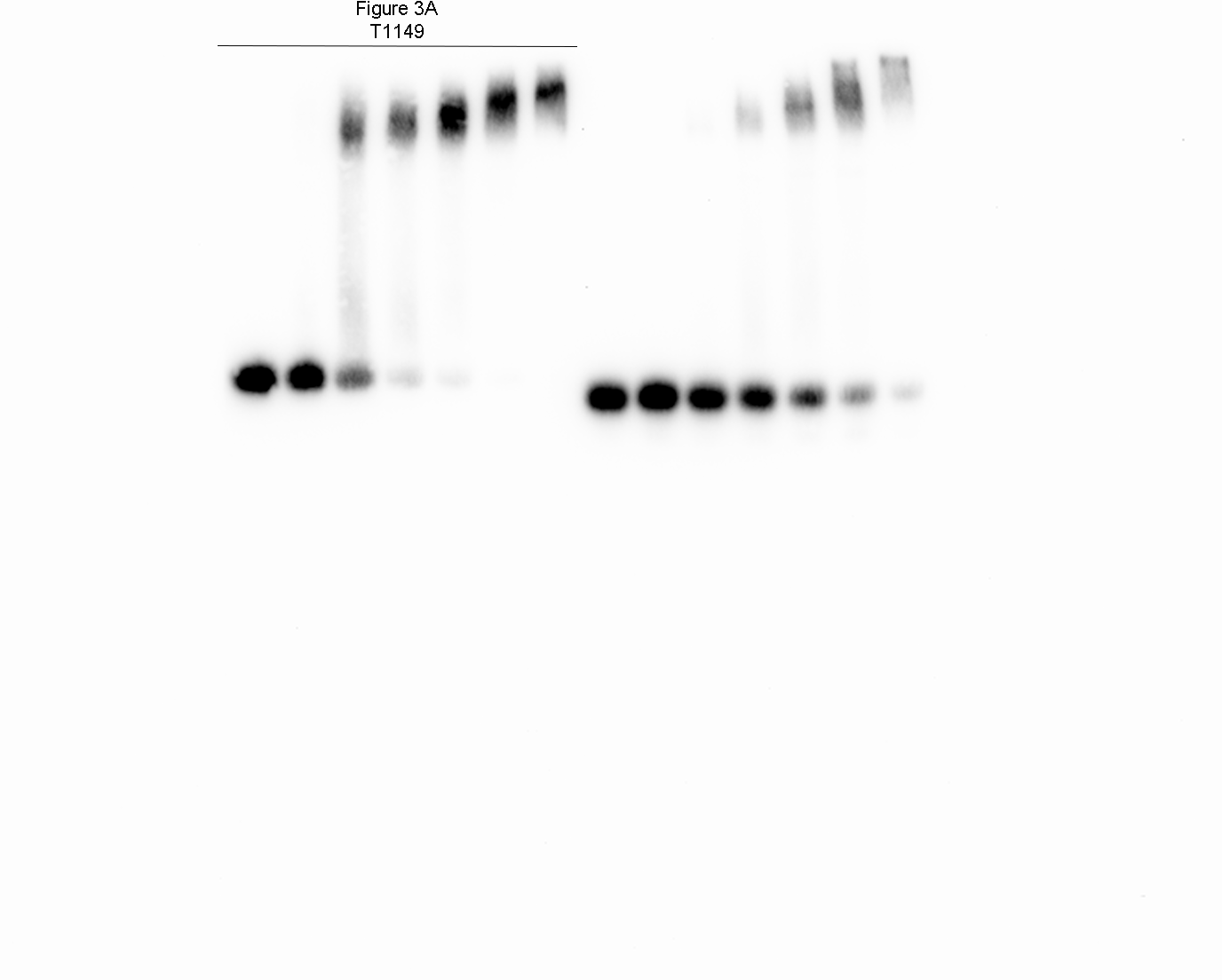

Supplement: Figure 3—source data 1. [file elife-70464-fig3-data1.zip › Figure 3-source data 1/Figure 3A-T1149-Labeled.tif]

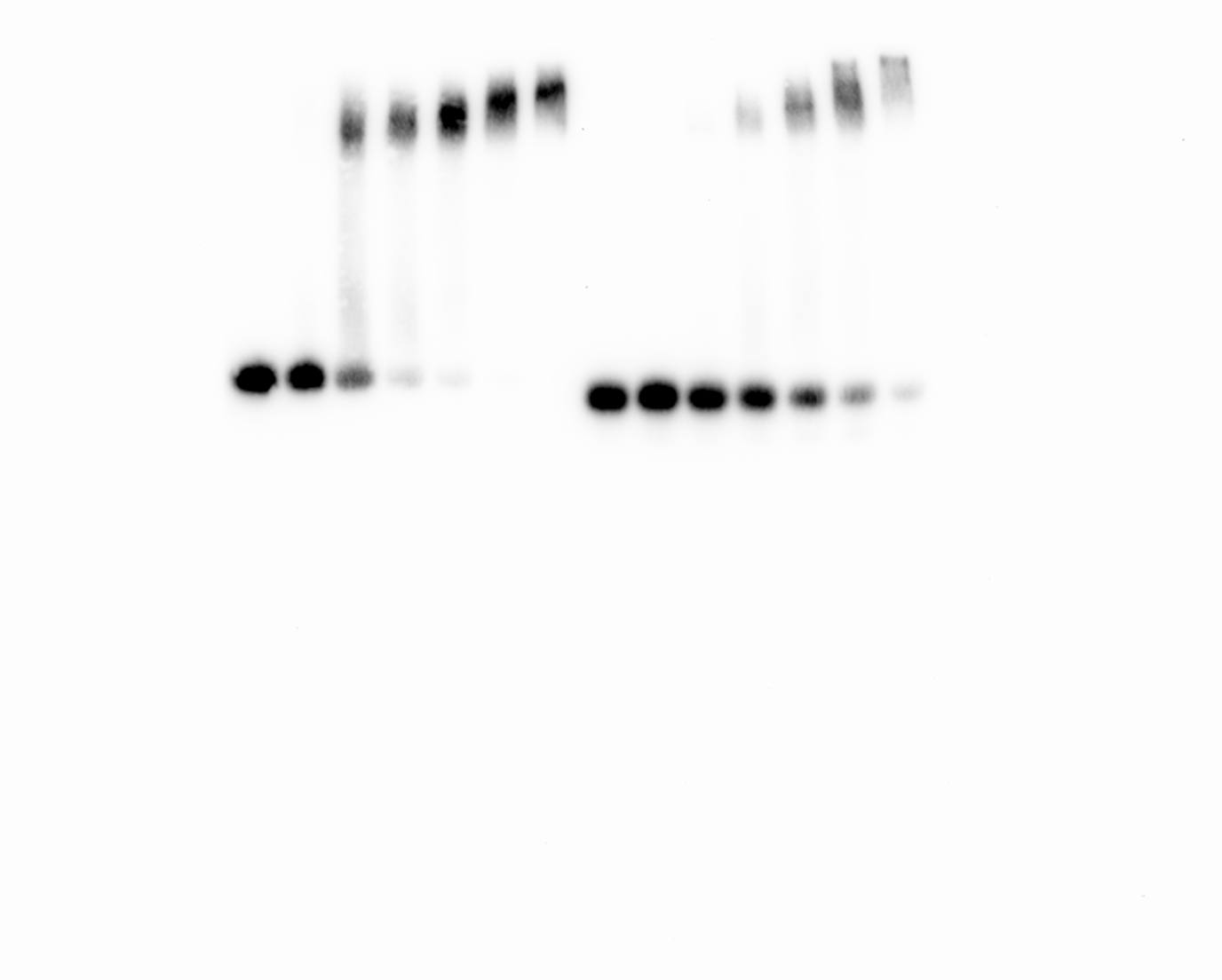

Supplement: Figure 3—source data 1. [file elife-70464-fig3-data1.zip › Figure 3-source data 1/Figure 3A-T1149-Original.tif]

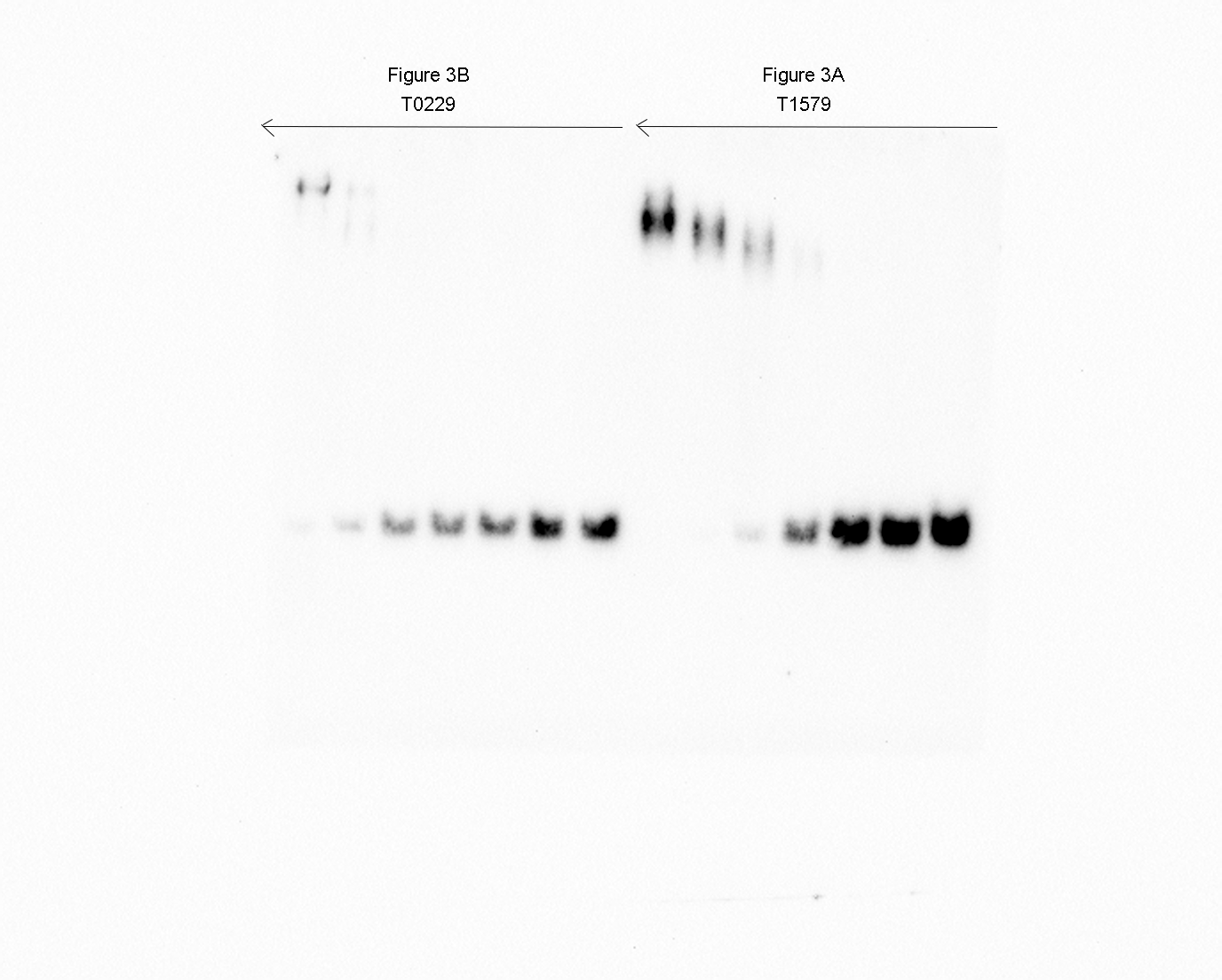

Supplement: Figure 3—source data 1. [file elife-70464-fig3-data1.zip › Figure 3-source data 1/Figure 3A-T1579 Figure 3B-T0229-Labeled.tif]

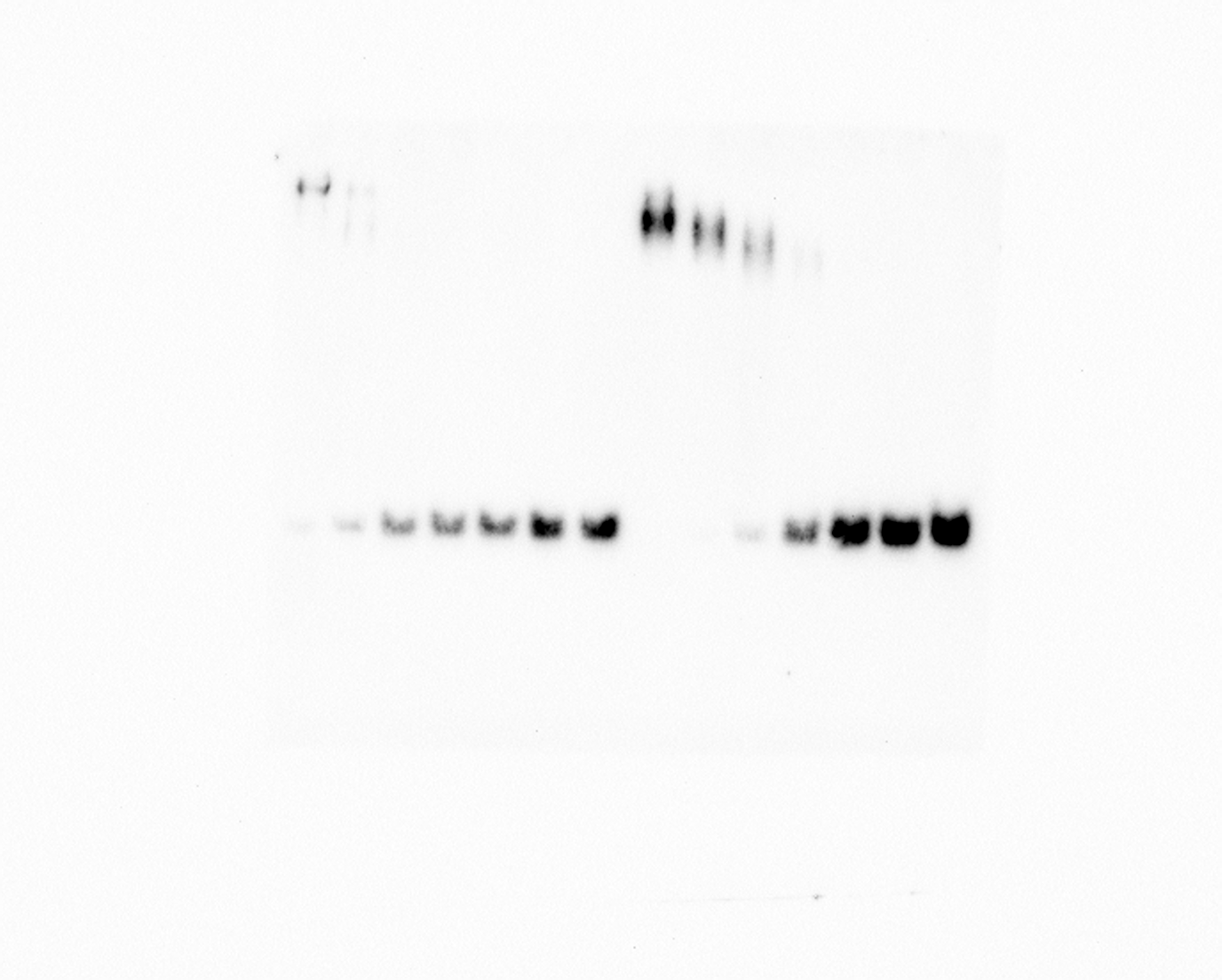

Supplement: Figure 3—source data 1. [file elife-70464-fig3-data1.zip › Figure 3-source data 1/Figure 3A-T1579 Figure 3B-T0229-Original.tif]

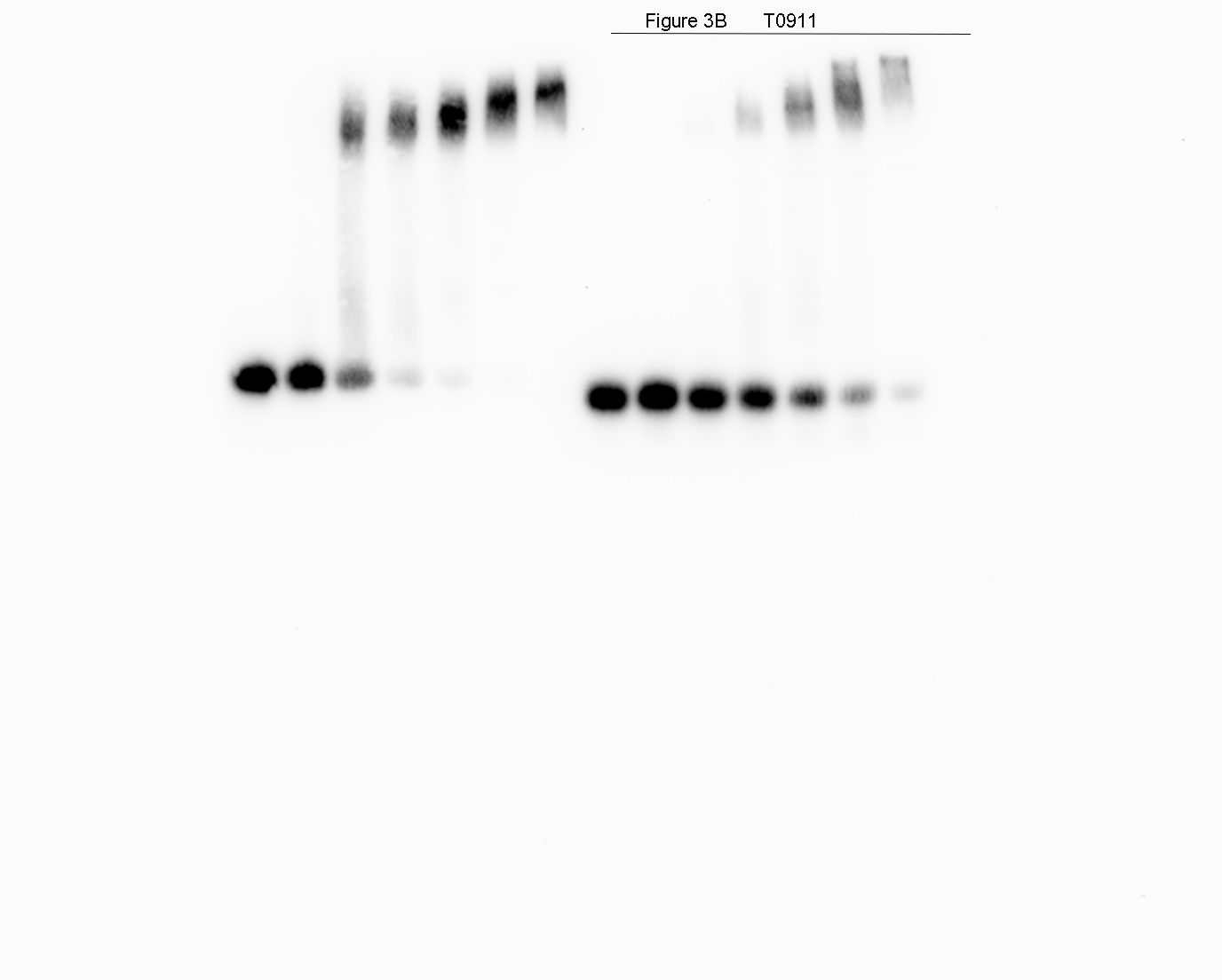

Supplement: Figure 3—source data 1. [file elife-70464-fig3-data1.zip › Figure 3-source data 1/Figure 3B-T0911-Labeled.tif]

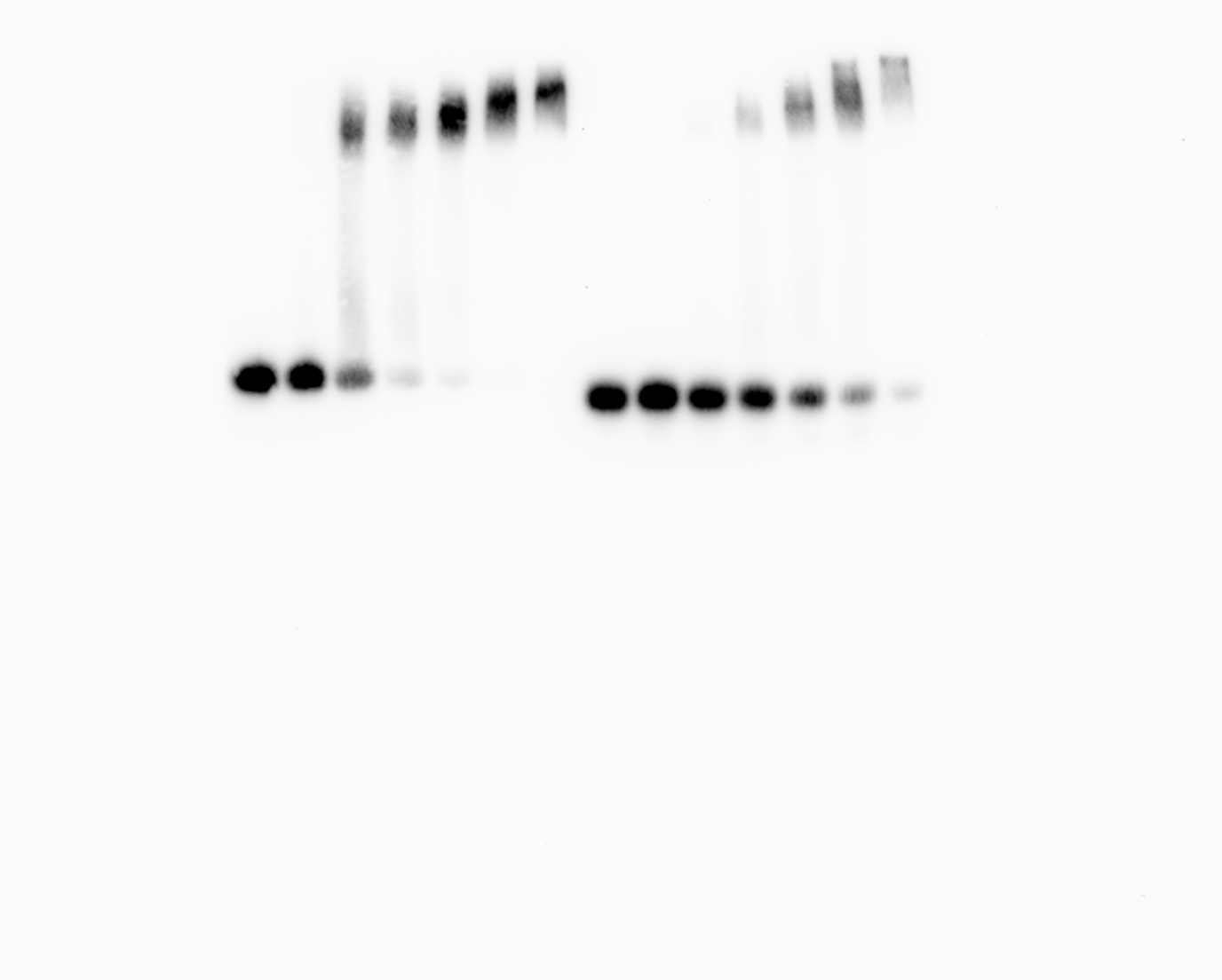

Supplement: Figure 3—source data 1. [file elife-70464-fig3-data1.zip › Figure 3-source data 1/Figure 3B-T0911-Original.tif]

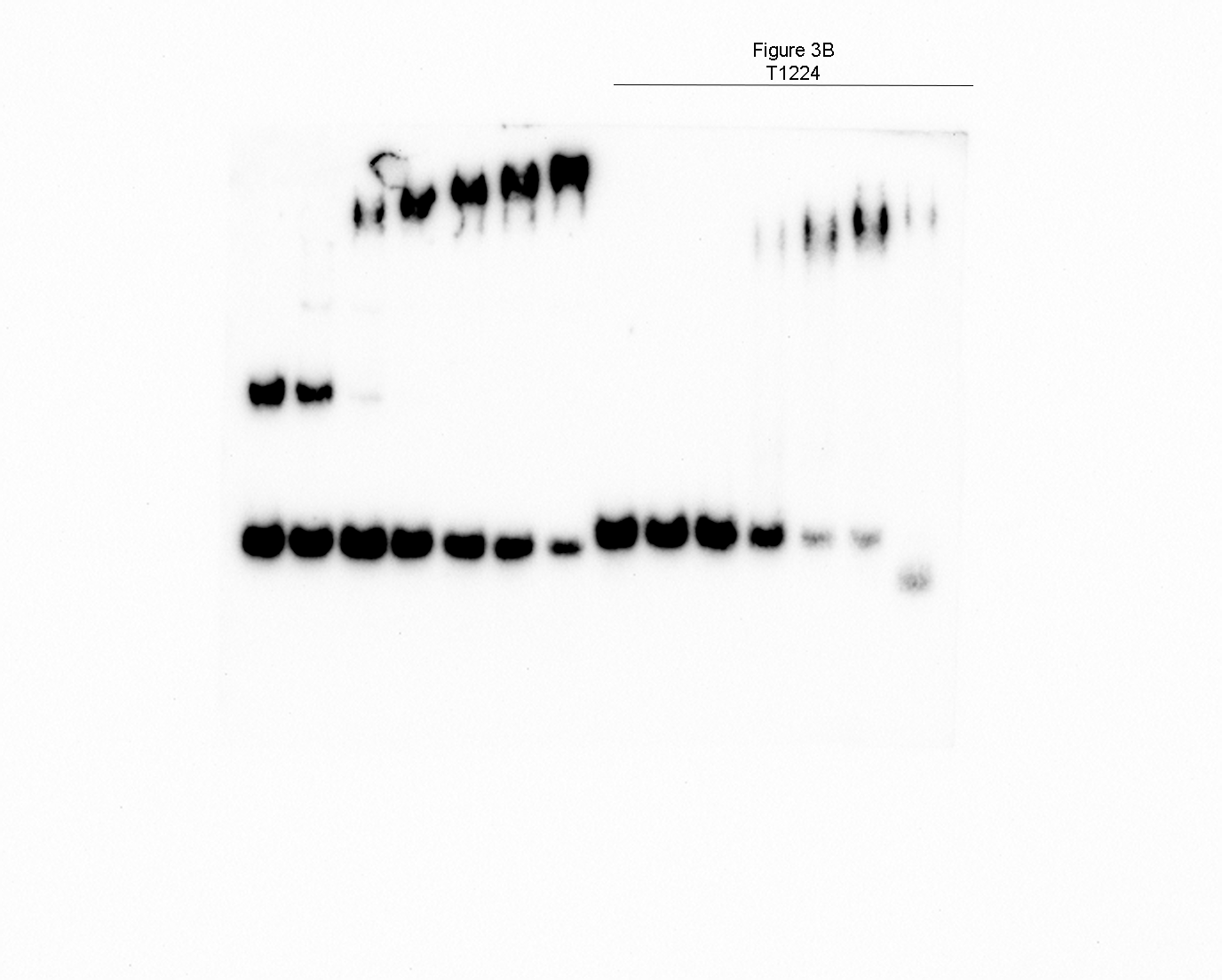

Supplement: Figure 3—source data 1. [file elife-70464-fig3-data1.zip › Figure 3-source data 1/Figure 3B-T1224-Labeled.tif]

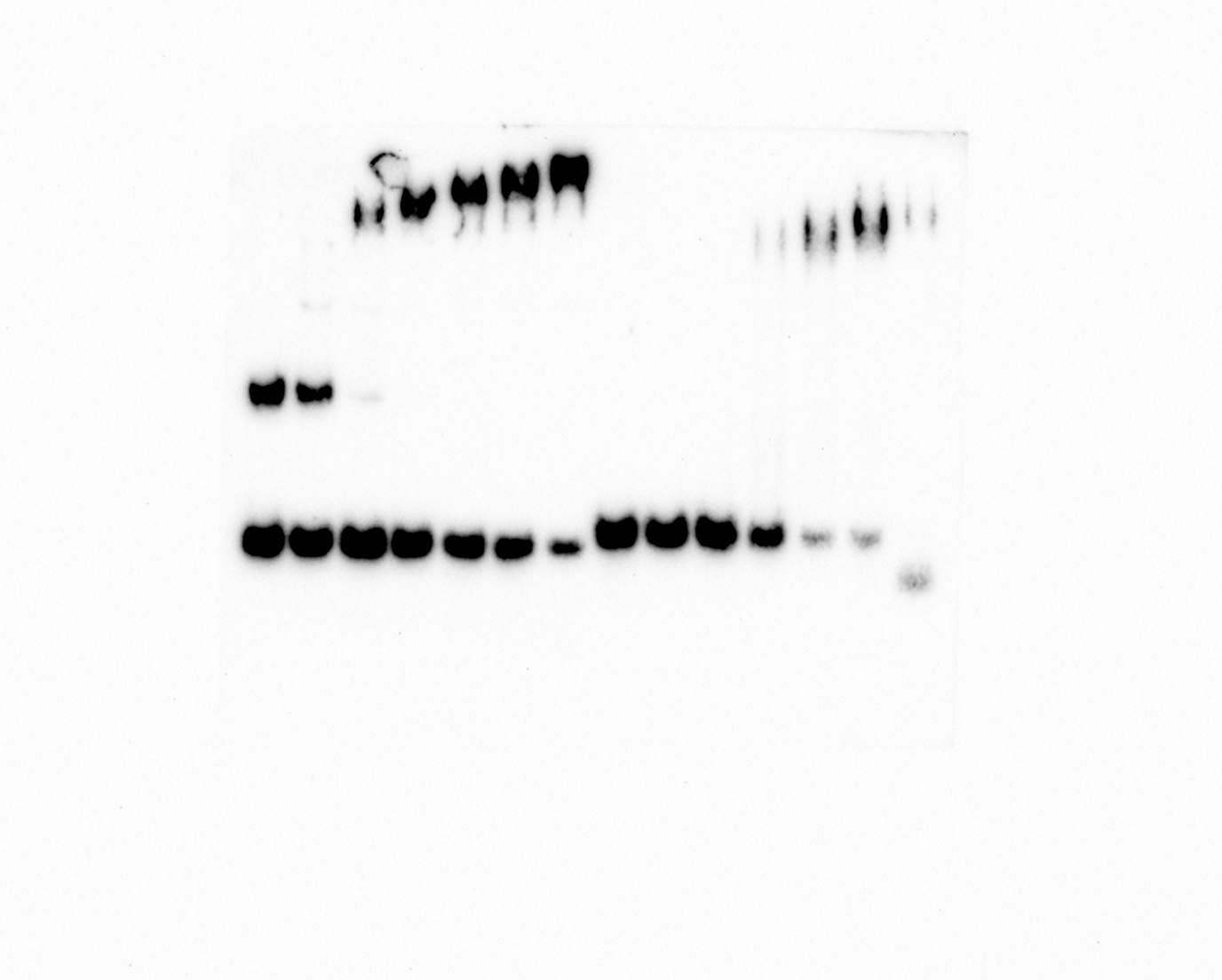

Supplement: Figure 3—source data 1. [file elife-70464-fig3-data1.zip › Figure 3-source data 1/Figure 3B-T1224-Original.tif]

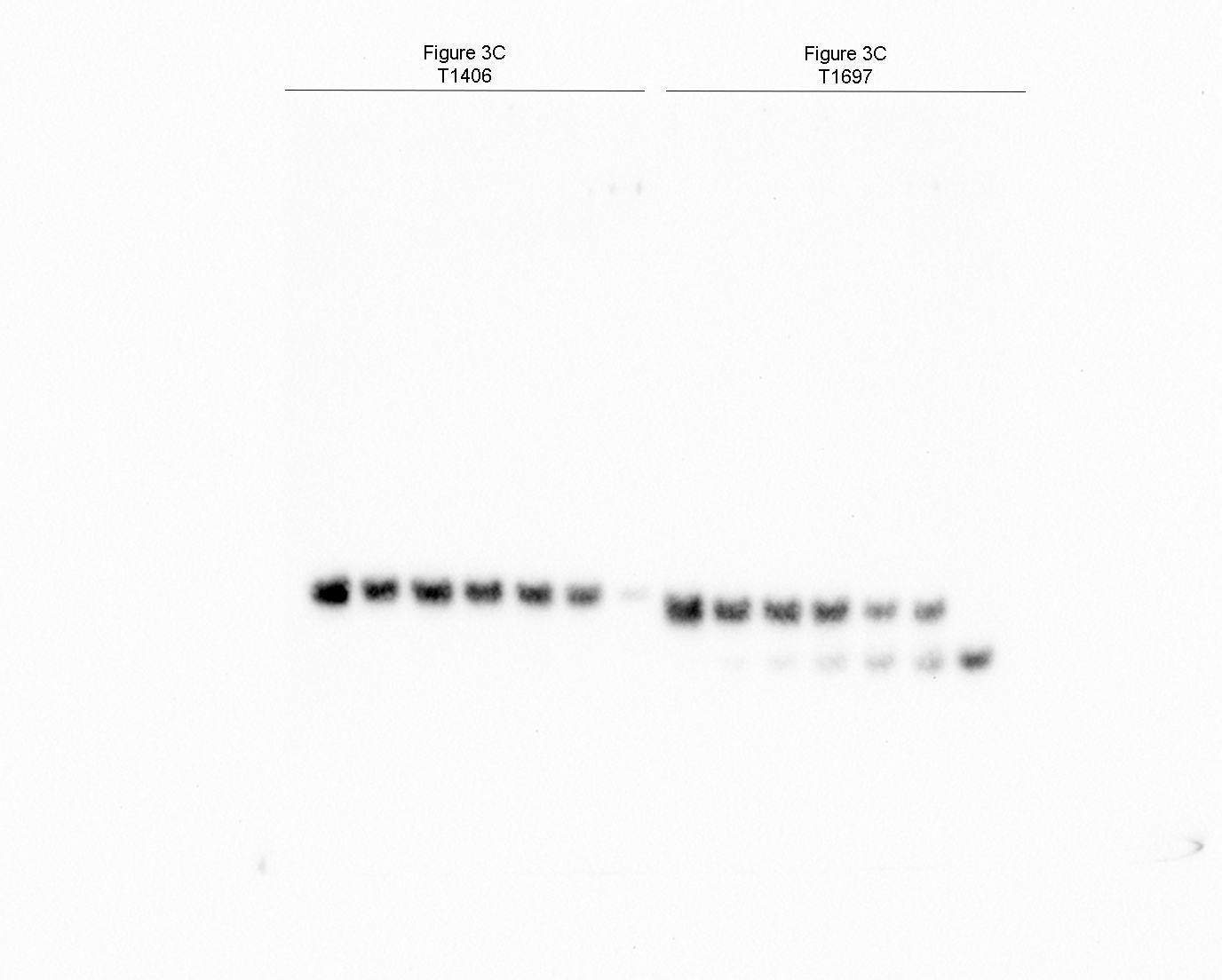

Supplement: Figure 3—source data 1. [file elife-70464-fig3-data1.zip › Figure 3-source data 1/Figure 3C-T1406 T1697-Labeled.tif]

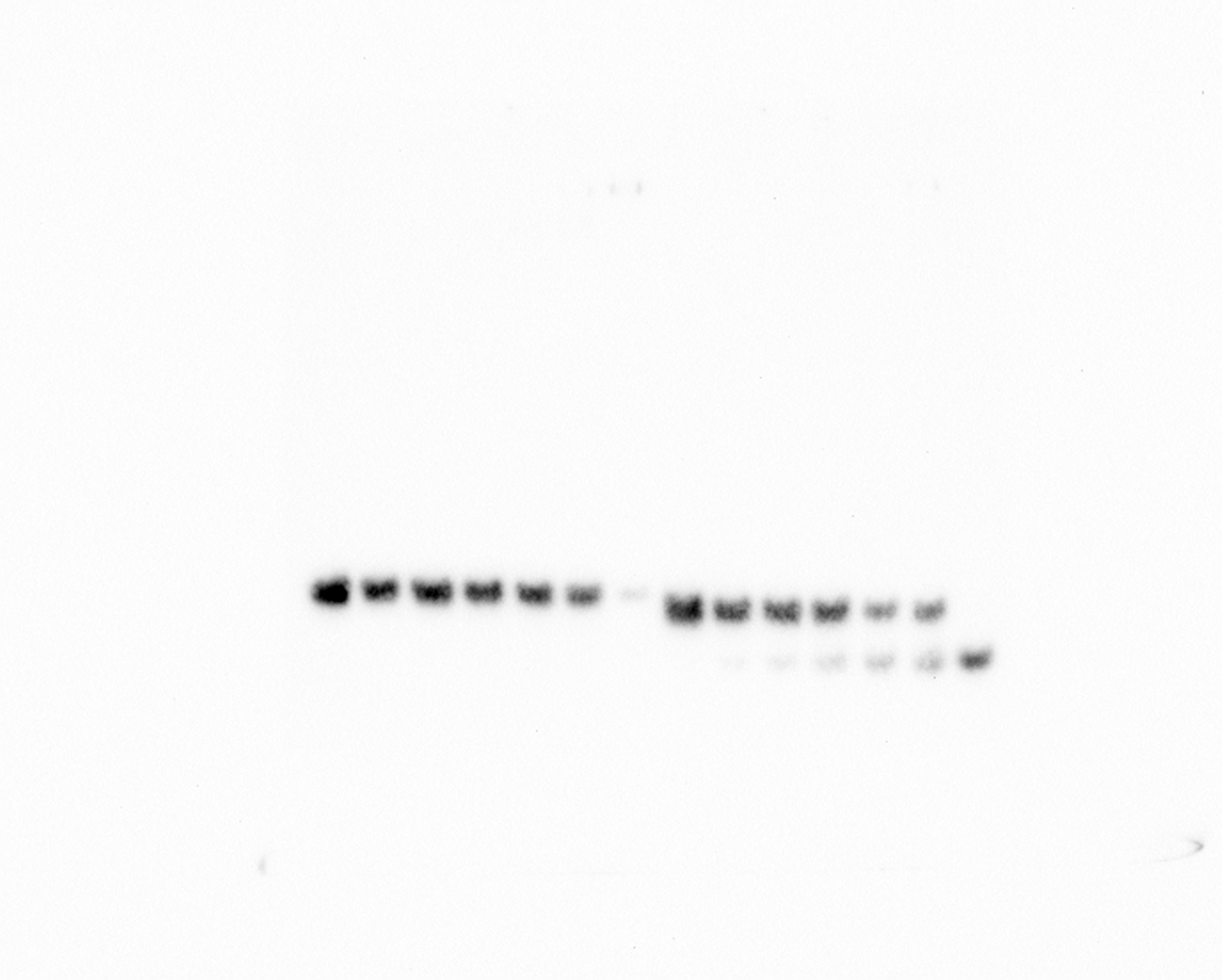

Supplement: Figure 3—source data 1. [file elife-70464-fig3-data1.zip › Figure 3-source data 1/Figure 3C-T1406 T1697-Original.tif]

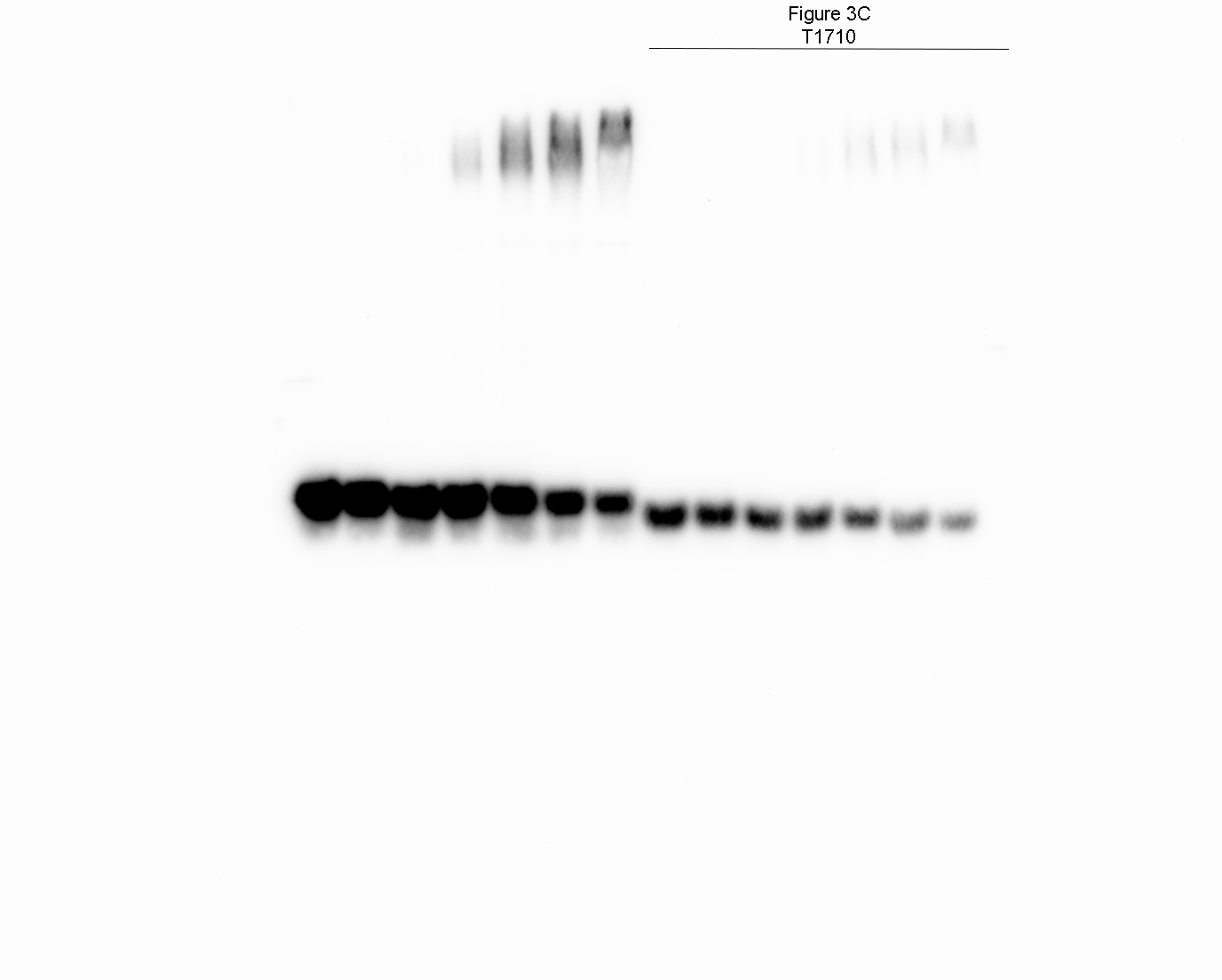

Supplement: Figure 3—source data 1. [file elife-70464-fig3-data1.zip › Figure 3-source data 1/Figure 3C-T1710-Labeled.tif]

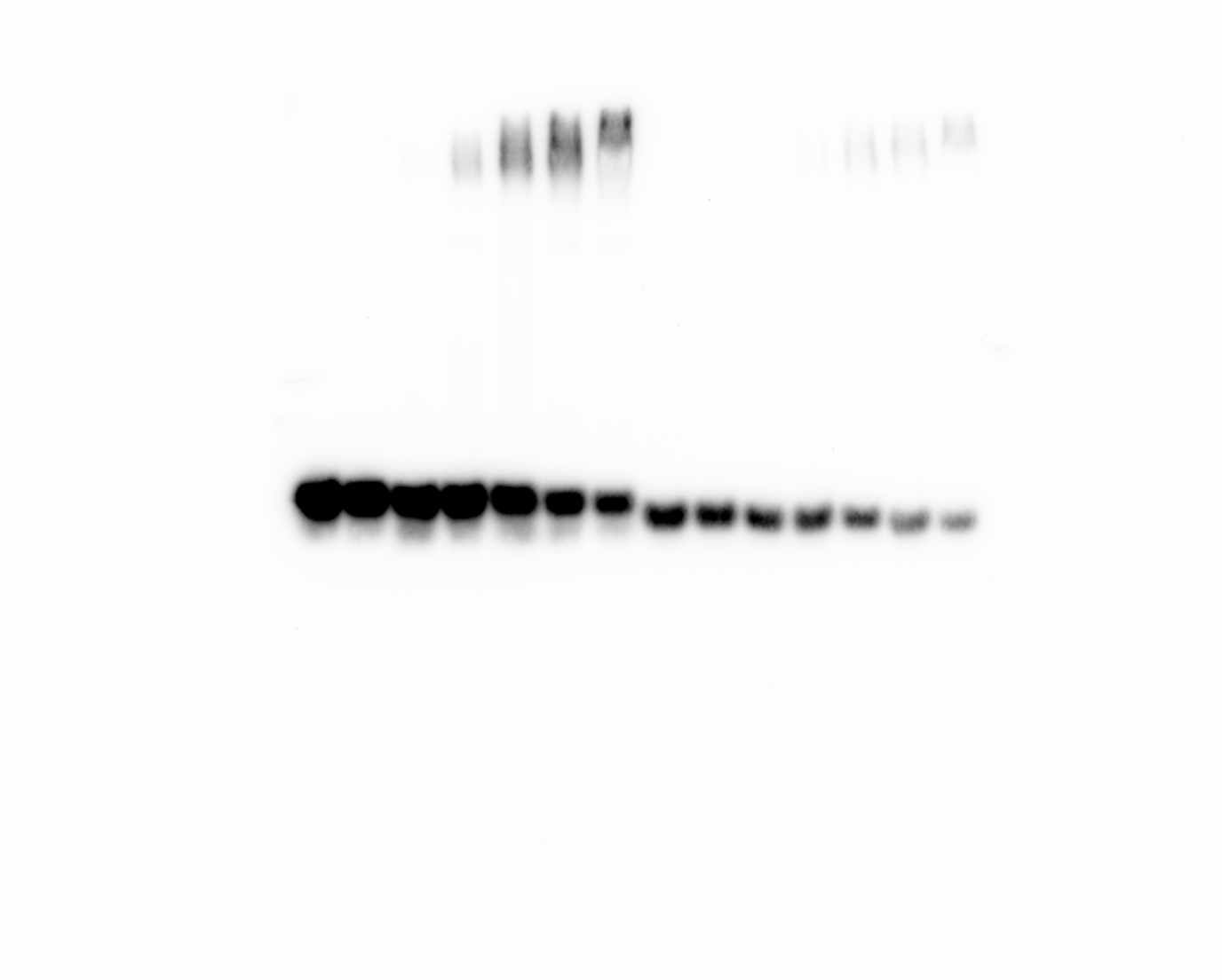

Supplement: Figure 3—source data 1. [file elife-70464-fig3-data1.zip › Figure 3-source data 1/Figure 3C-T1710-Original.tif]

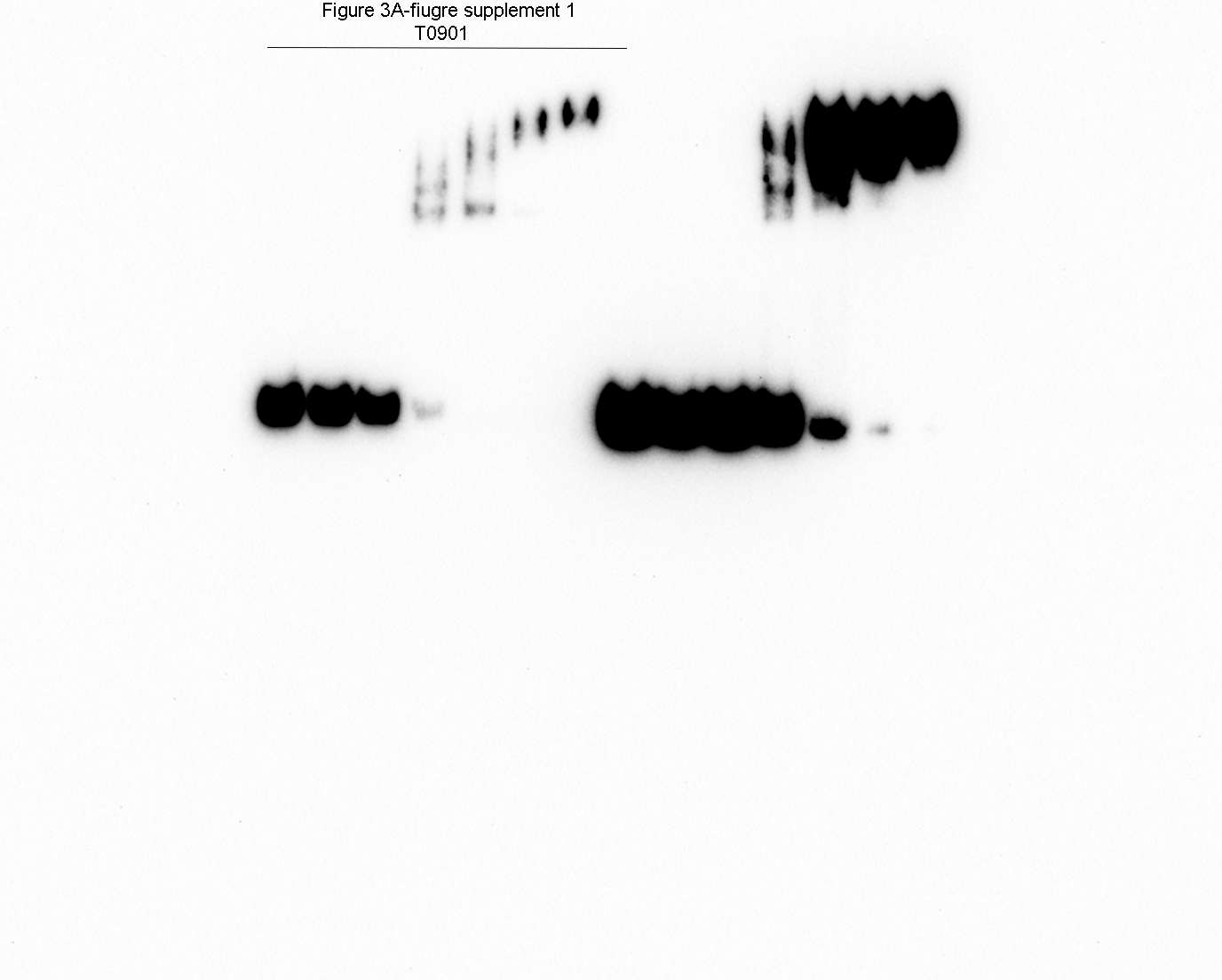

Supplement: Figure 3—figure supplement 1—source data 1. [file elife-70464-fig3-figsupp1-data1.zip › Figure 3-figure supplement 1-source data 1/Figure 3-figure supplement 1-T0901-Labeled.tif]

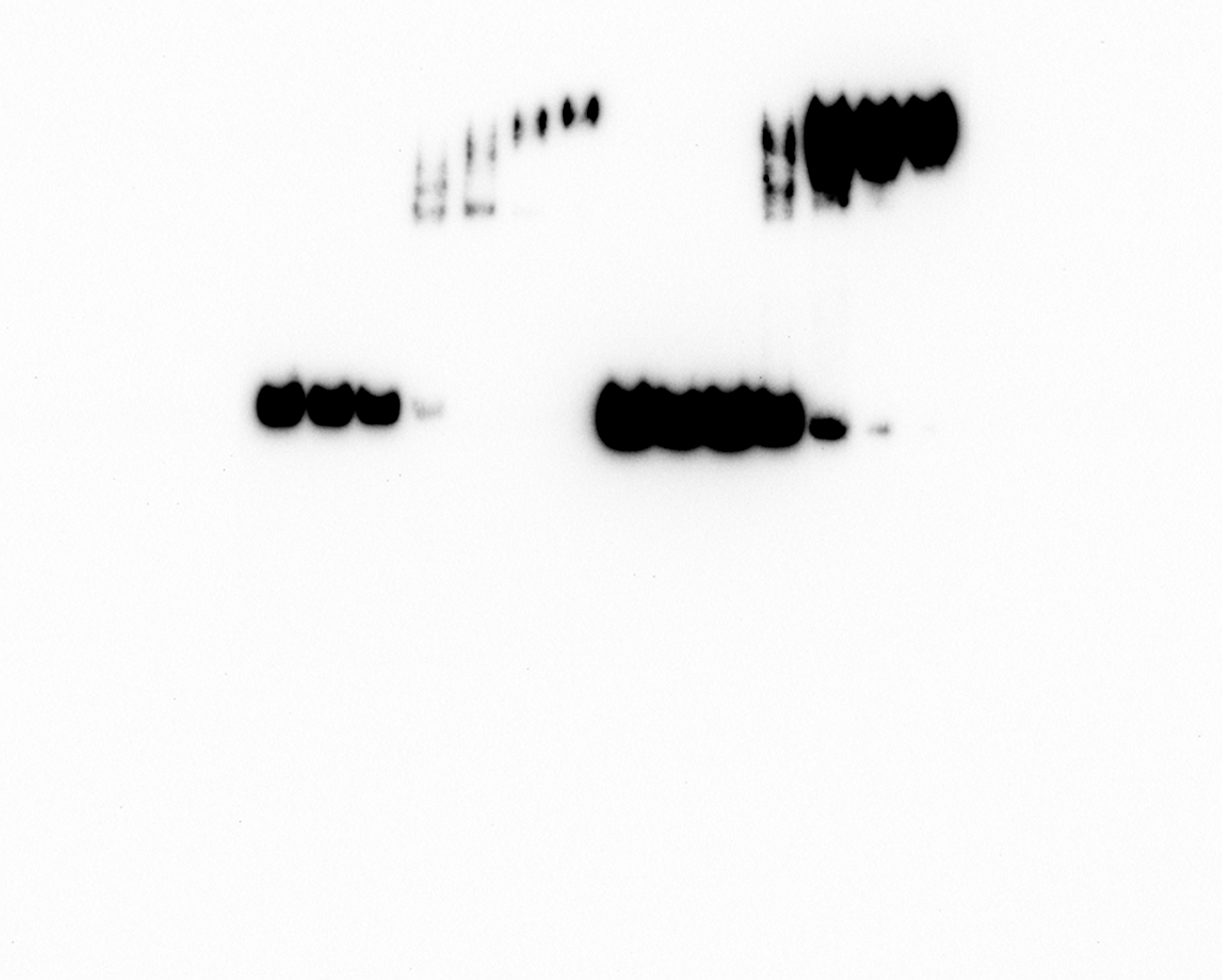

Supplement: Figure 3—figure supplement 1—source data 1. [file elife-70464-fig3-figsupp1-data1.zip › Figure 3-figure supplement 1-source data 1/Figure 3-figure supplement 1-T0901-Original.tif]

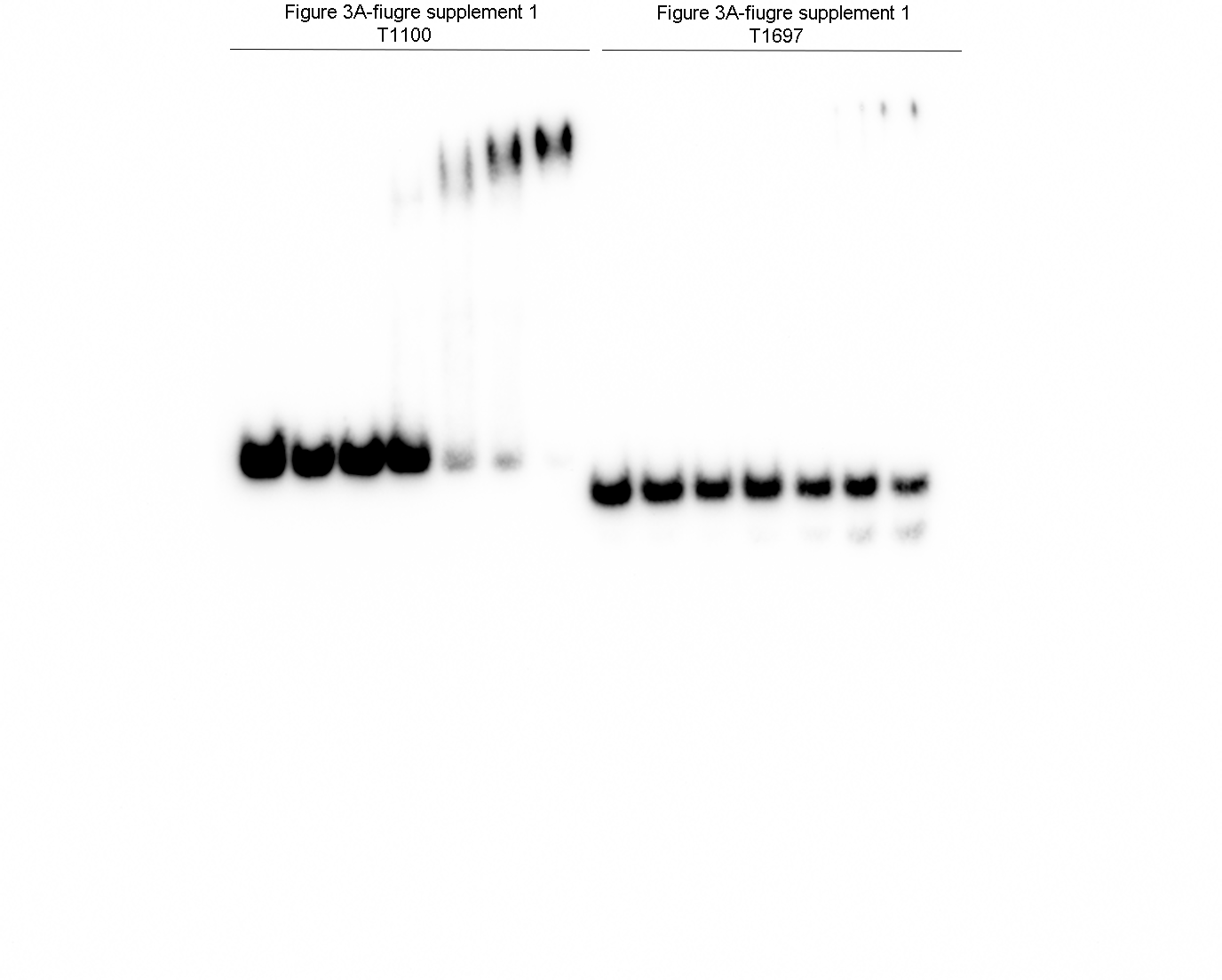

Supplement: Figure 3—figure supplement 1—source data 1. [file elife-70464-fig3-figsupp1-data1.zip › Figure 3-figure supplement 1-source data 1/Figure 3-figure supplement 1-T1100 T1697-Labeled.tif]

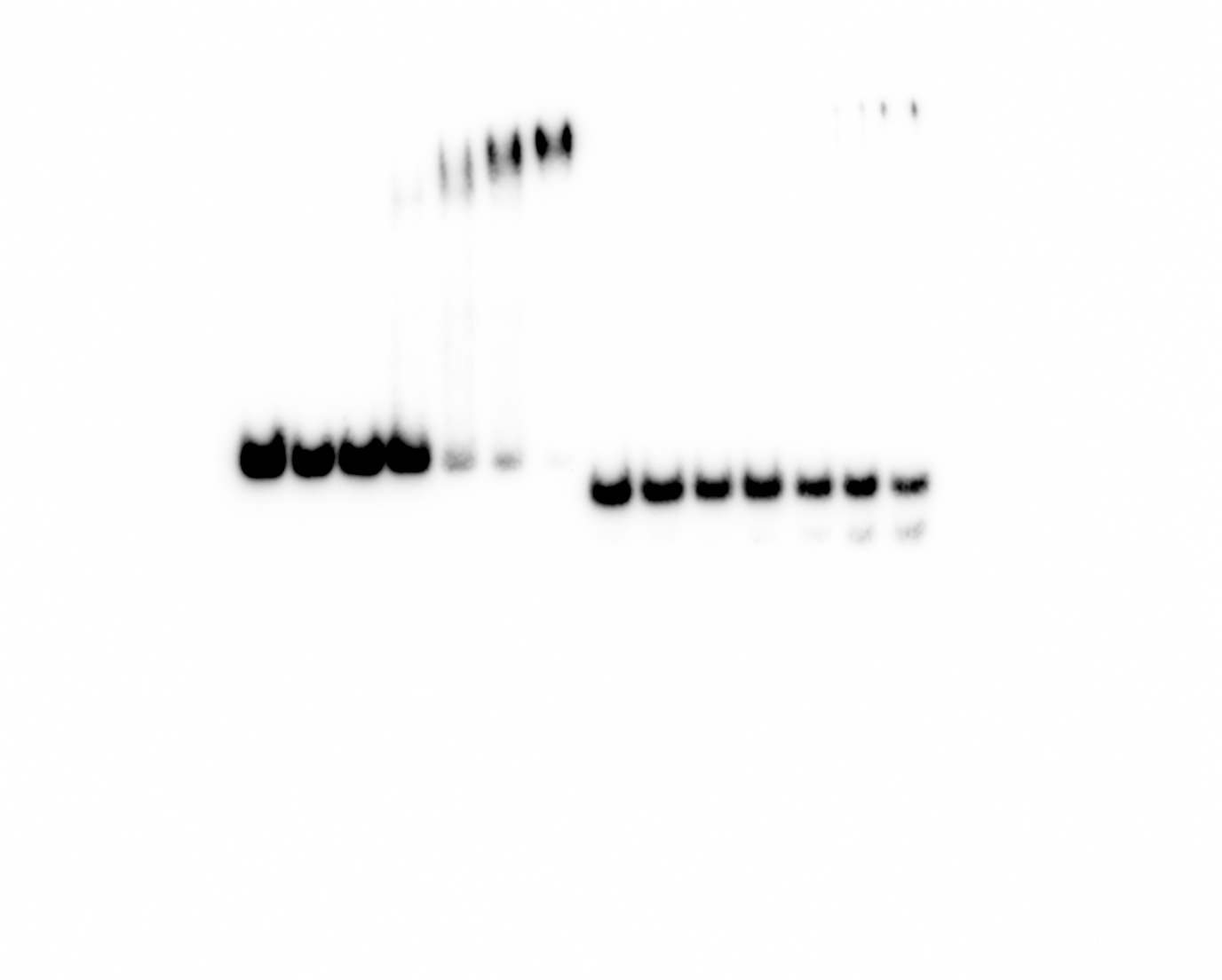

Supplement: Figure 3—figure supplement 1—source data 1. [file elife-70464-fig3-figsupp1-data1.zip › Figure 3-figure supplement 1-source data 1/Figure 3-figure supplement 1-T1100 T1697-Original.tif]

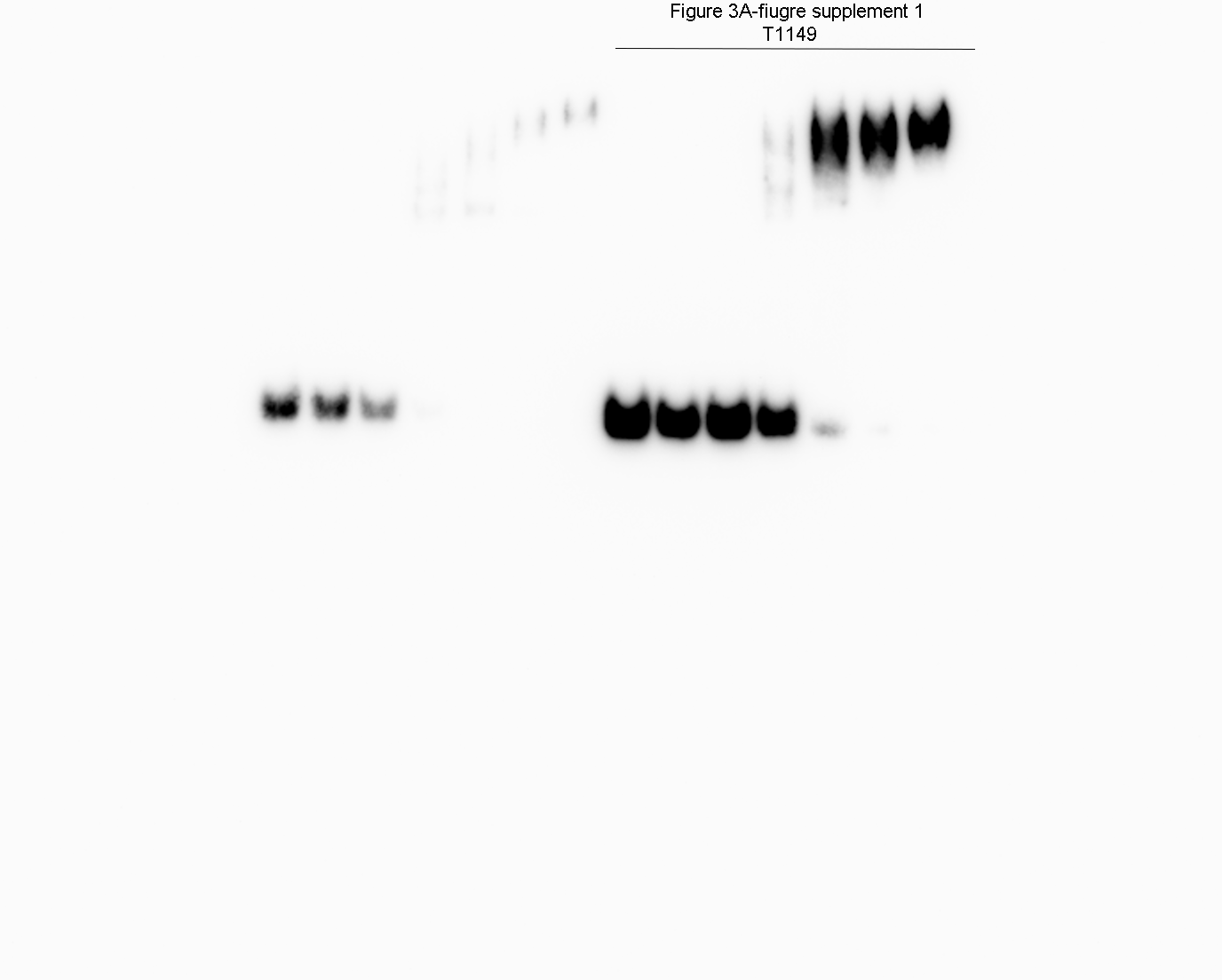

Supplement: Figure 3—figure supplement 1—source data 1. [file elife-70464-fig3-figsupp1-data1.zip › Figure 3-figure supplement 1-source data 1/Figure 3-figure supplement 1-T1149-Labeled.tif]

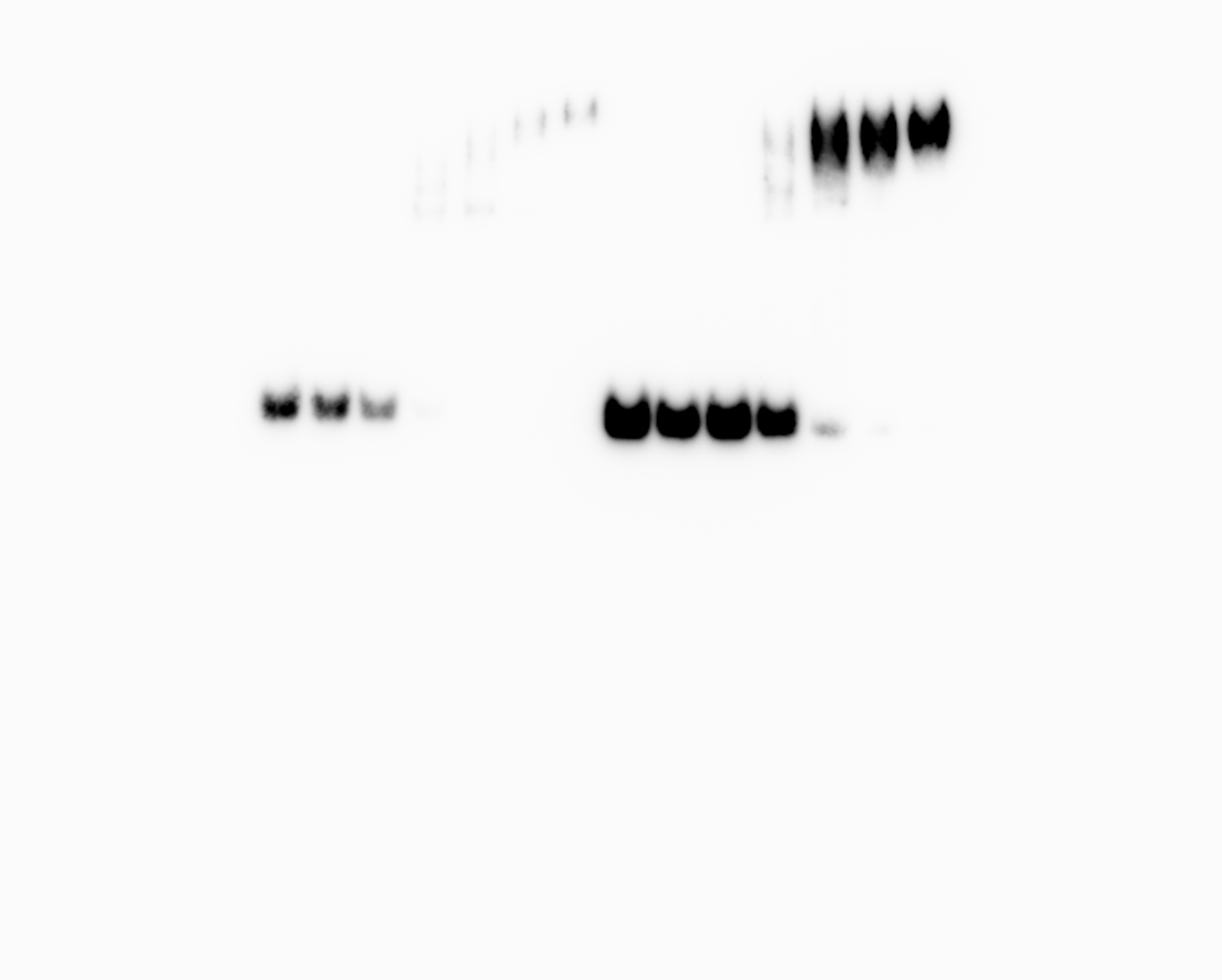

Supplement: Figure 3—figure supplement 1—source data 1. [file elife-70464-fig3-figsupp1-data1.zip › Figure 3-figure supplement 1-source data 1/Figure 3-figure supplement 1-T1149-Original.tif]

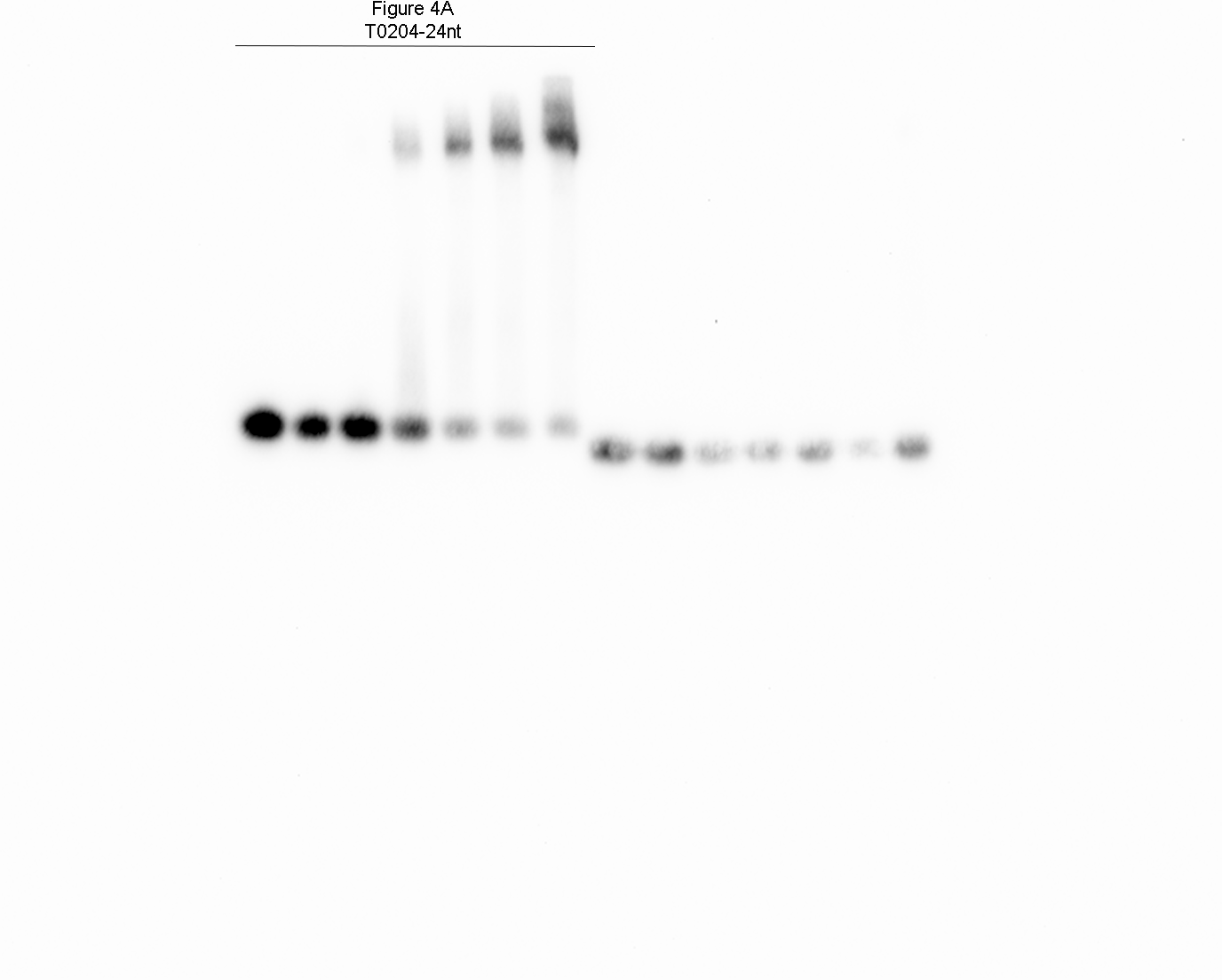

Supplement: Figure 4—source data 1. [file elife-70464-fig4-data1.zip › Figure 4-source data 1/Figure 4A-T0204 24nt-Labeled.tif]

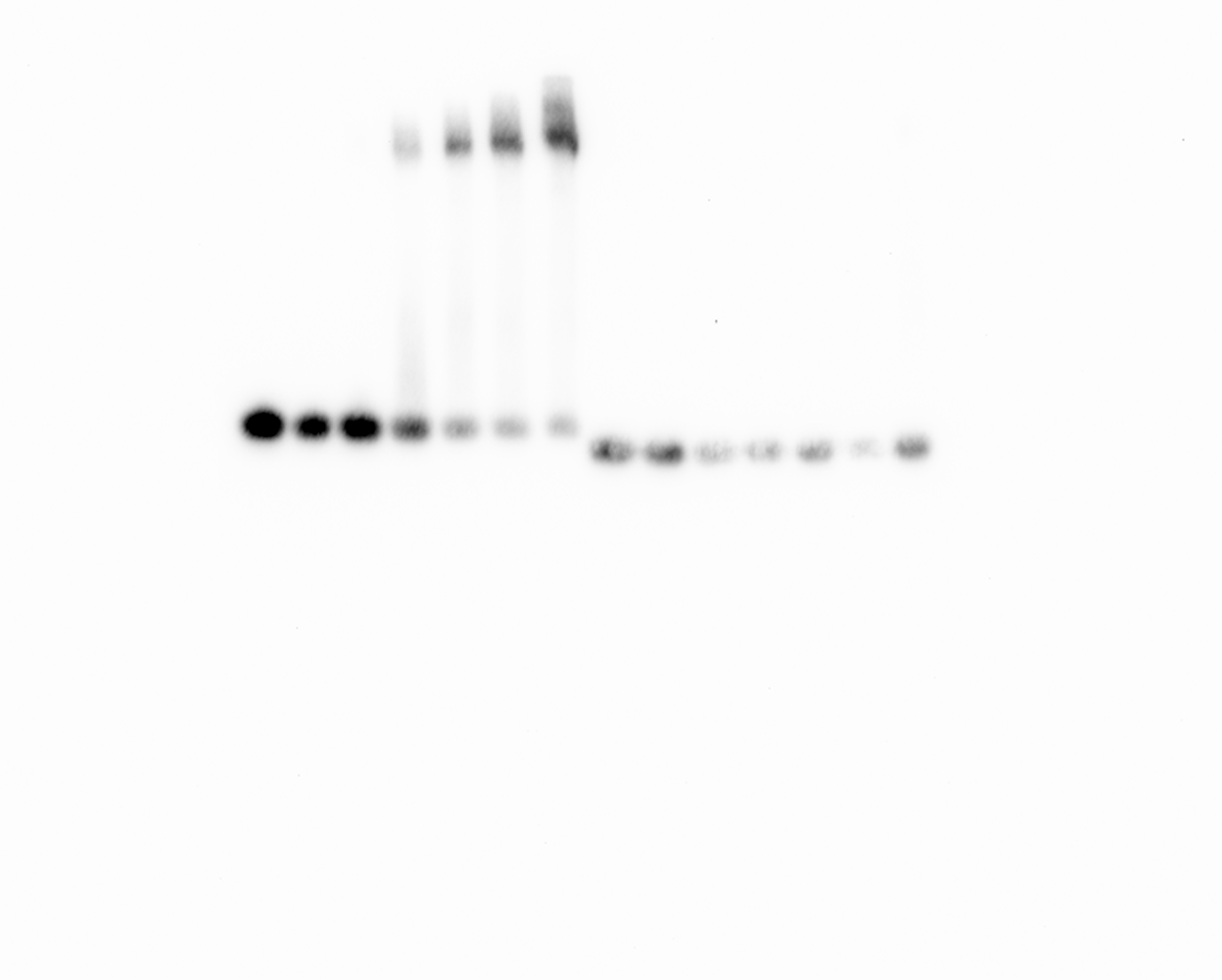

Supplement: Figure 4—source data 1. [file elife-70464-fig4-data1.zip › Figure 4-source data 1/Figure 4A-T0204 24nt-Original.tif]

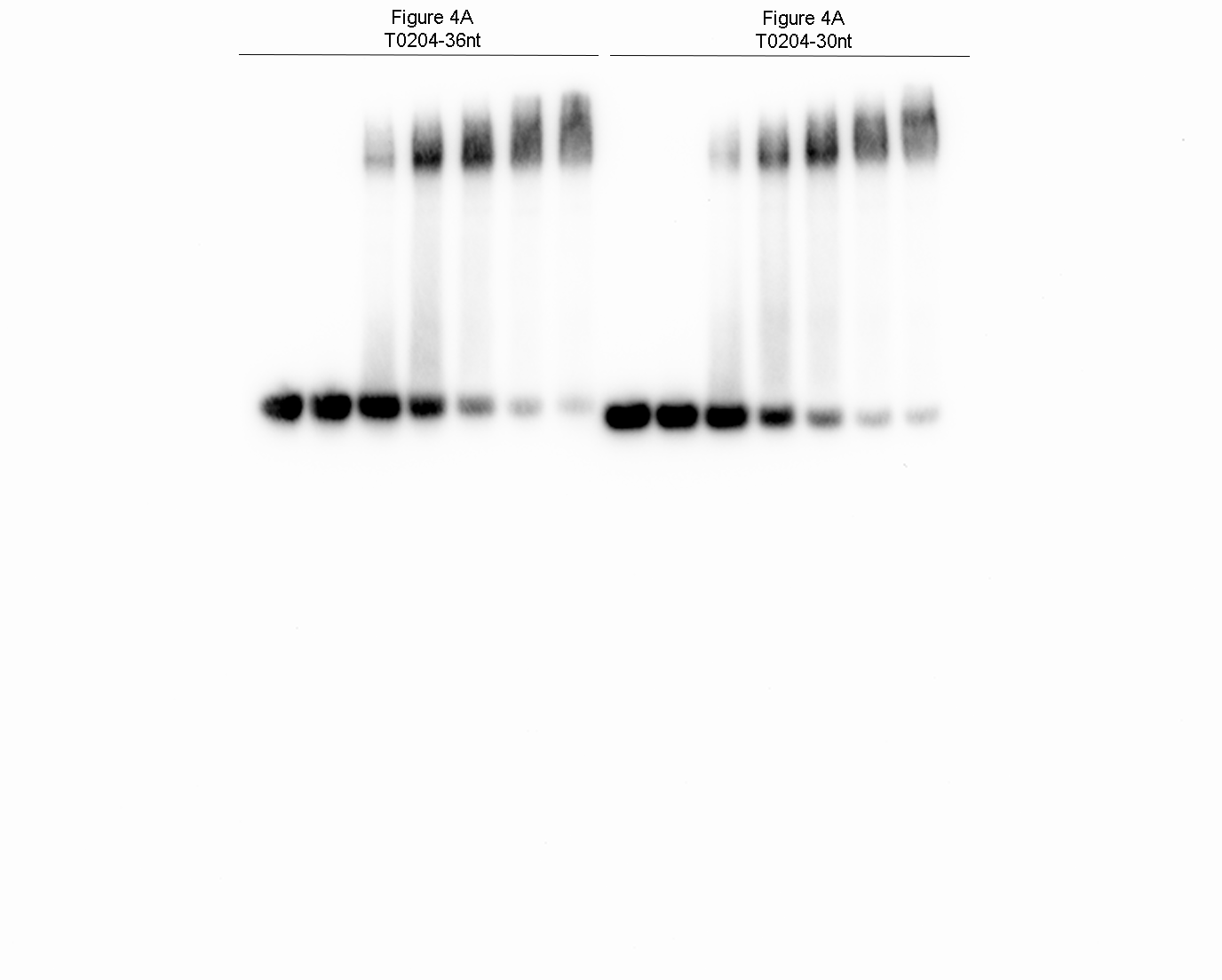

Supplement: Figure 4—source data 1. [file elife-70464-fig4-data1.zip › Figure 4-source data 1/Figure 4A-T0204 36nt 30nt-Labeled.tif]

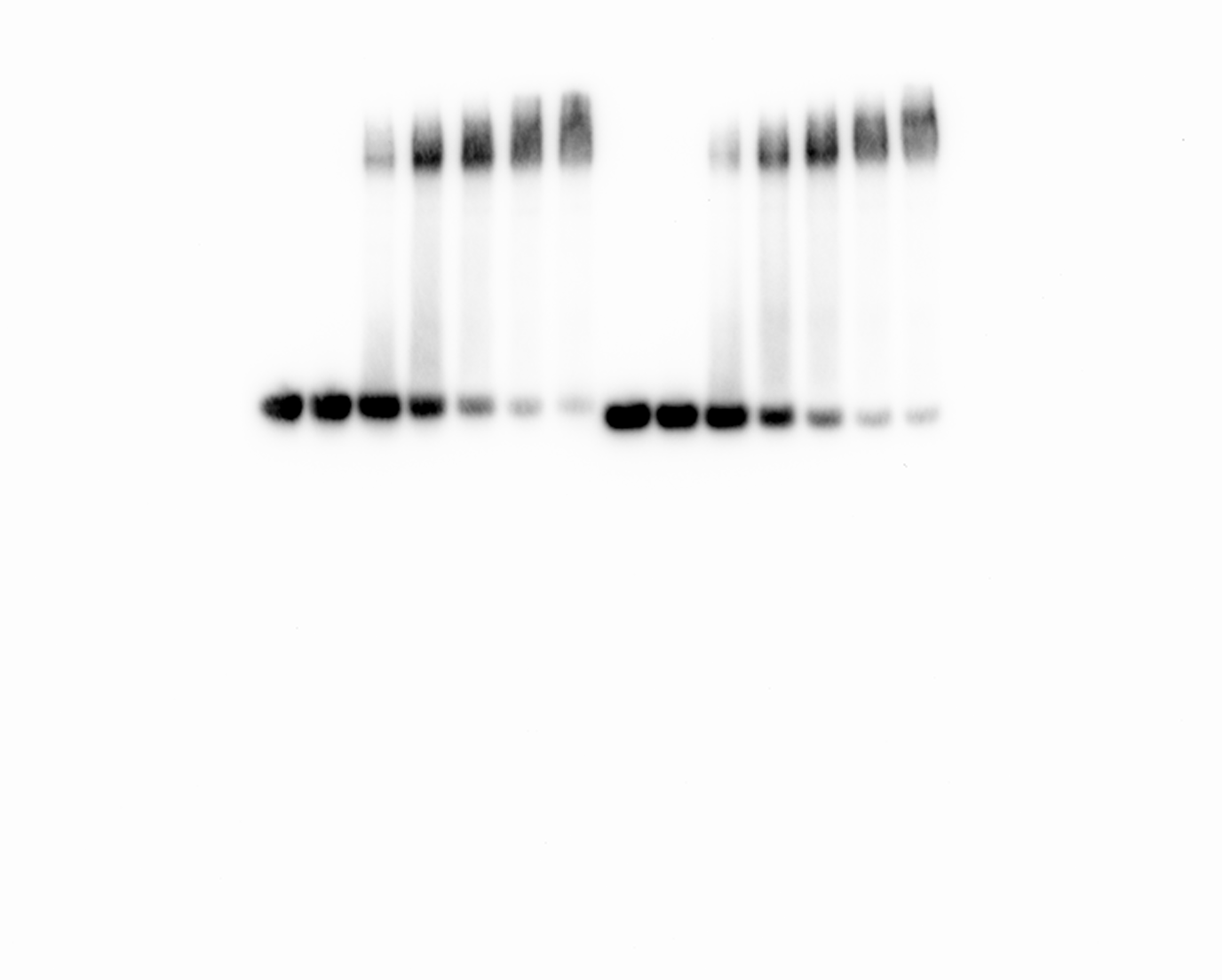

Supplement: Figure 4—source data 1. [file elife-70464-fig4-data1.zip › Figure 4-source data 1/Figure 4A-T0204 36nt 30nt-Original.tif]

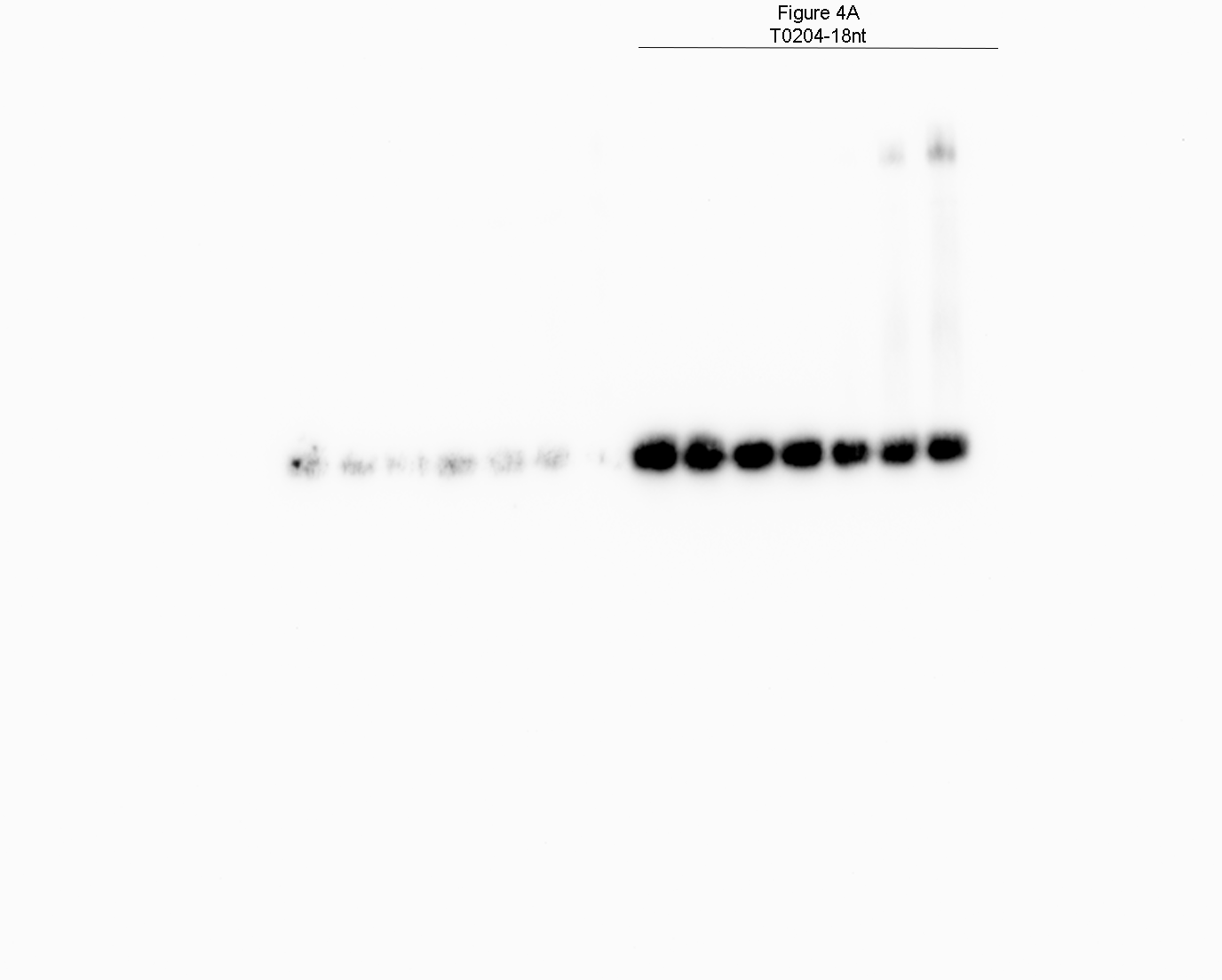

Supplement: Figure 4—source data 1. [file elife-70464-fig4-data1.zip › Figure 4-source data 1/Figure 4A-T0204-18nt-Labeled.tif]

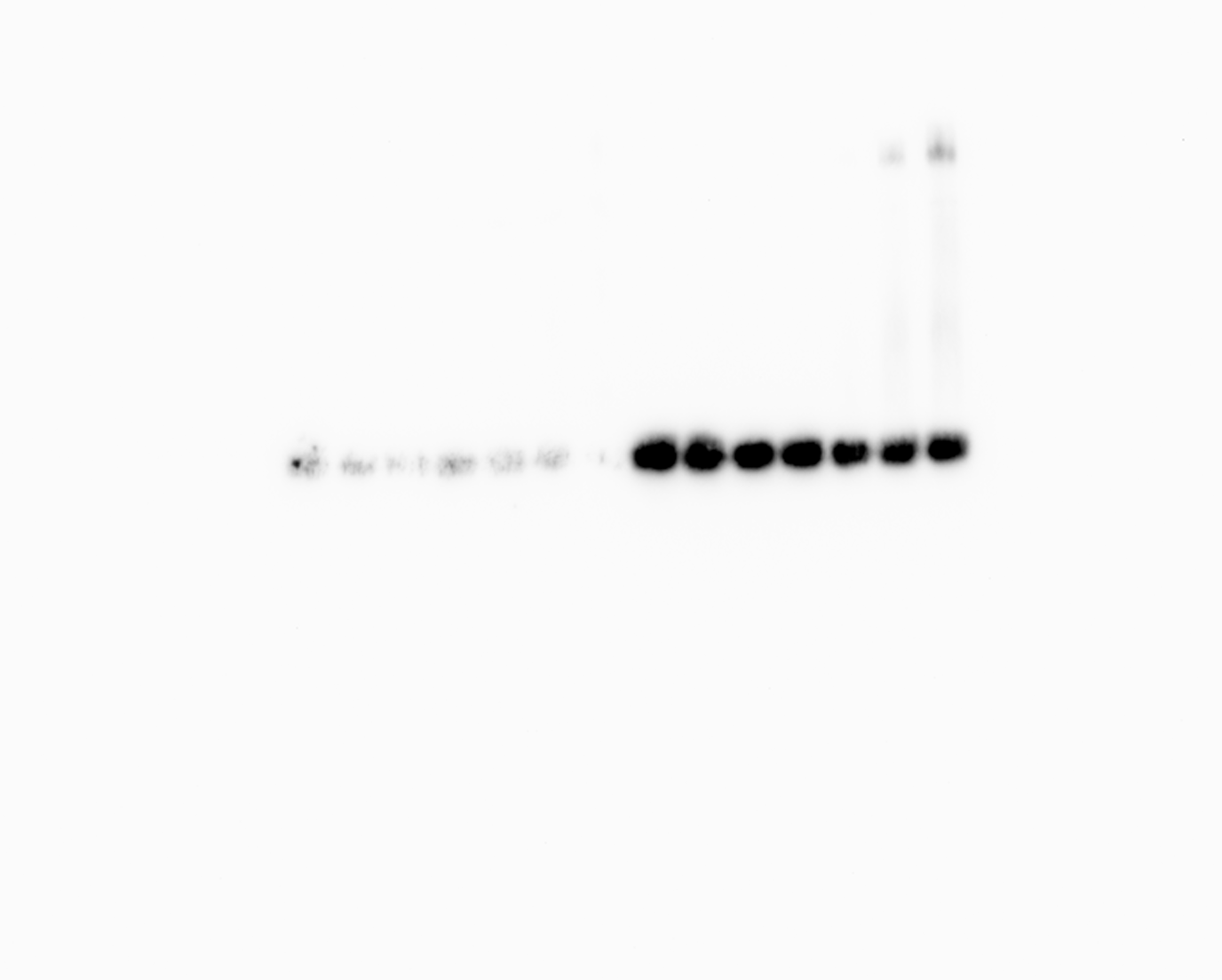

Supplement: Figure 4—source data 1. [file elife-70464-fig4-data1.zip › Figure 4-source data 1/Figure 4A-T0204-18nt-Original.tif]

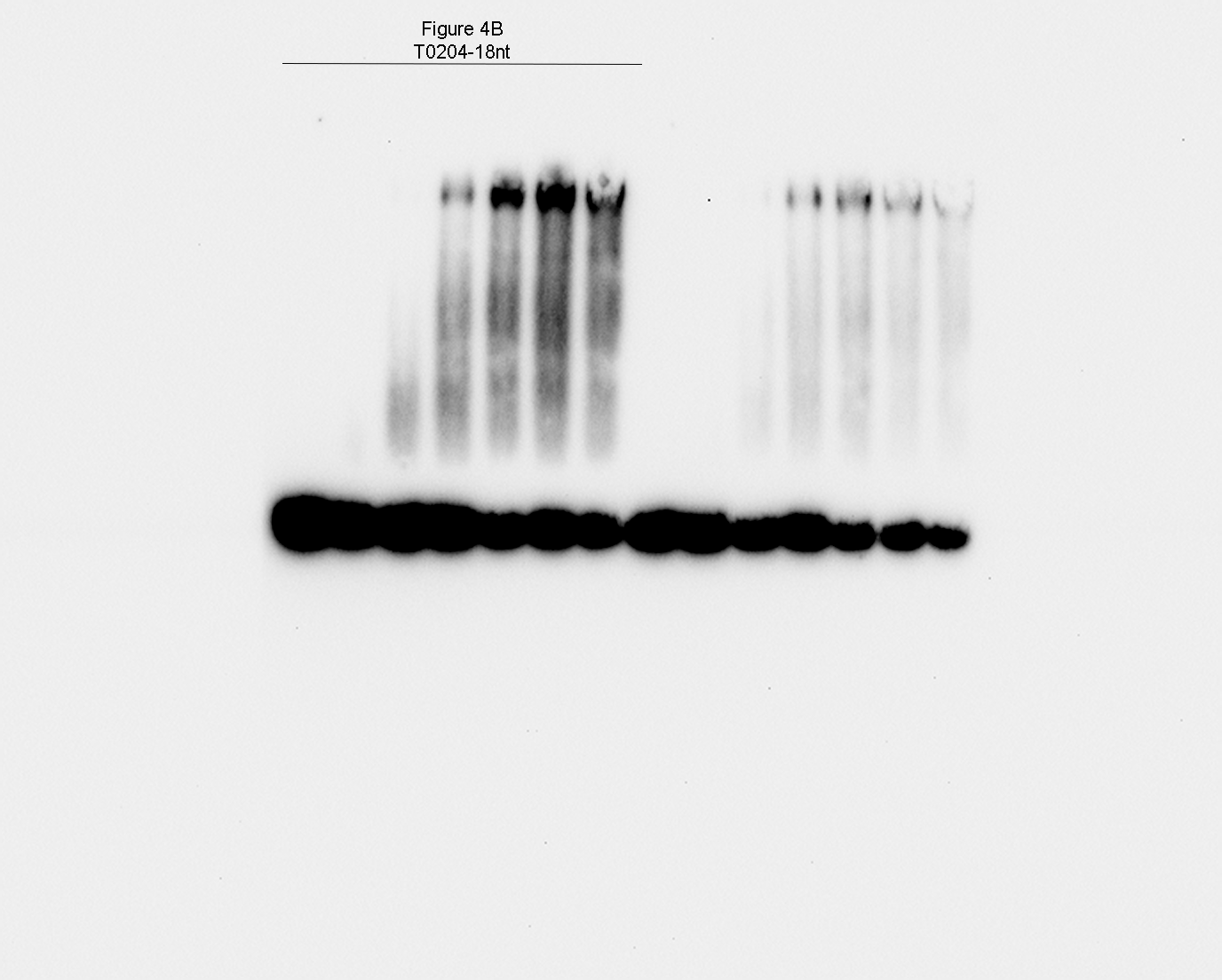

Supplement: Figure 4—source data 1. [file elife-70464-fig4-data1.zip › Figure 4-source data 1/Figure 4B-T0204-18nt- Labeled.tif]

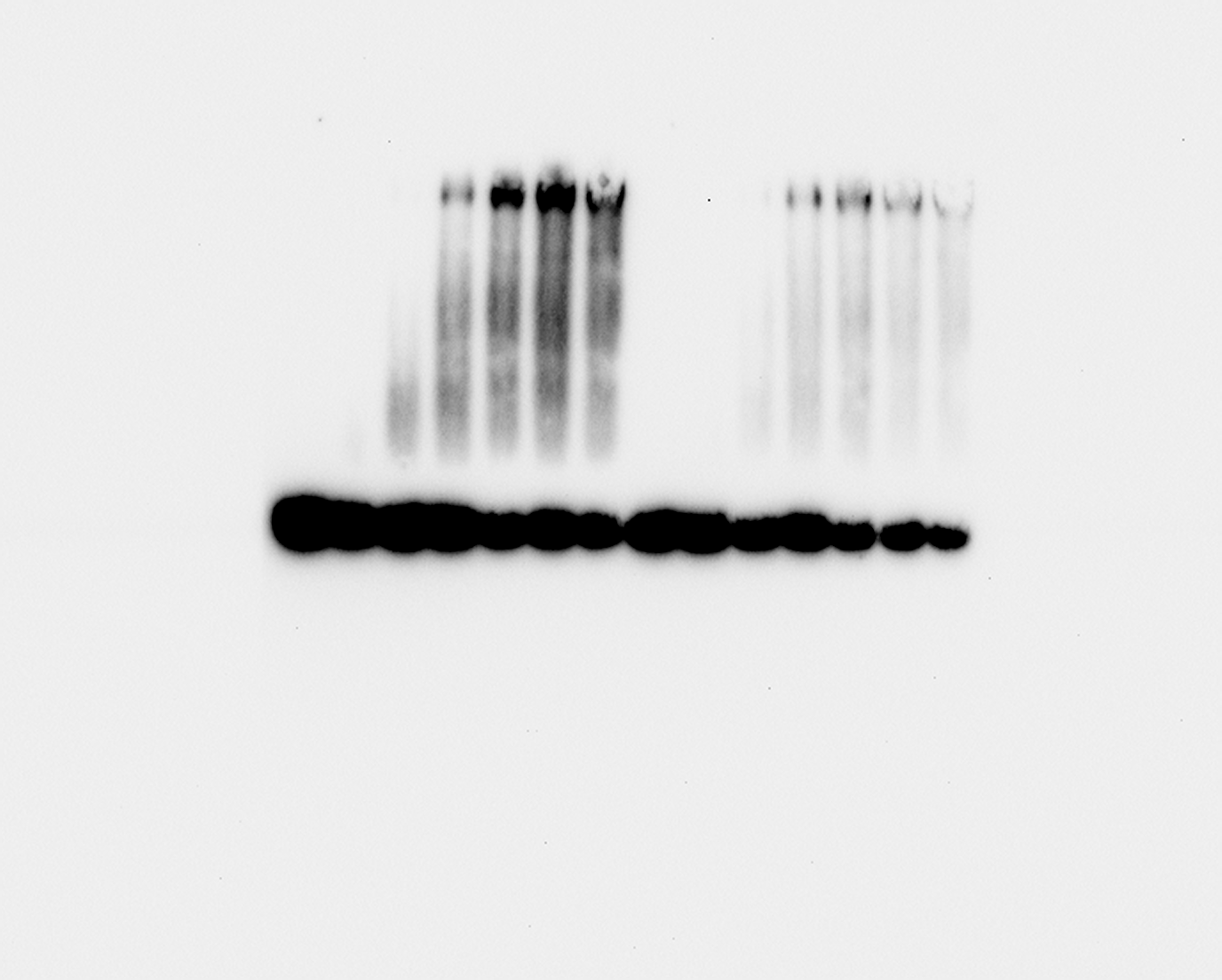

Supplement: Figure 4—source data 1. [file elife-70464-fig4-data1.zip › Figure 4-source data 1/Figure 4B-T0204-18nt- Original.tif]

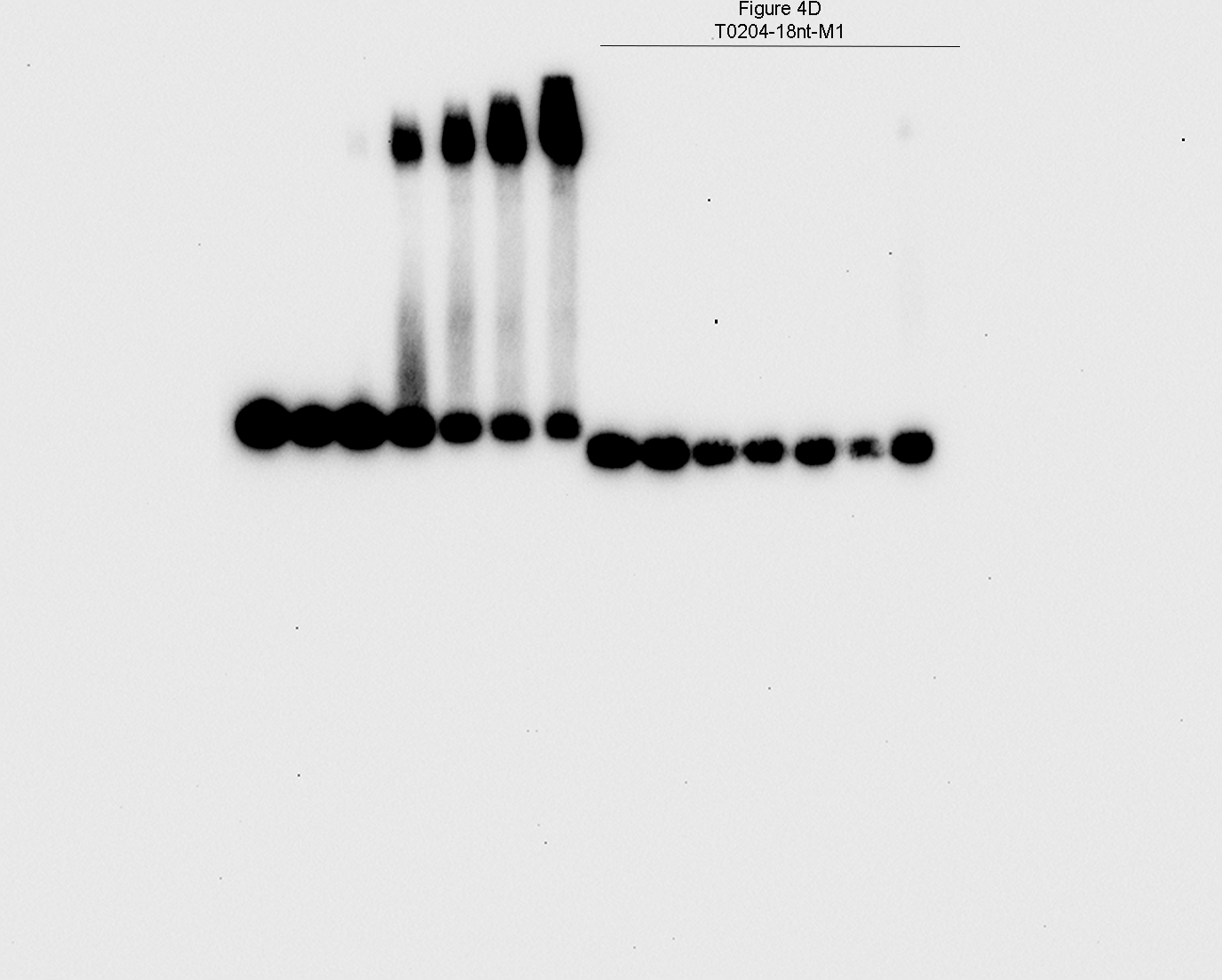

Supplement: Figure 4—source data 1. [file elife-70464-fig4-data1.zip › Figure 4-source data 1/Figure 4B-T0204-18nt-M1-Labeled.tif]

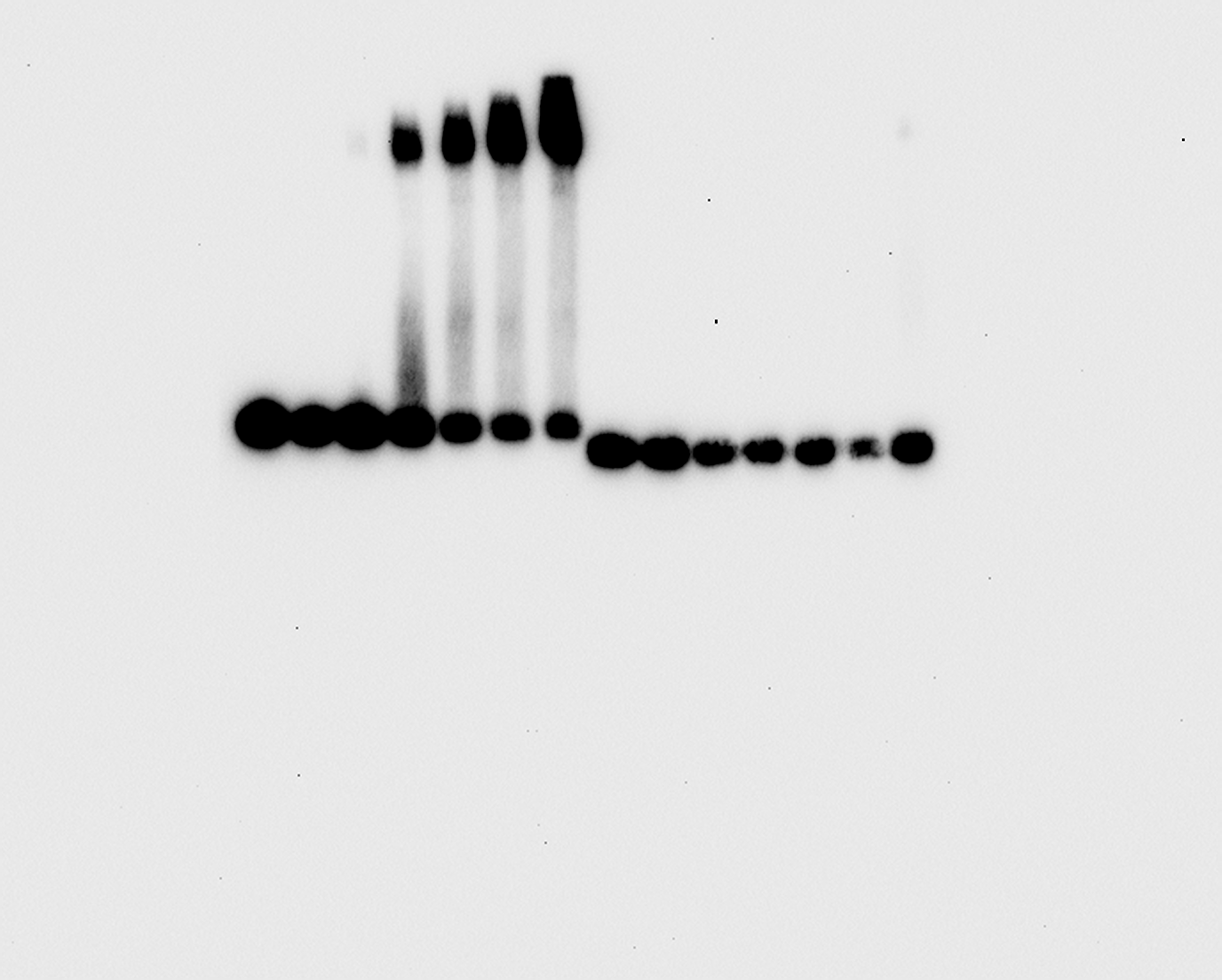

Supplement: Figure 4—source data 1. [file elife-70464-fig4-data1.zip › Figure 4-source data 1/Figure 4B-T0204-18nt-M1-Original.tif]

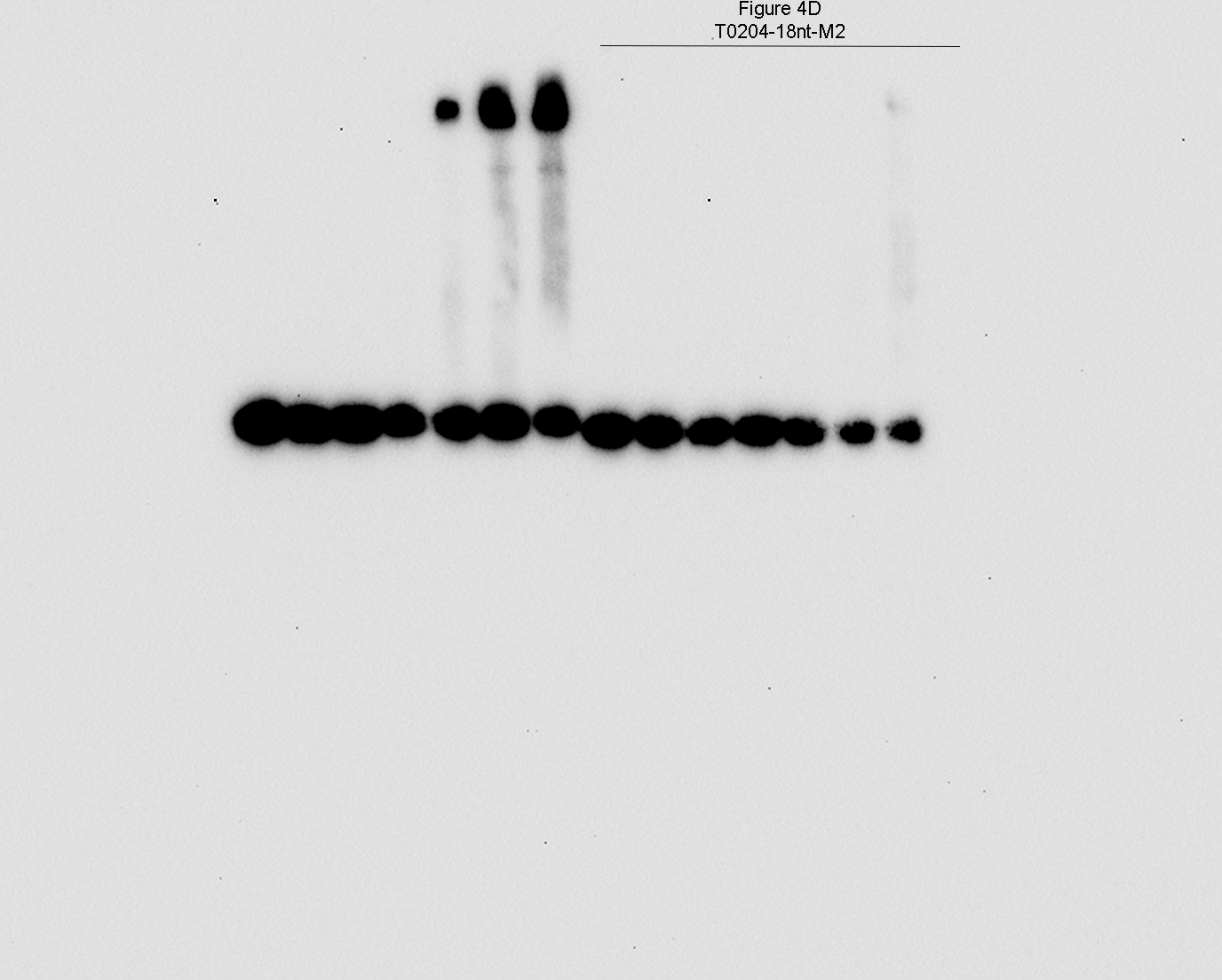

Supplement: Figure 4—source data 1. [file elife-70464-fig4-data1.zip › Figure 4-source data 1/Figure 4B-T0204-18nt-M2-Labeled.tif]

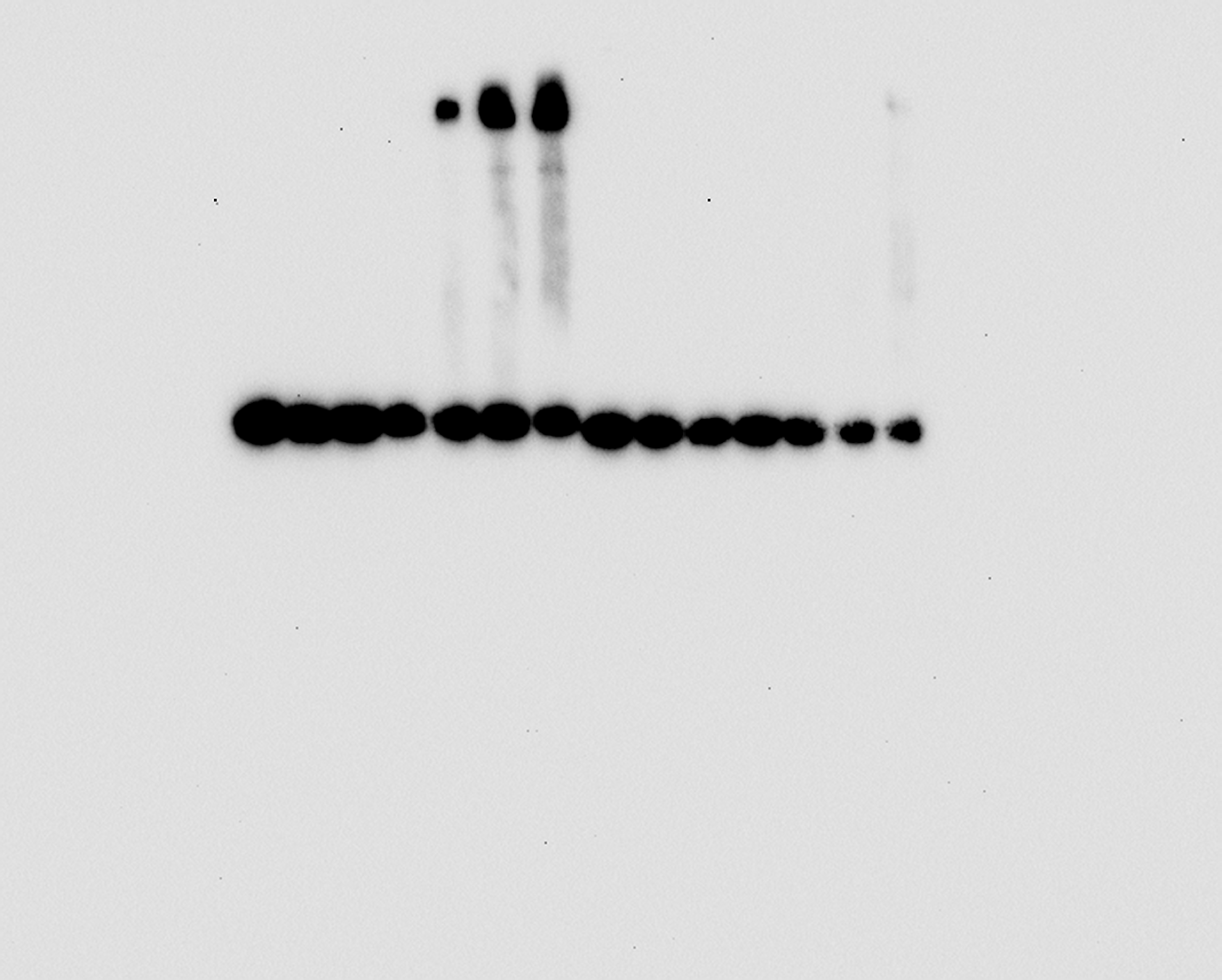

Supplement: Figure 4—source data 1. [file elife-70464-fig4-data1.zip › Figure 4-source data 1/Figure 4B-T0204-18nt-M2-Original.tif]

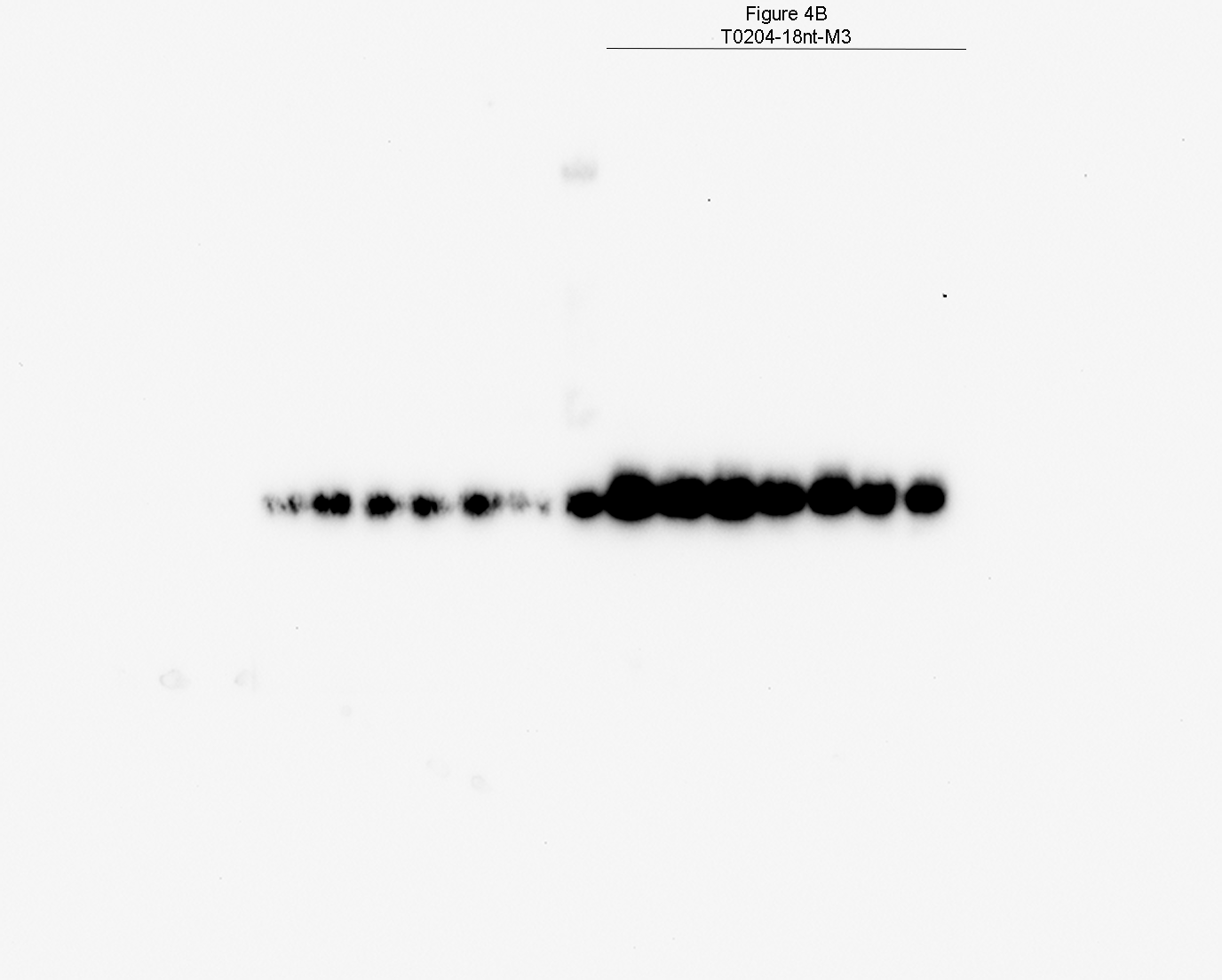

Supplement: Figure 4—source data 1. [file elife-70464-fig4-data1.zip › Figure 4-source data 1/Figure 4B-T0204-18nt-M3-Labeled.tif]

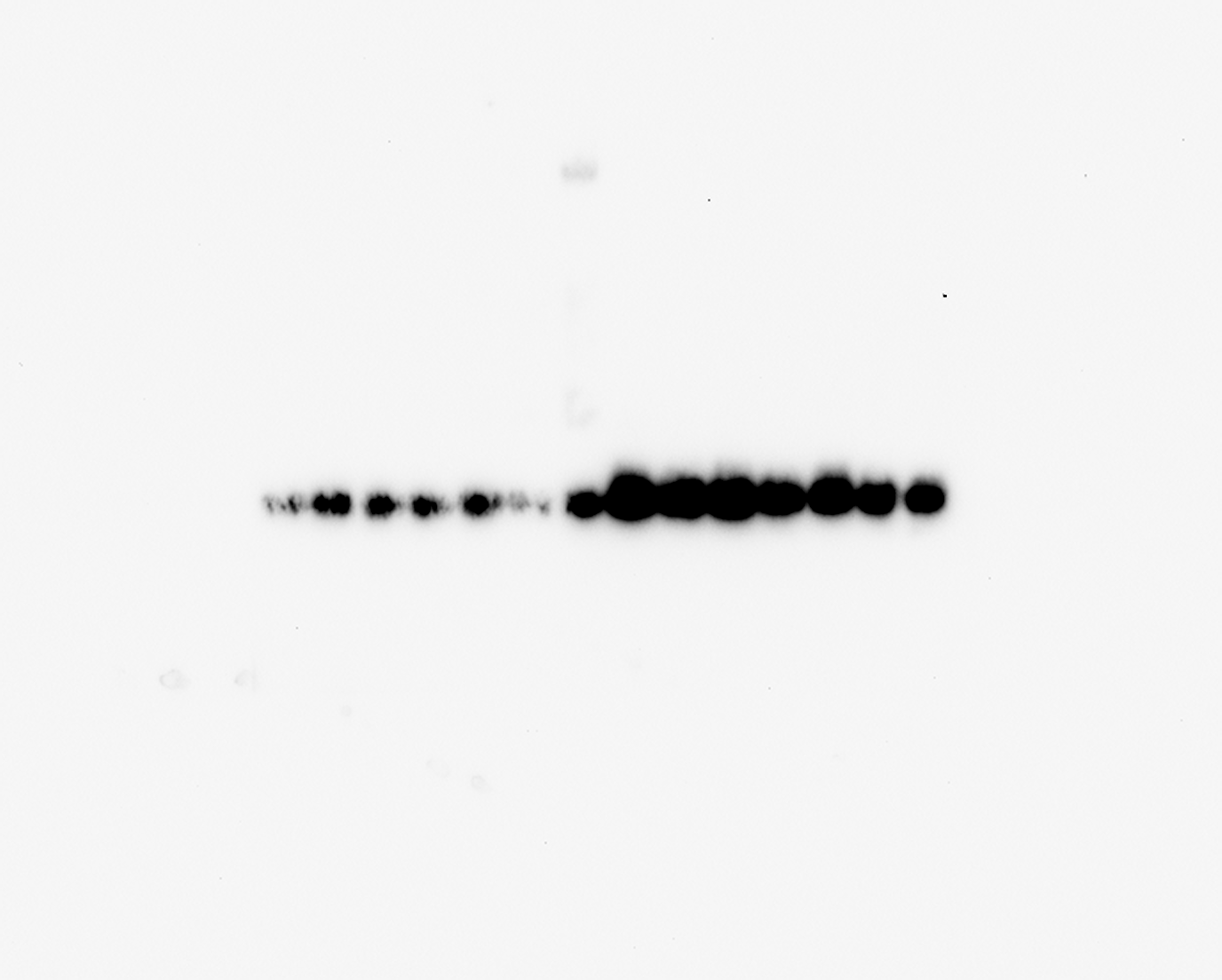

Supplement: Figure 4—source data 1. [file elife-70464-fig4-data1.zip › Figure 4-source data 1/Figure 4B-T0204-18nt-M3-Original.tif]

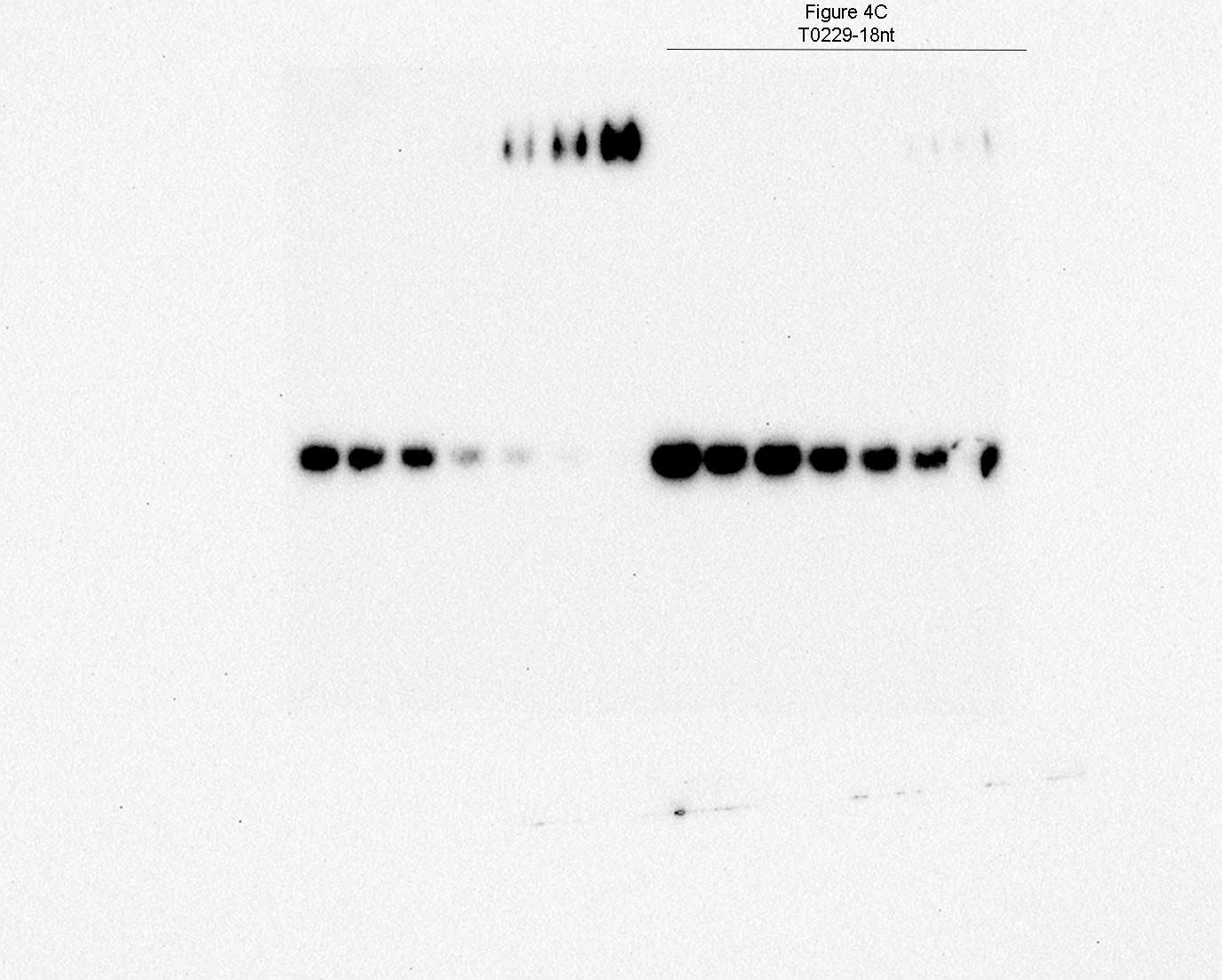

Supplement: Figure 4—source data 1. [file elife-70464-fig4-data1.zip › Figure 4-source data 1/Figure 4C-T0229-18nt- Labeled.tif]

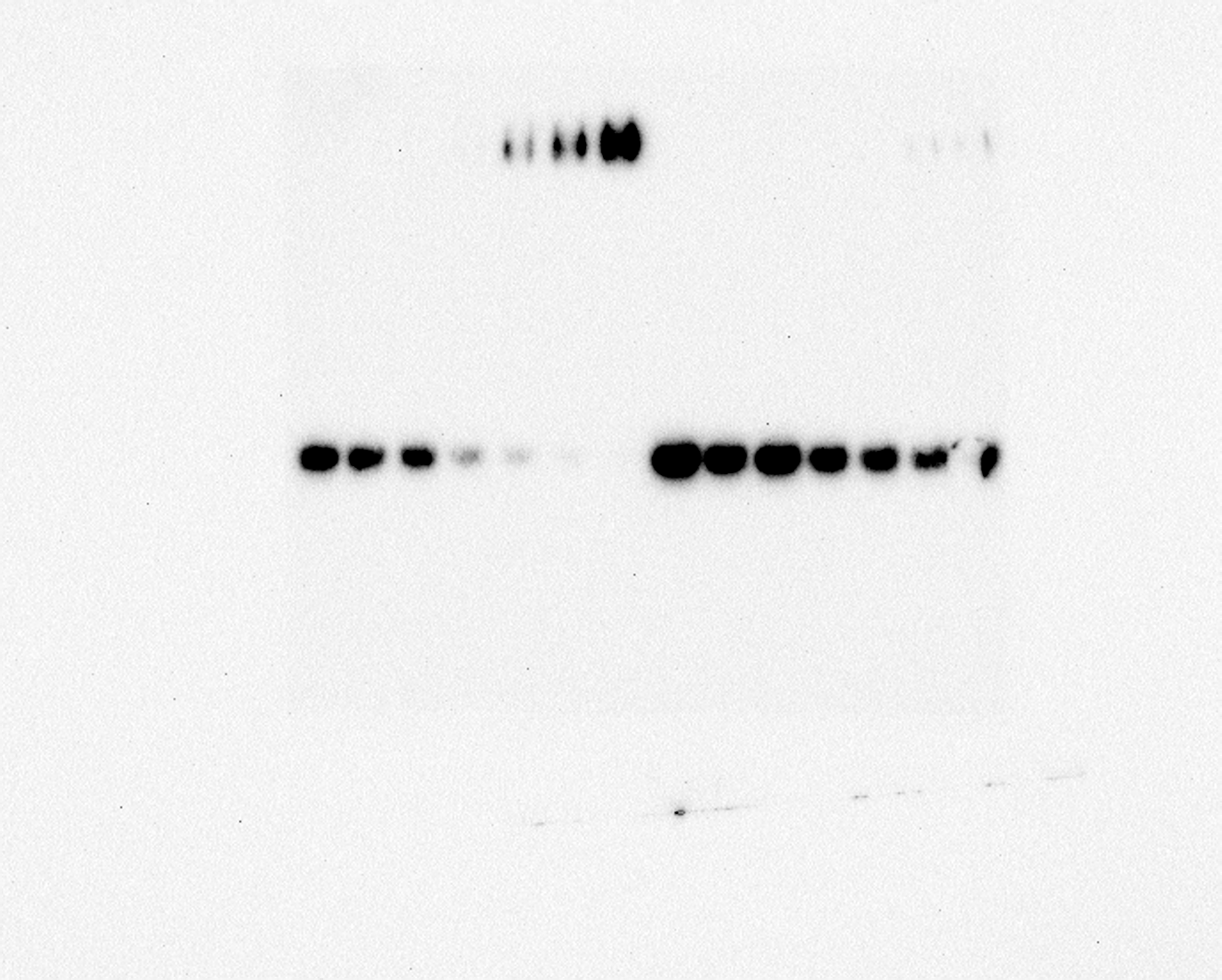

Supplement: Figure 4—source data 1. [file elife-70464-fig4-data1.zip › Figure 4-source data 1/Figure 4C-T0229-18nt- Original.tif]

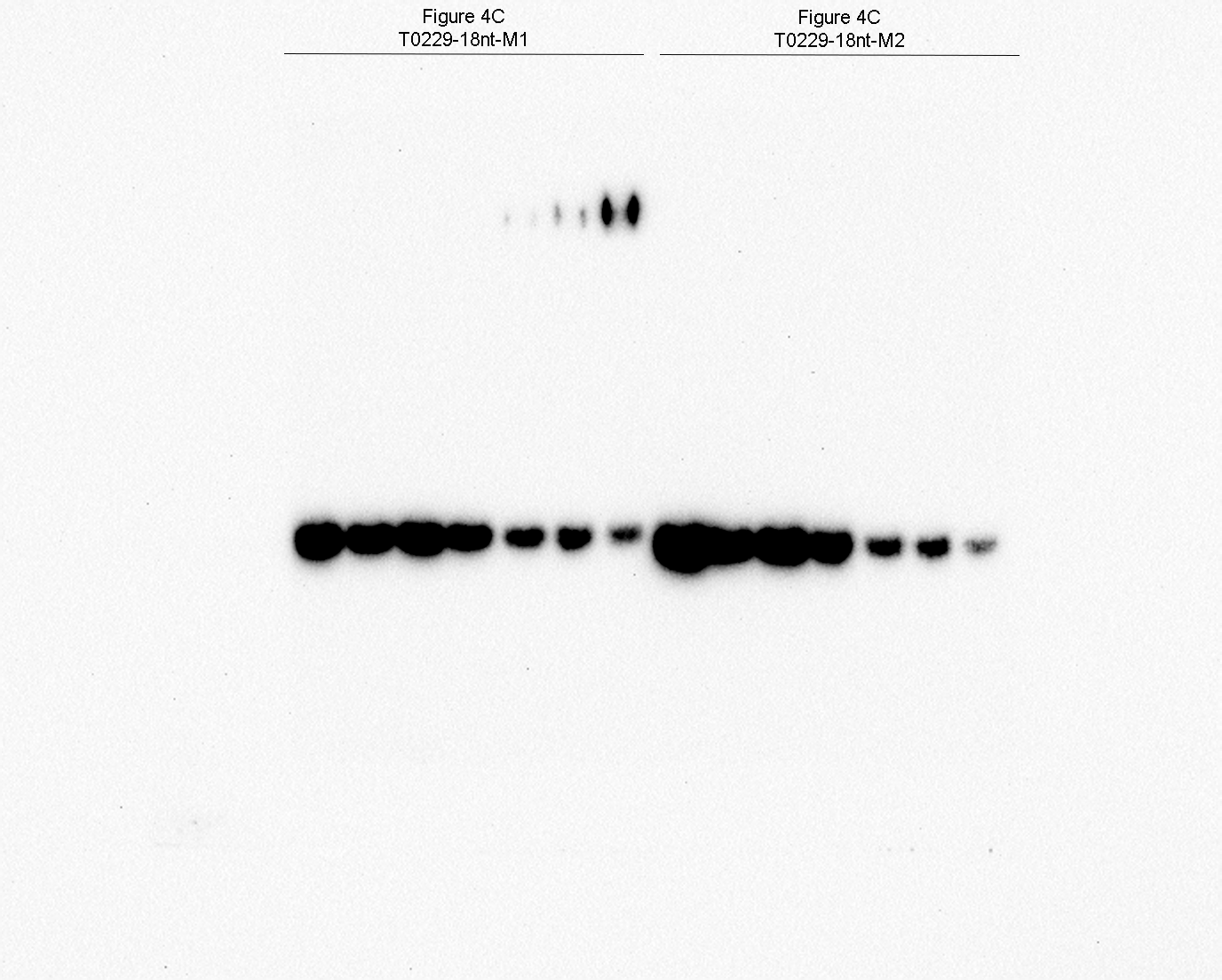

Supplement: Figure 4—source data 1. [file elife-70464-fig4-data1.zip › Figure 4-source data 1/Figure 4C-T0229-18nt-M1 M2-Labeled.tif]

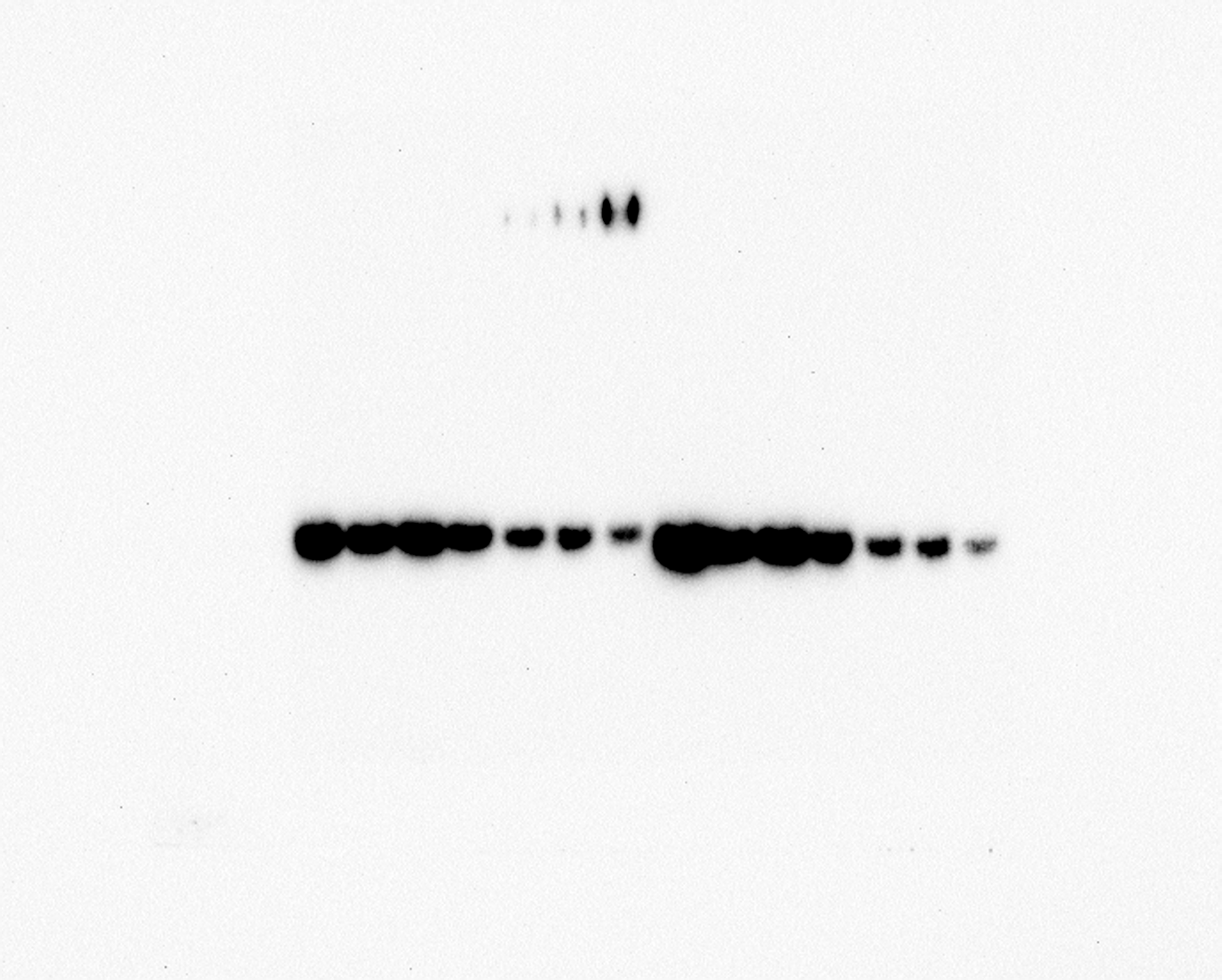

Supplement: Figure 4—source data 1. [file elife-70464-fig4-data1.zip › Figure 4-source data 1/Figure 4C-T0229-18nt-M1 M2-Original.tif]

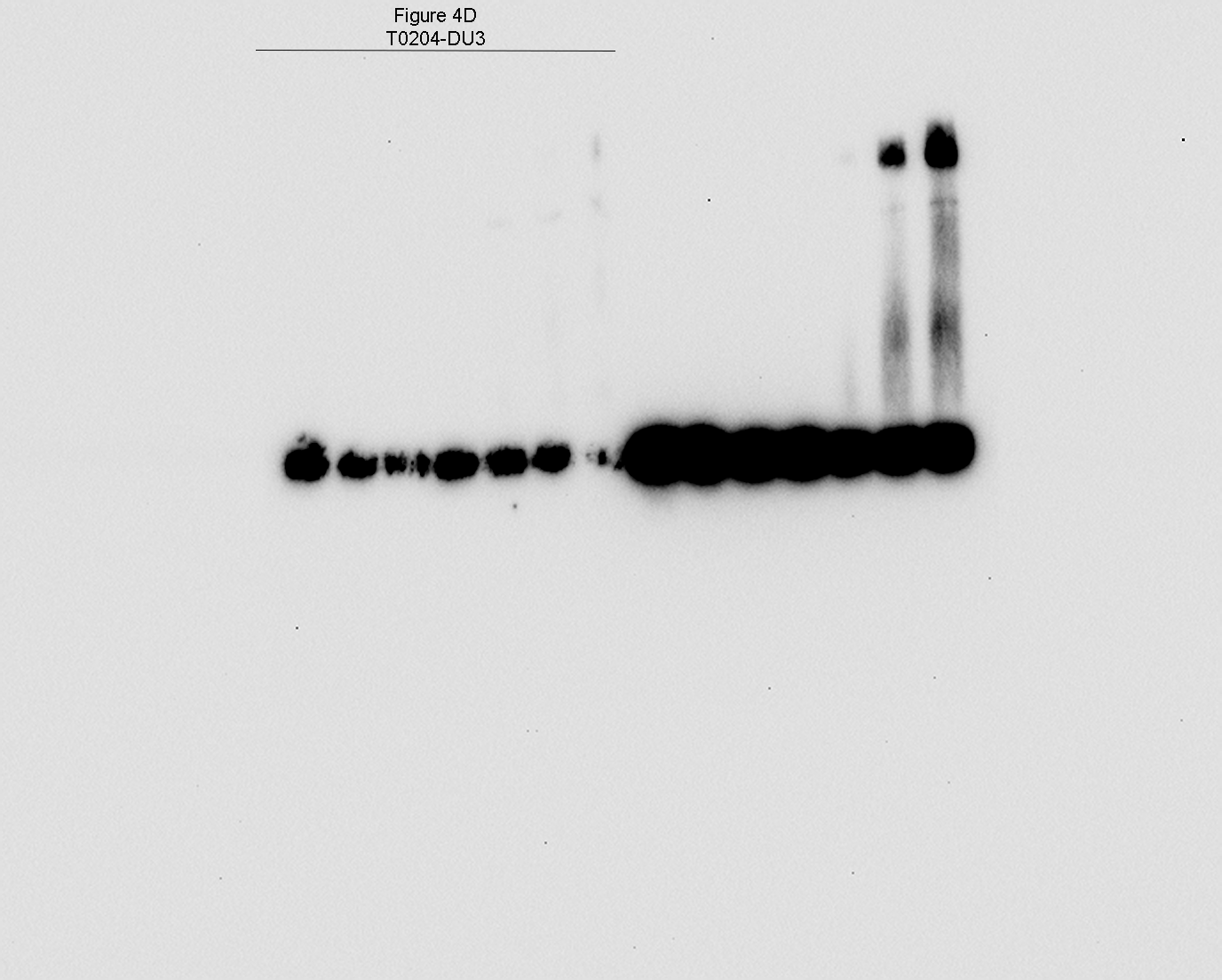

Supplement: Figure 4—source data 1. [file elife-70464-fig4-data1.zip › Figure 4-source data 1/Figure 4D-T0204 DU3-Labeled.tif]

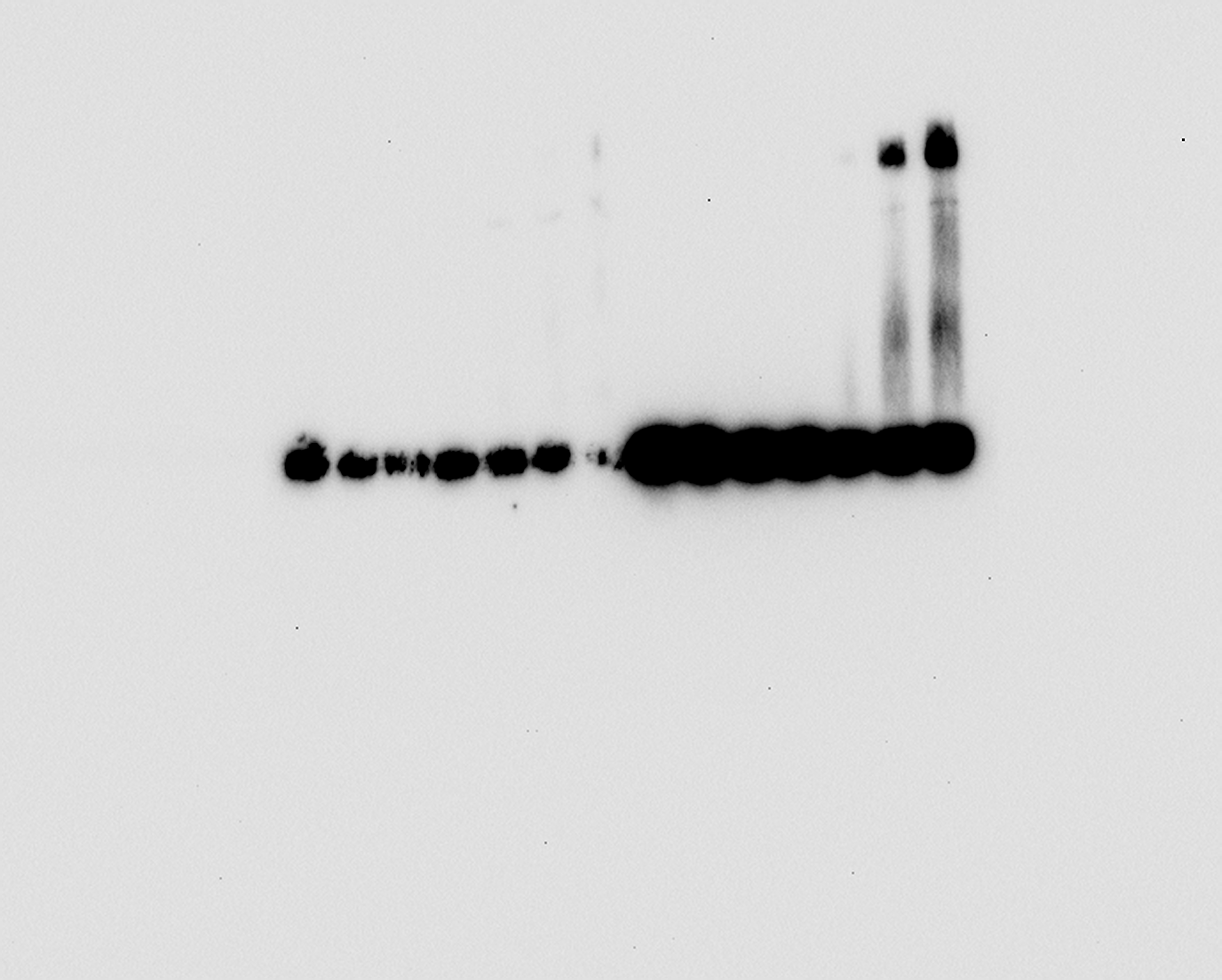

Supplement: Figure 4—source data 1. [file elife-70464-fig4-data1.zip › Figure 4-source data 1/Figure 4D-T0204 DU3-Original.tif]

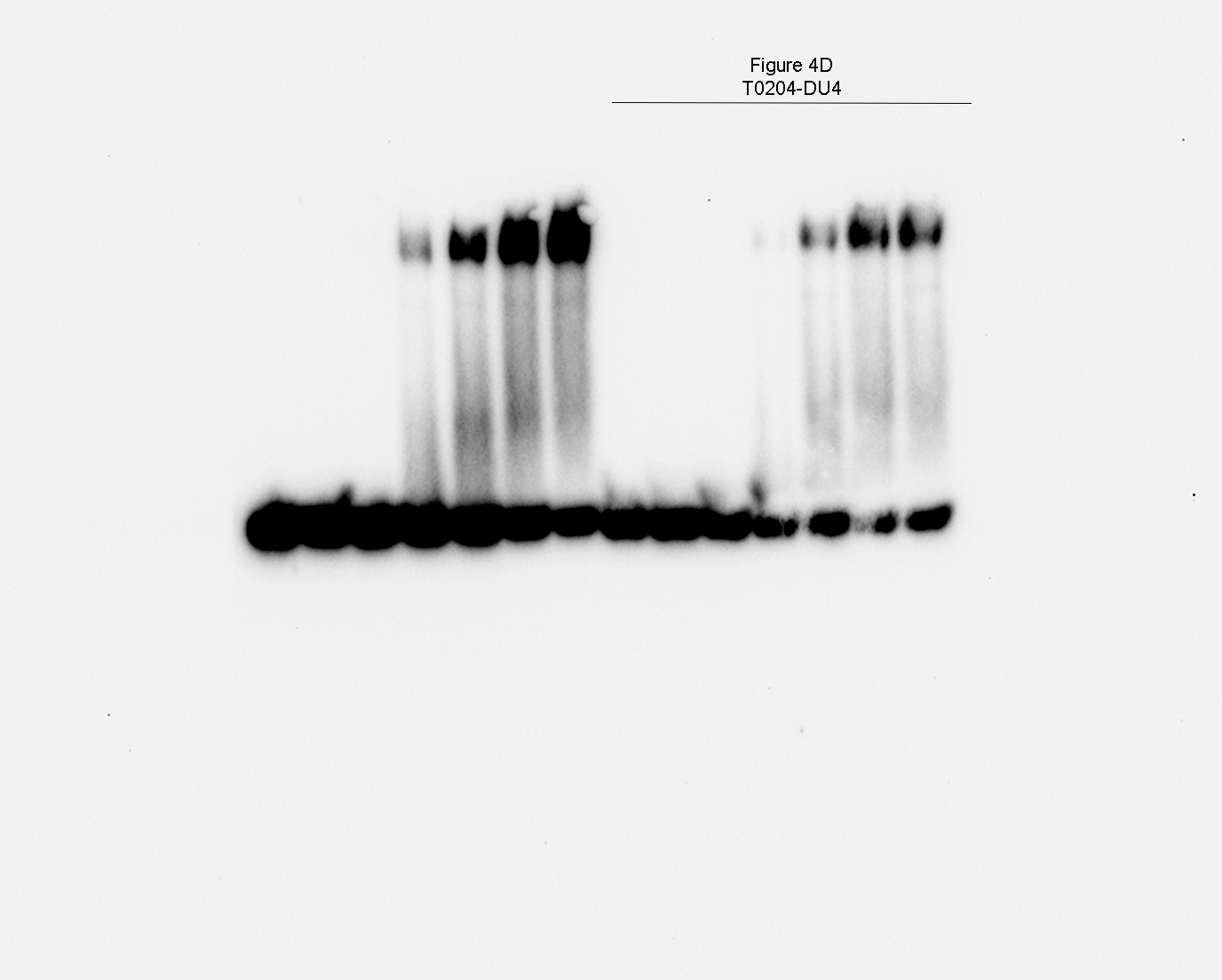

Supplement: Figure 4—source data 1. [file elife-70464-fig4-data1.zip › Figure 4-source data 1/Figure 4D-T0204 DU4-Labeled.tif]

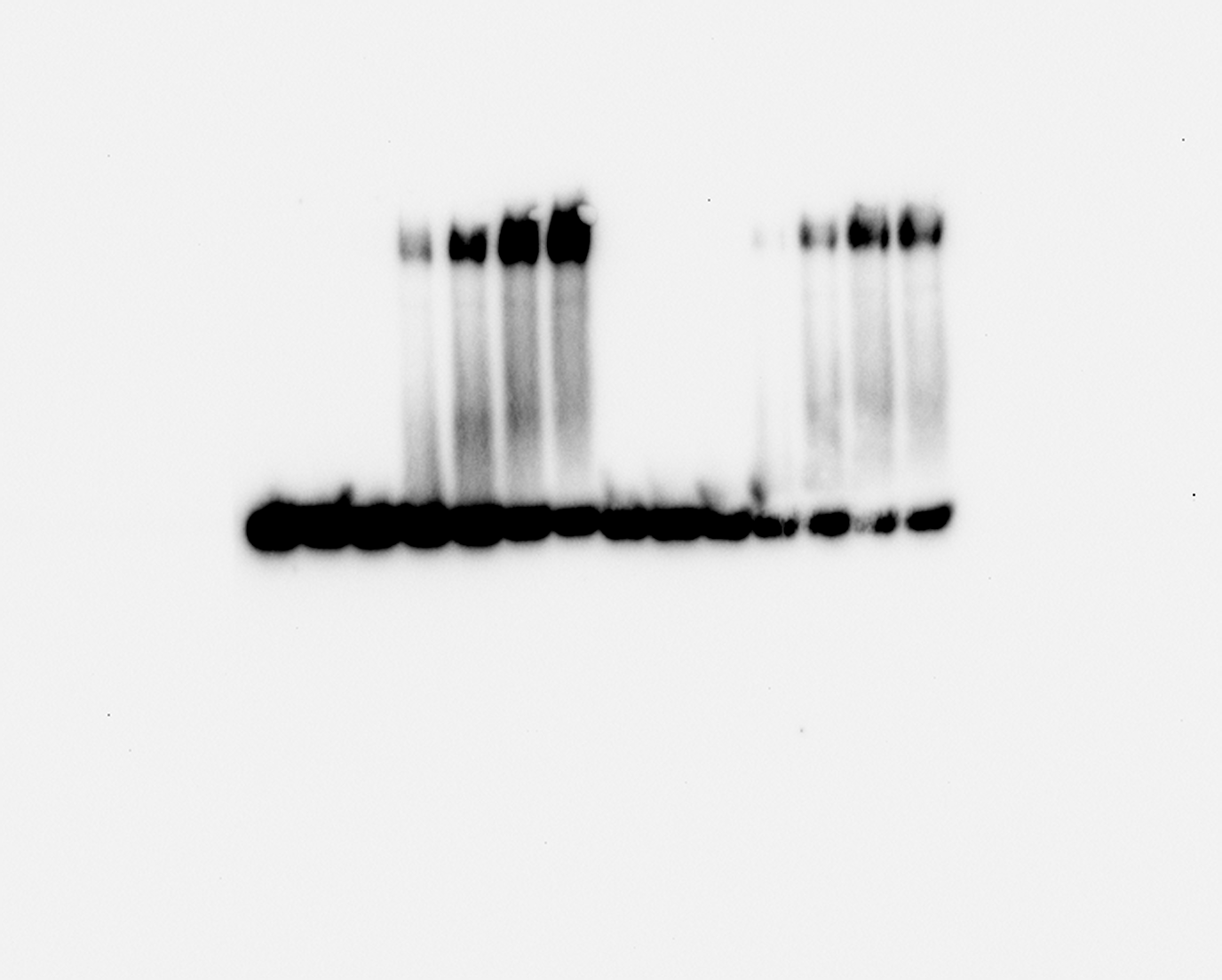

Supplement: Figure 4—source data 1. [file elife-70464-fig4-data1.zip › Figure 4-source data 1/Figure 4D-T0204 DU4-Original.tif]

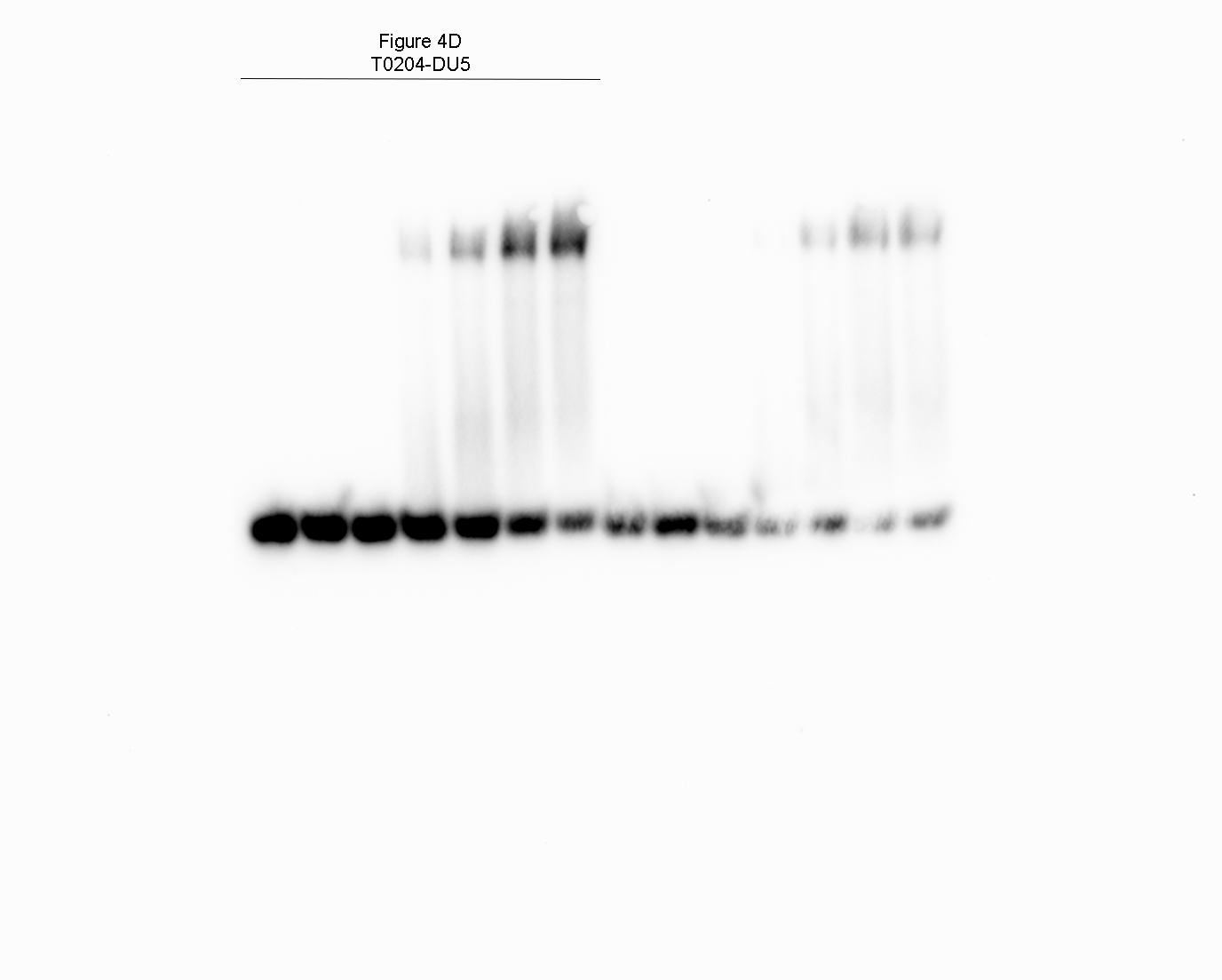

Supplement: Figure 4—source data 1. [file elife-70464-fig4-data1.zip › Figure 4-source data 1/Figure 4D-T0204 DU5-Labeled.tif]

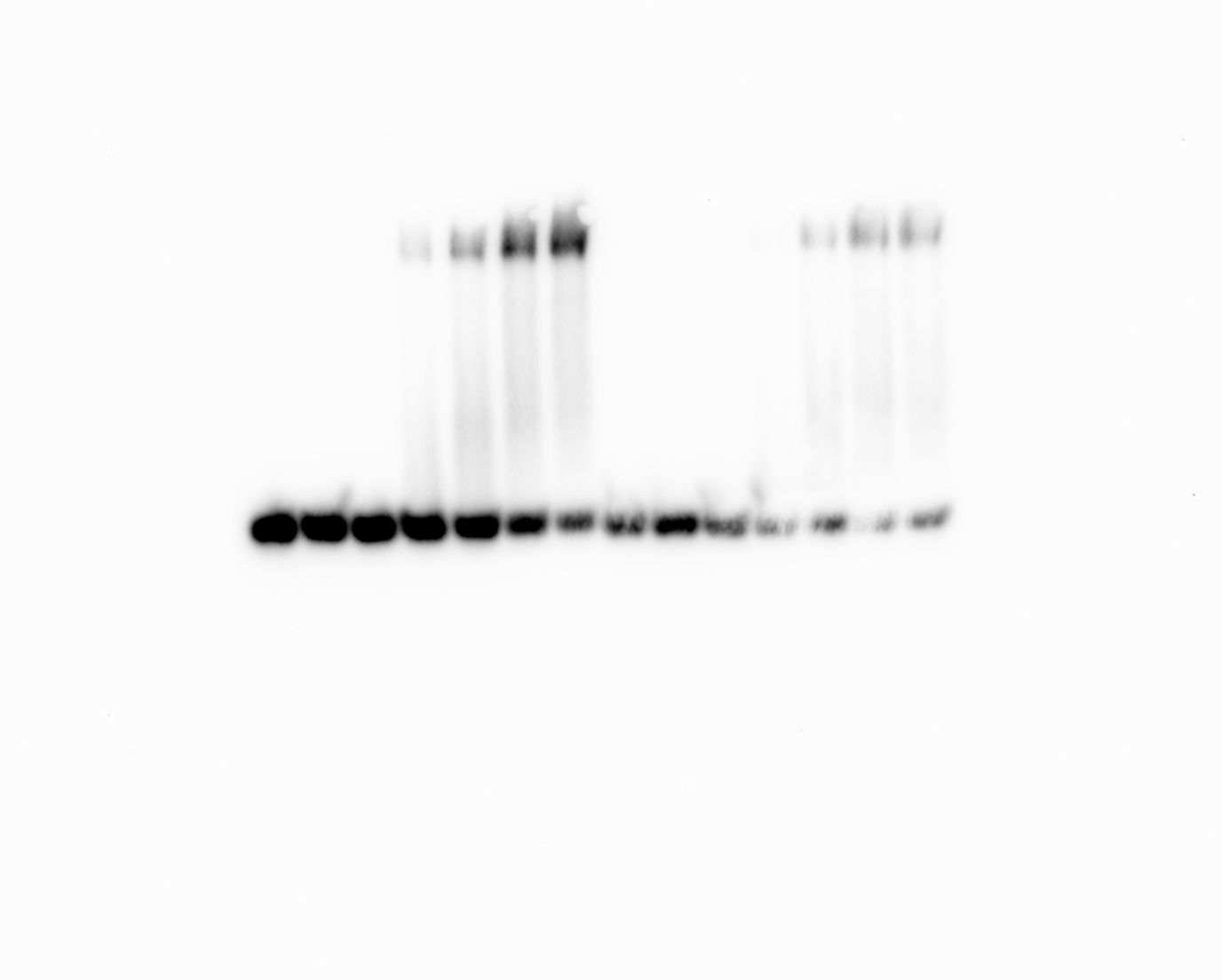

Supplement: Figure 4—source data 1. [file elife-70464-fig4-data1.zip › Figure 4-source data 1/Figure 4D-T0204 DU5-Original.tif]

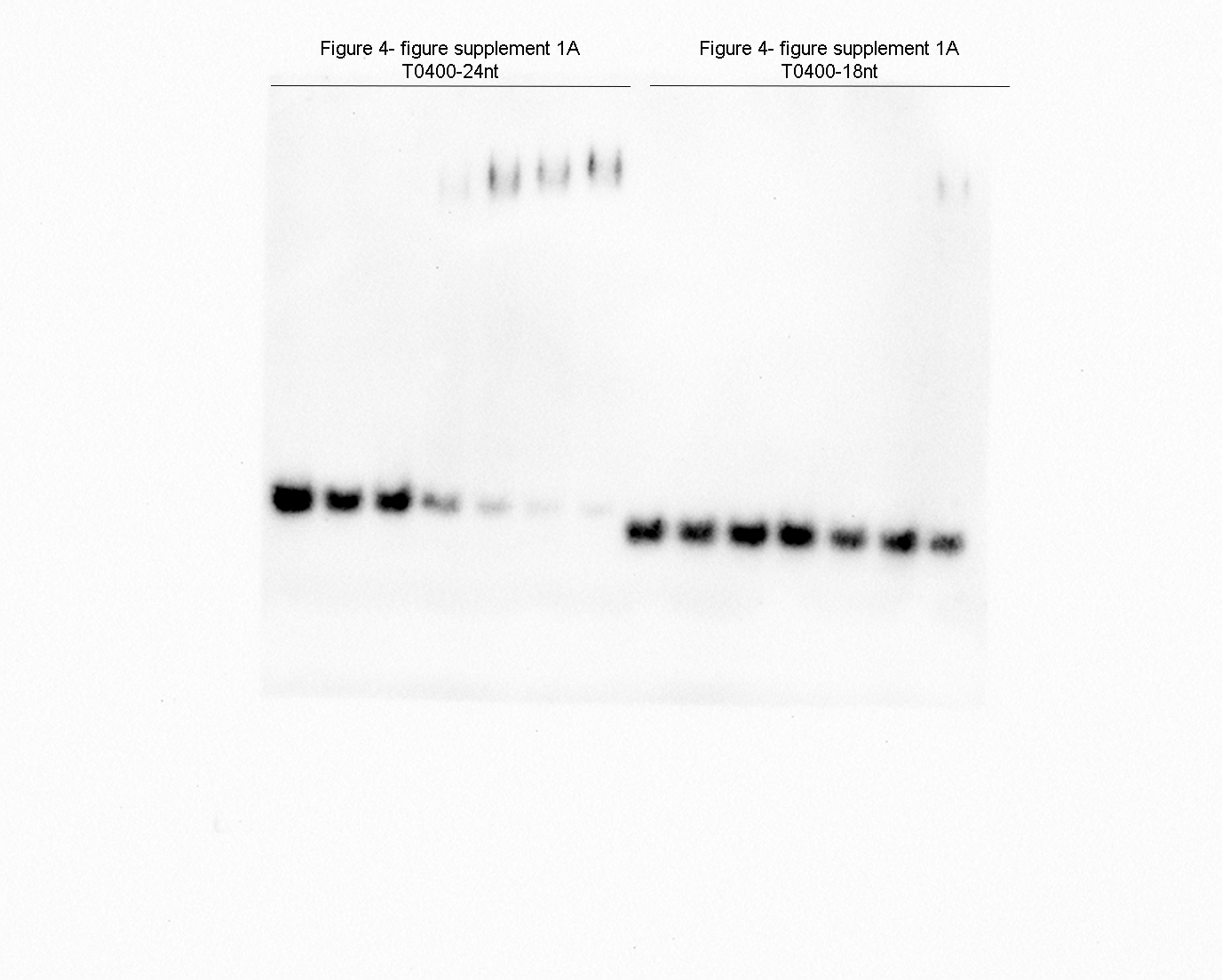

Supplement: Figure 4—figure supplement 1—source data 1. [file elife-70464-fig4-figsupp1-data1.zip › Figure 4-figure supplement 1-source data 1/Figure 4-figure supplement 1A-T0400-24nt 18nt-Labeled.tif]

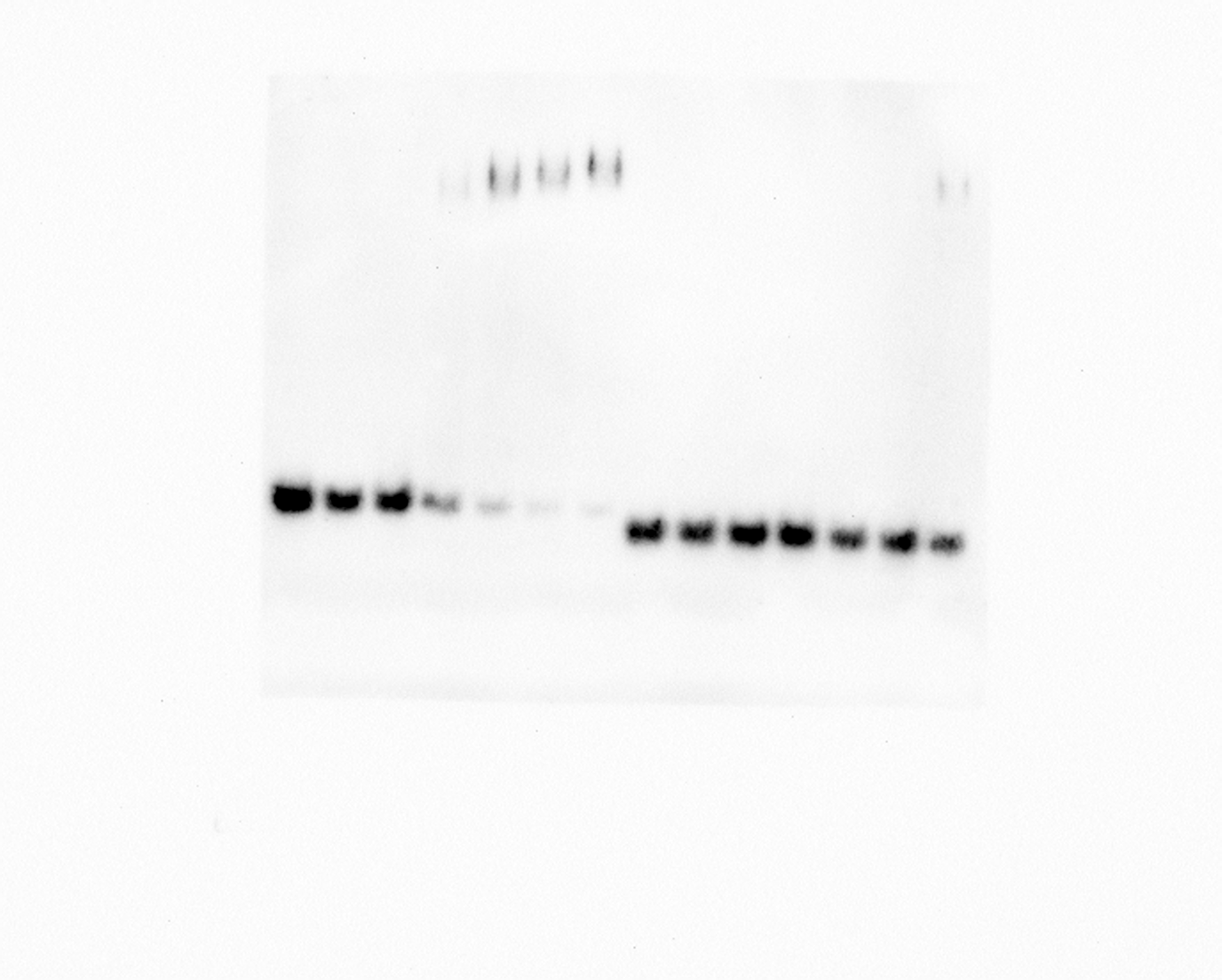

Supplement: Figure 4—figure supplement 1—source data 1. [file elife-70464-fig4-figsupp1-data1.zip › Figure 4-figure supplement 1-source data 1/Figure 4-figure supplement 1A-T0400-24nt 18nt-Original.tif]

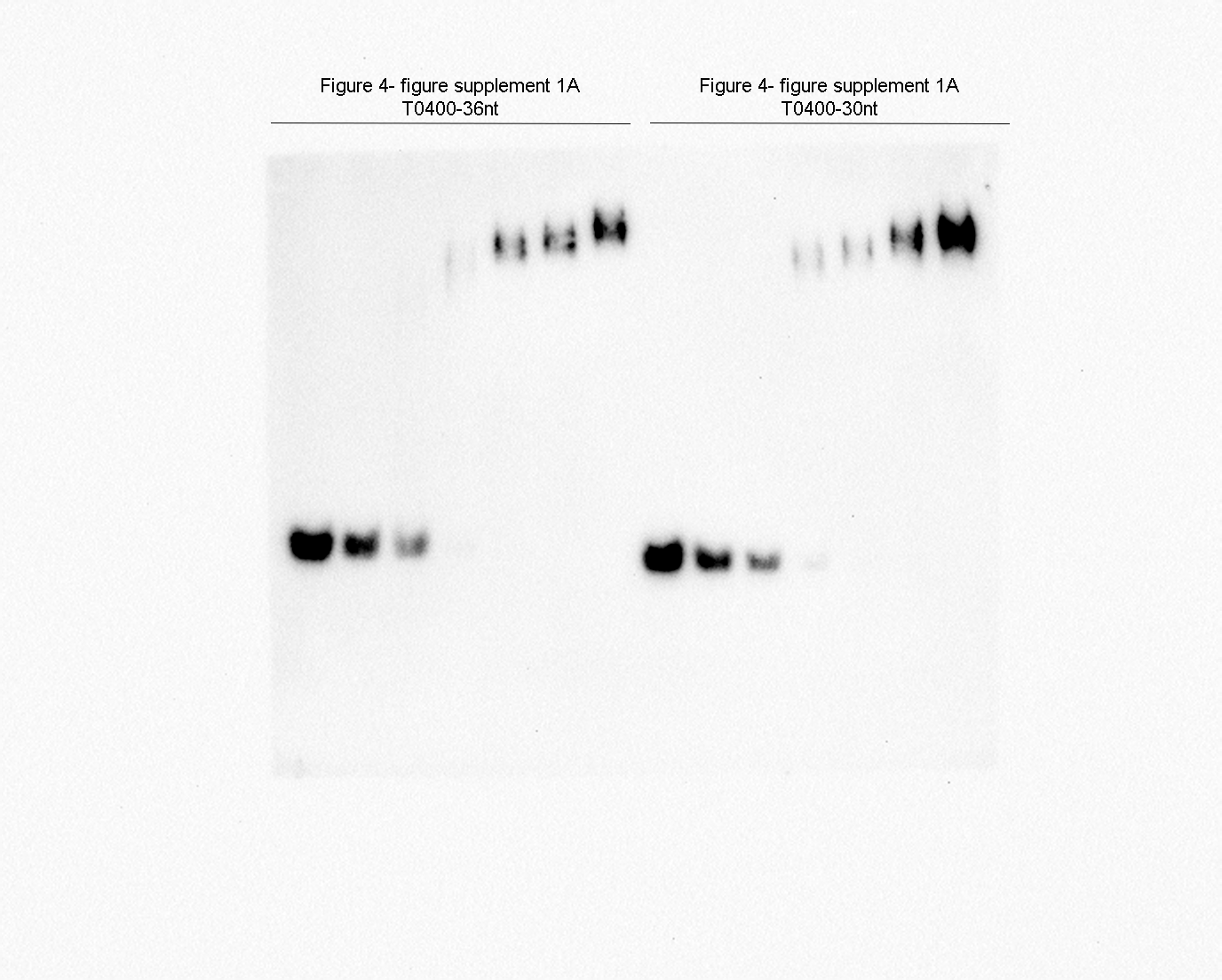

Supplement: Figure 4—figure supplement 1—source data 1. [file elife-70464-fig4-figsupp1-data1.zip › Figure 4-figure supplement 1-source data 1/Figure 4-figure supplement 1A-T0400-36nt 30nt-Labeled.tif]

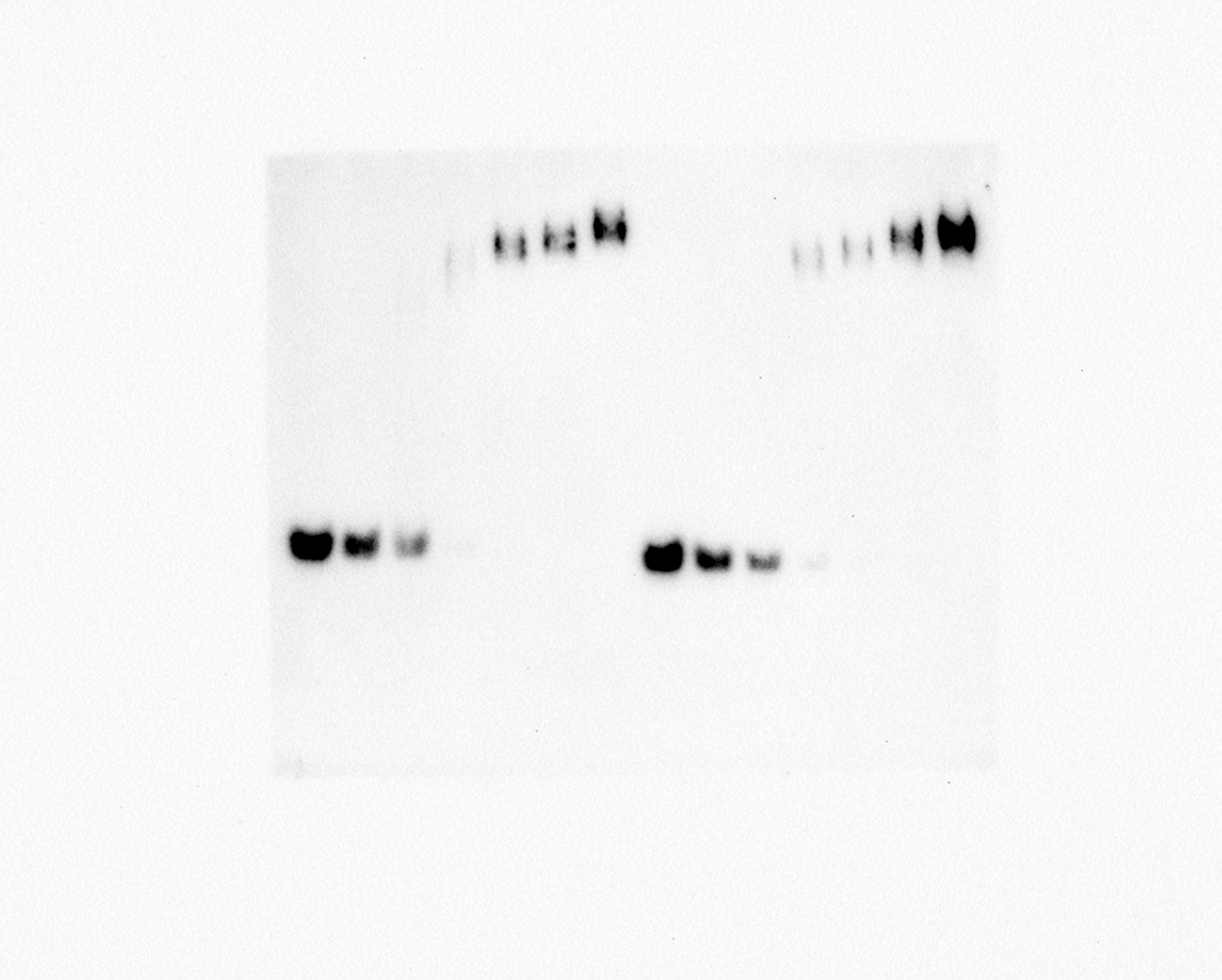

Supplement: Figure 4—figure supplement 1—source data 1. [file elife-70464-fig4-figsupp1-data1.zip › Figure 4-figure supplement 1-source data 1/Figure 4-figure supplement 1A-T0400-36nt 30nt-Original.tif]

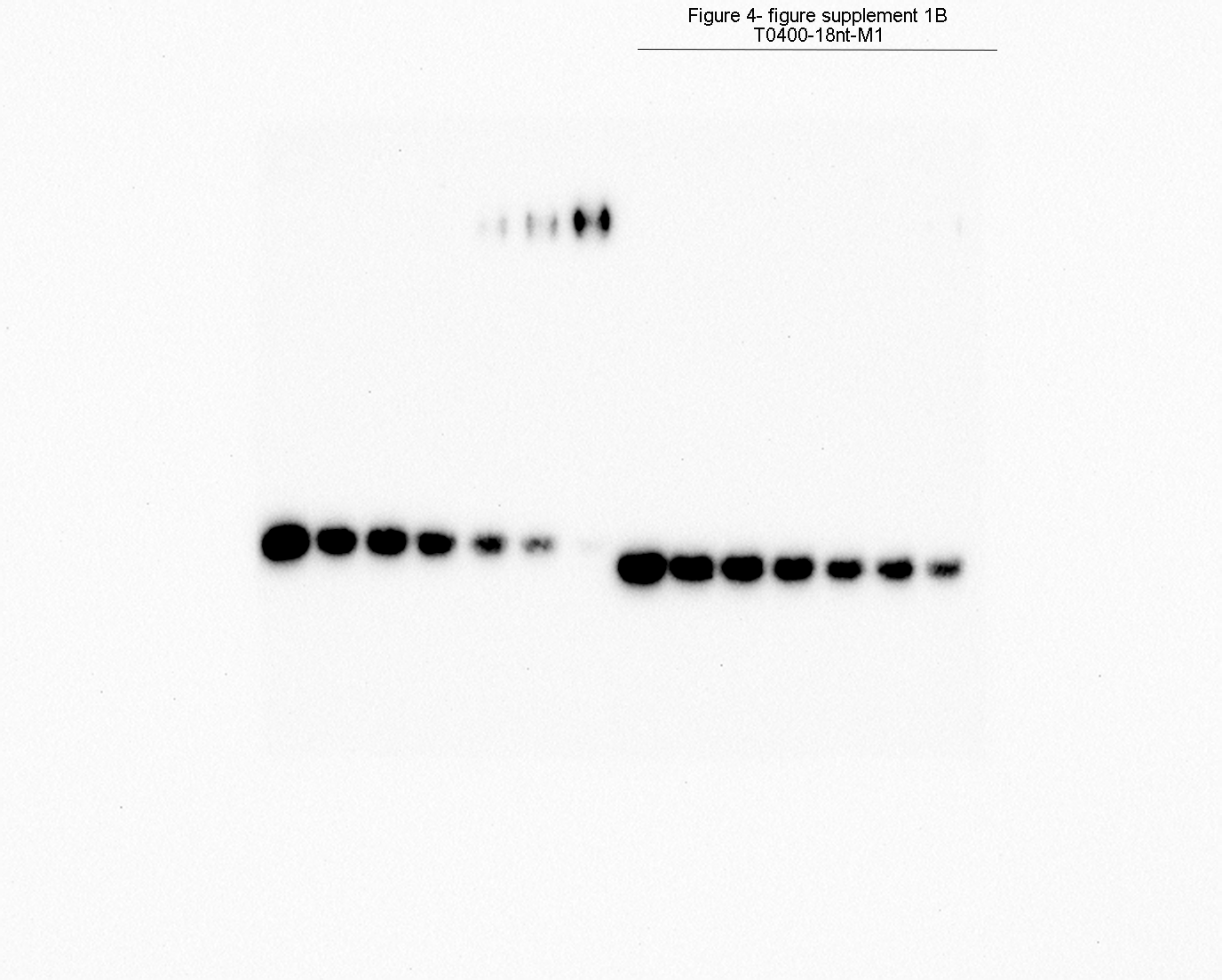

Supplement: Figure 4—figure supplement 1—source data 1. [file elife-70464-fig4-figsupp1-data1.zip › Figure 4-figure supplement 1-source data 1/Figure 4-figure supplement 1B-T0400-18nt M1-Labeled.tif]

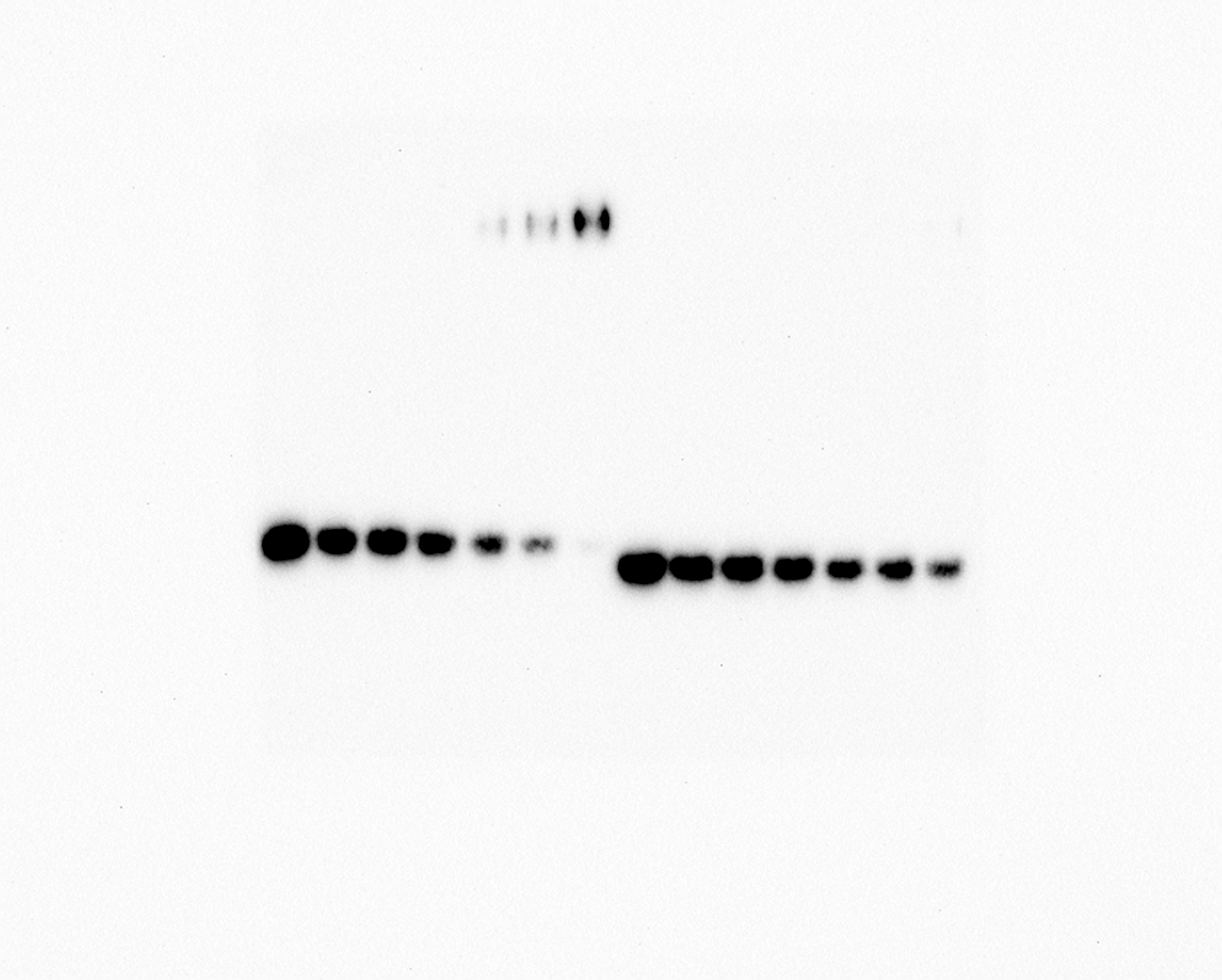

Supplement: Figure 4—figure supplement 1—source data 1. [file elife-70464-fig4-figsupp1-data1.zip › Figure 4-figure supplement 1-source data 1/Figure 4-figure supplement 1B-T0400-18nt M1-Original.tif]

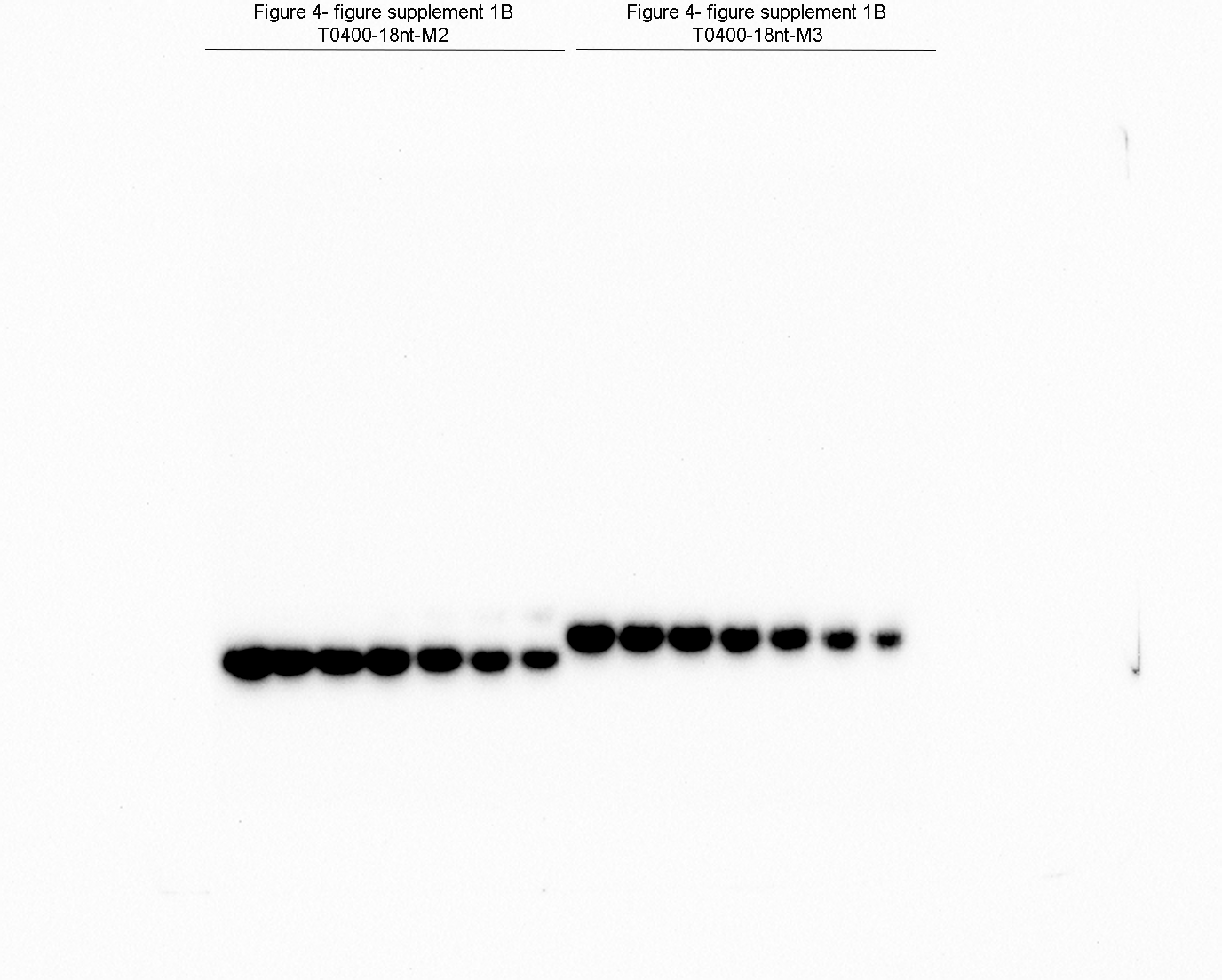

Supplement: Figure 4—figure supplement 1—source data 1. [file elife-70464-fig4-figsupp1-data1.zip › Figure 4-figure supplement 1-source data 1/Figure 4-figure supplement 1B-T0400-18nt M2 M3-Labeled.tif]

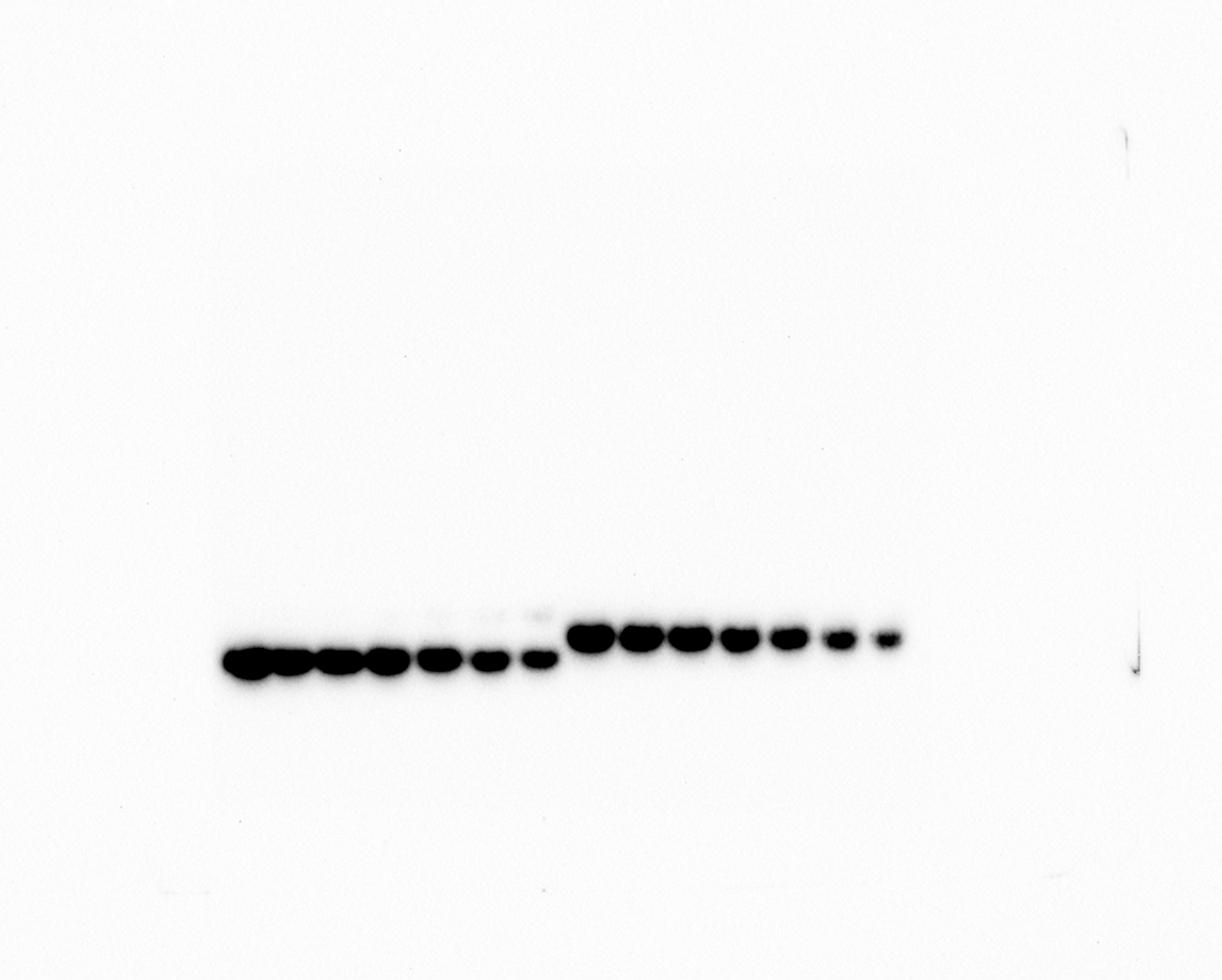

Supplement: Figure 4—figure supplement 1—source data 1. [file elife-70464-fig4-figsupp1-data1.zip › Figure 4-figure supplement 1-source data 1/Figure 4-figure supplement 1B-T0400-18nt M2 M3-Original.tif]

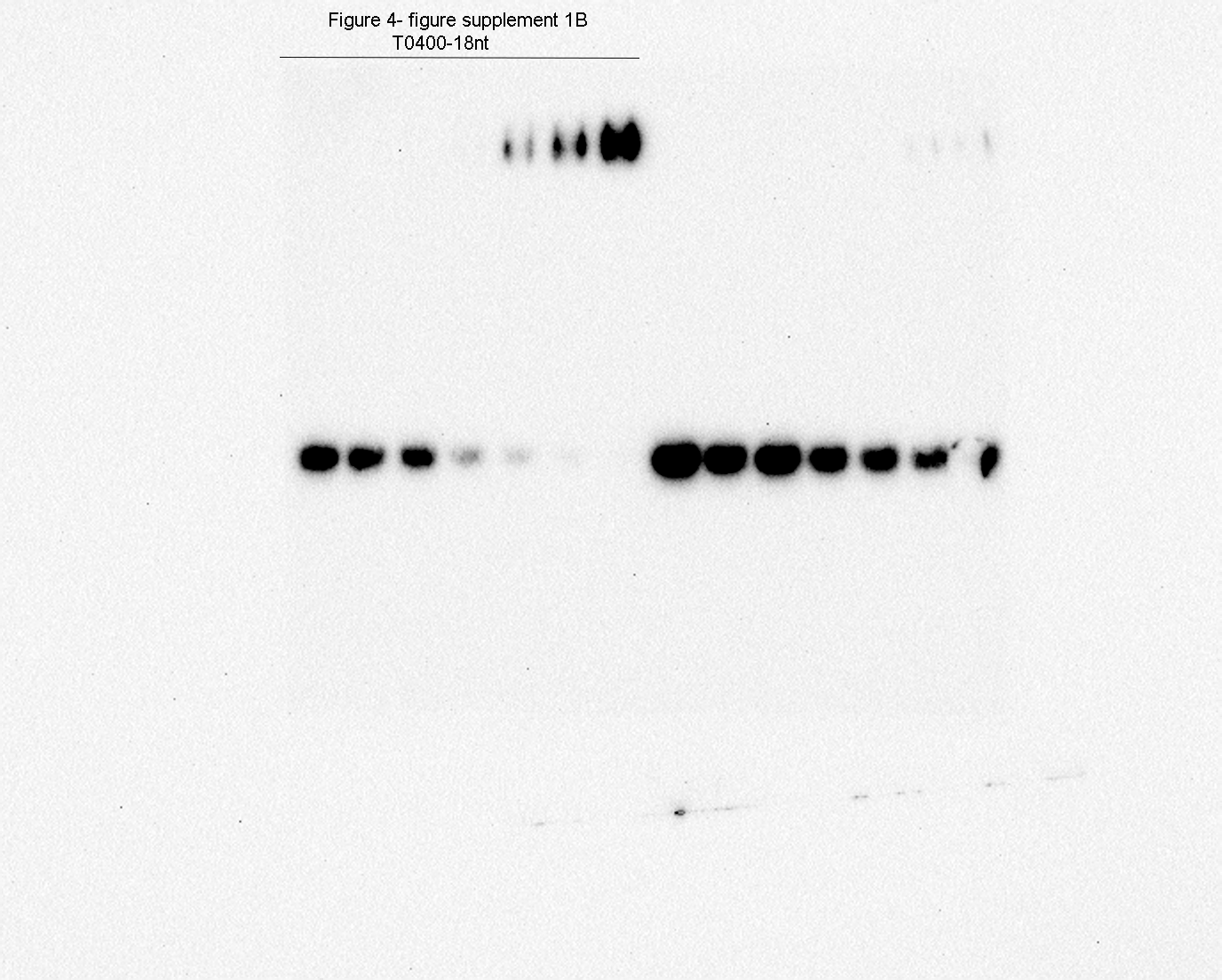

Supplement: Figure 4—figure supplement 1—source data 1. [file elife-70464-fig4-figsupp1-data1.zip › Figure 4-figure supplement 1-source data 1/Figure 4-figure supplement 1B-T0400-18nt-Labeled.tif]

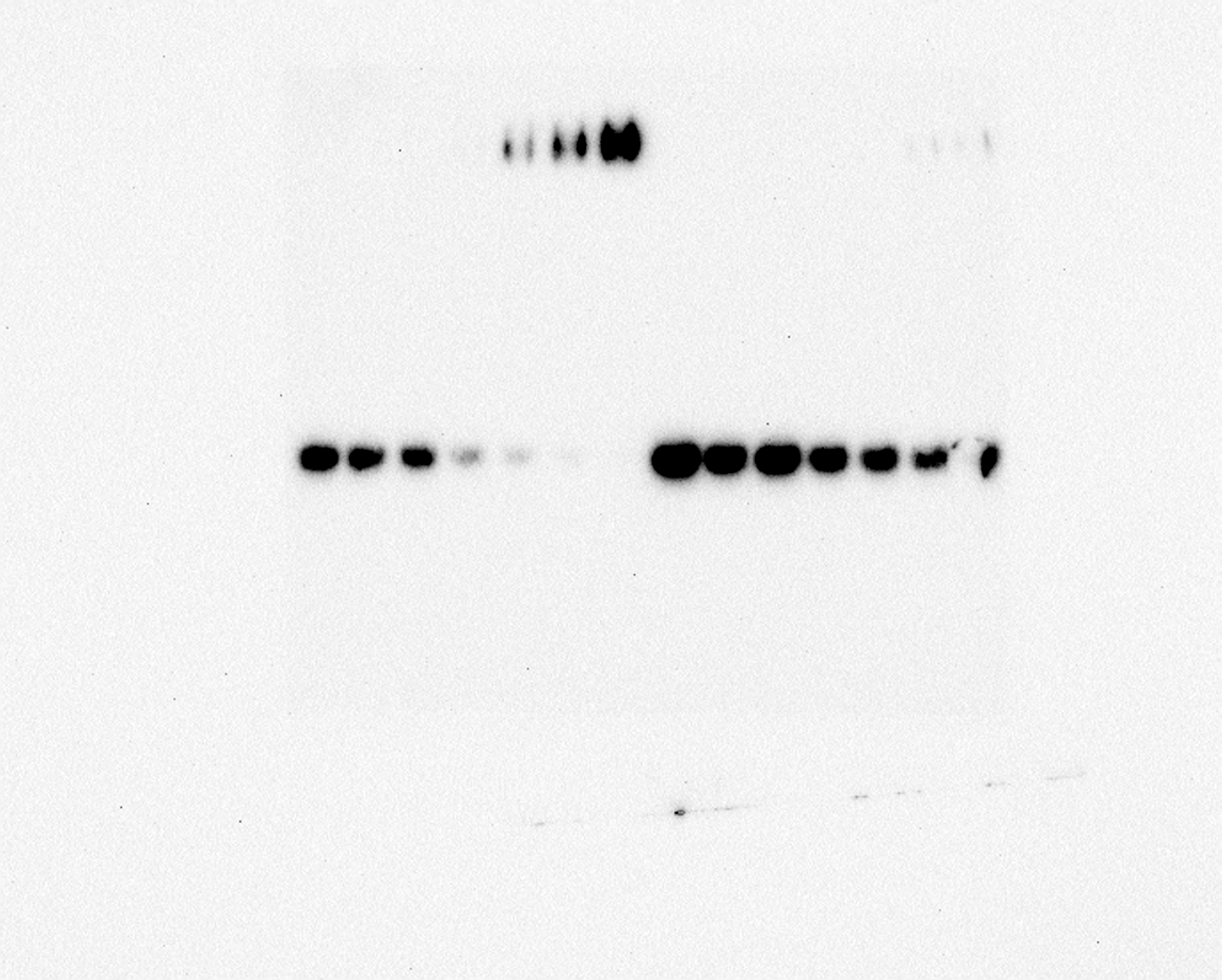

Supplement: Figure 4—figure supplement 1—source data 1. [file elife-70464-fig4-figsupp1-data1.zip › Figure 4-figure supplement 1-source data 1/Figure 4-figure supplement 1B-T0400-18nt-Original.tif]

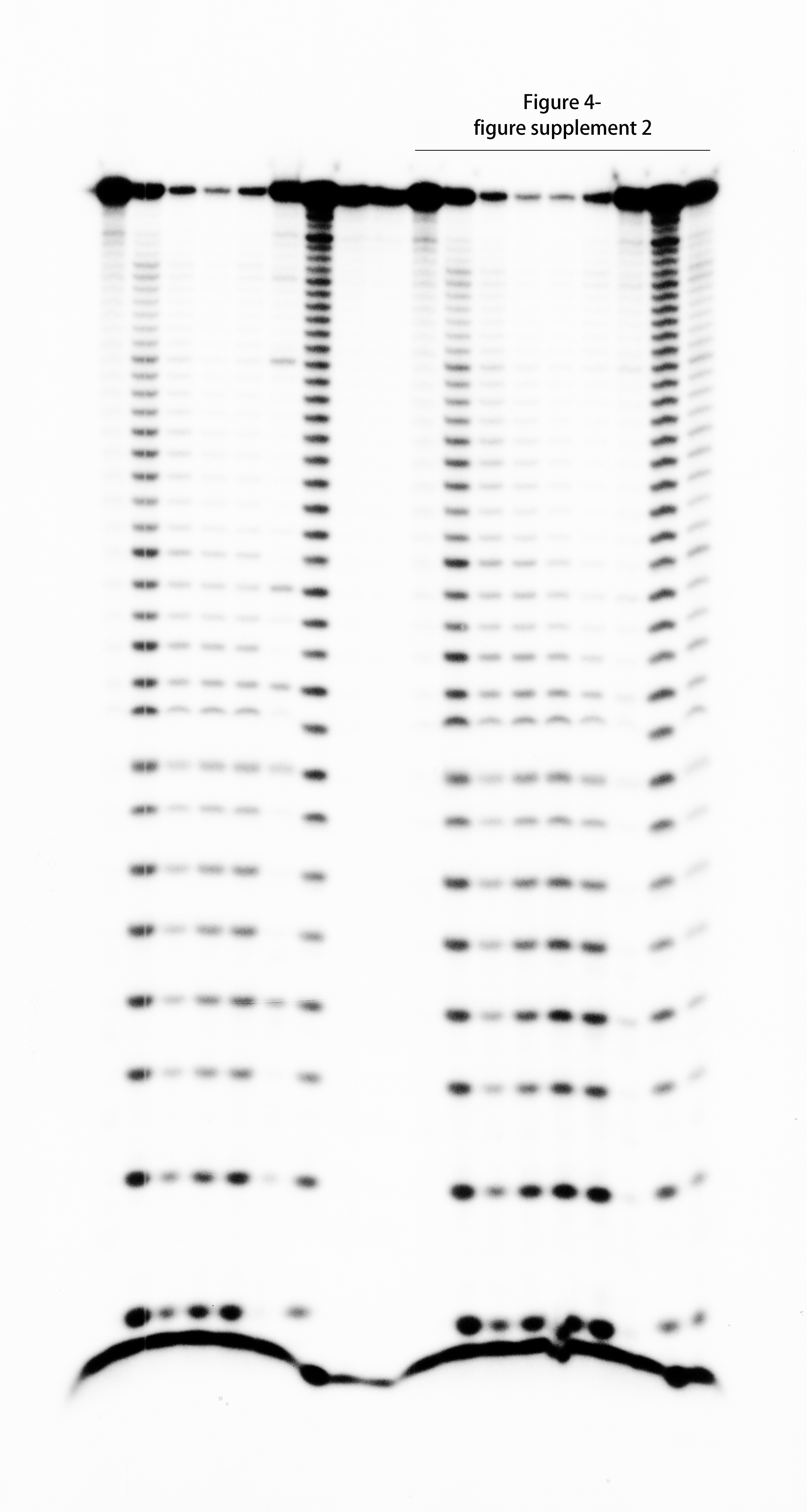

Supplement: Figure 4—figure supplement 3—source data 1. [file elife-70464-fig4-figsupp3-data1.zip › Figure 4-figure supplement 2-source data 1/Figure 4-figure supplement 2-Labeled.tif]

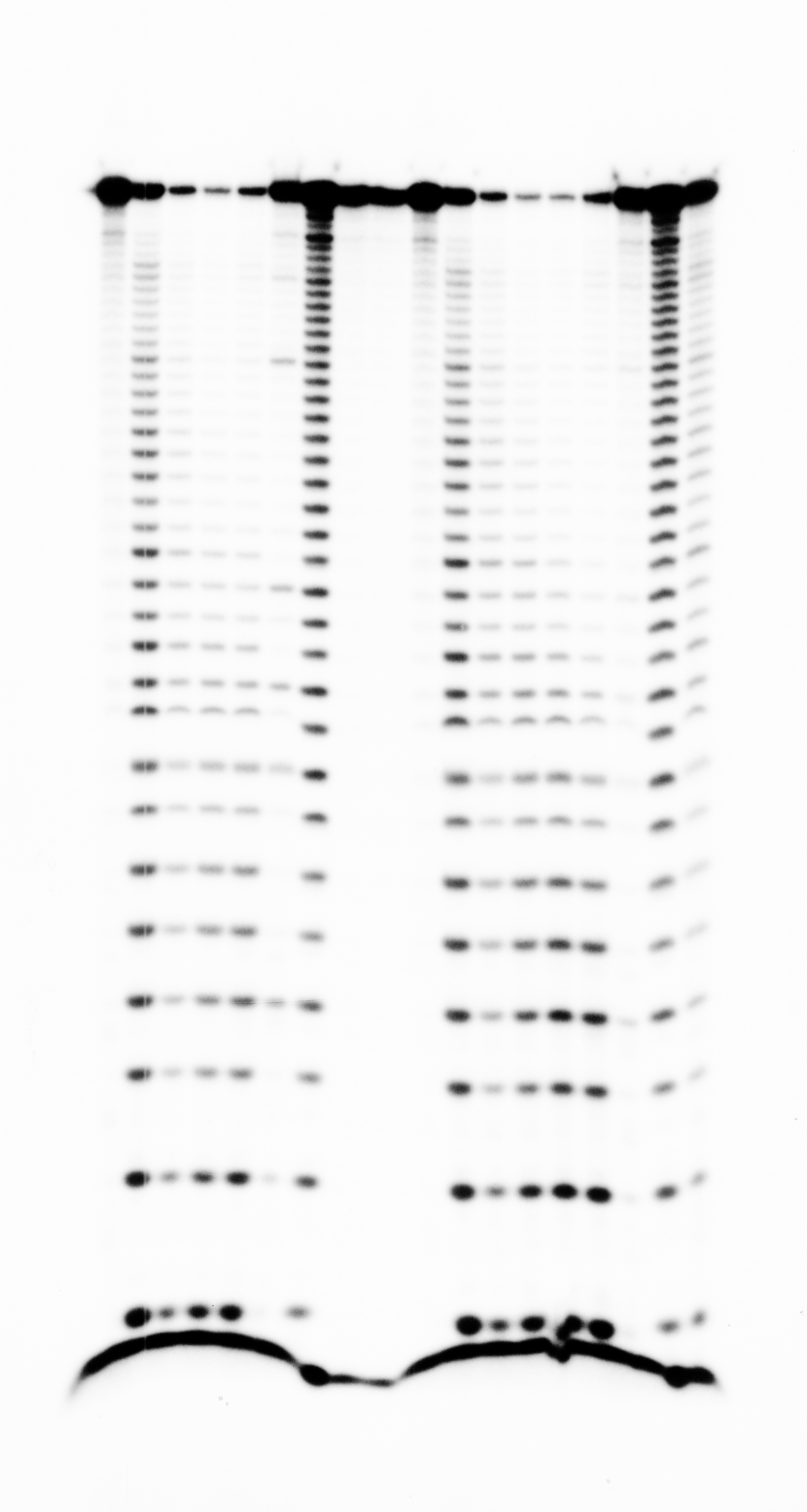

Supplement: Figure 4—figure supplement 3—source data 1. [file elife-70464-fig4-figsupp3-data1.zip › Figure 4-figure supplement 2-source data 1/Figure 4-figure supplement 2-Original.tif]

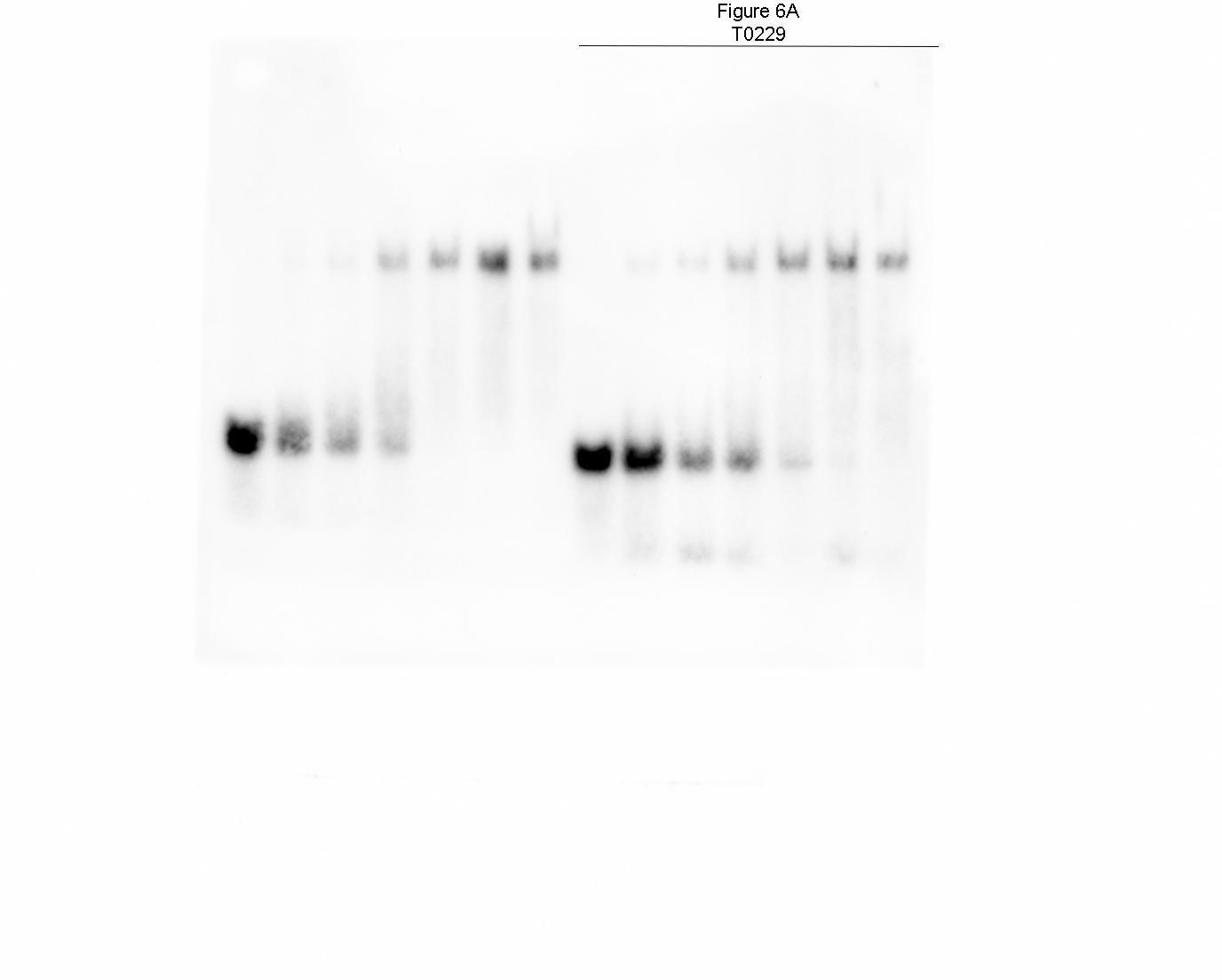

Supplement: Figure 6—source data 1. [file elife-70464-fig6-data1.zip › Figure 6-source data 1/Figure 6A-T0229-Labeled.tif]

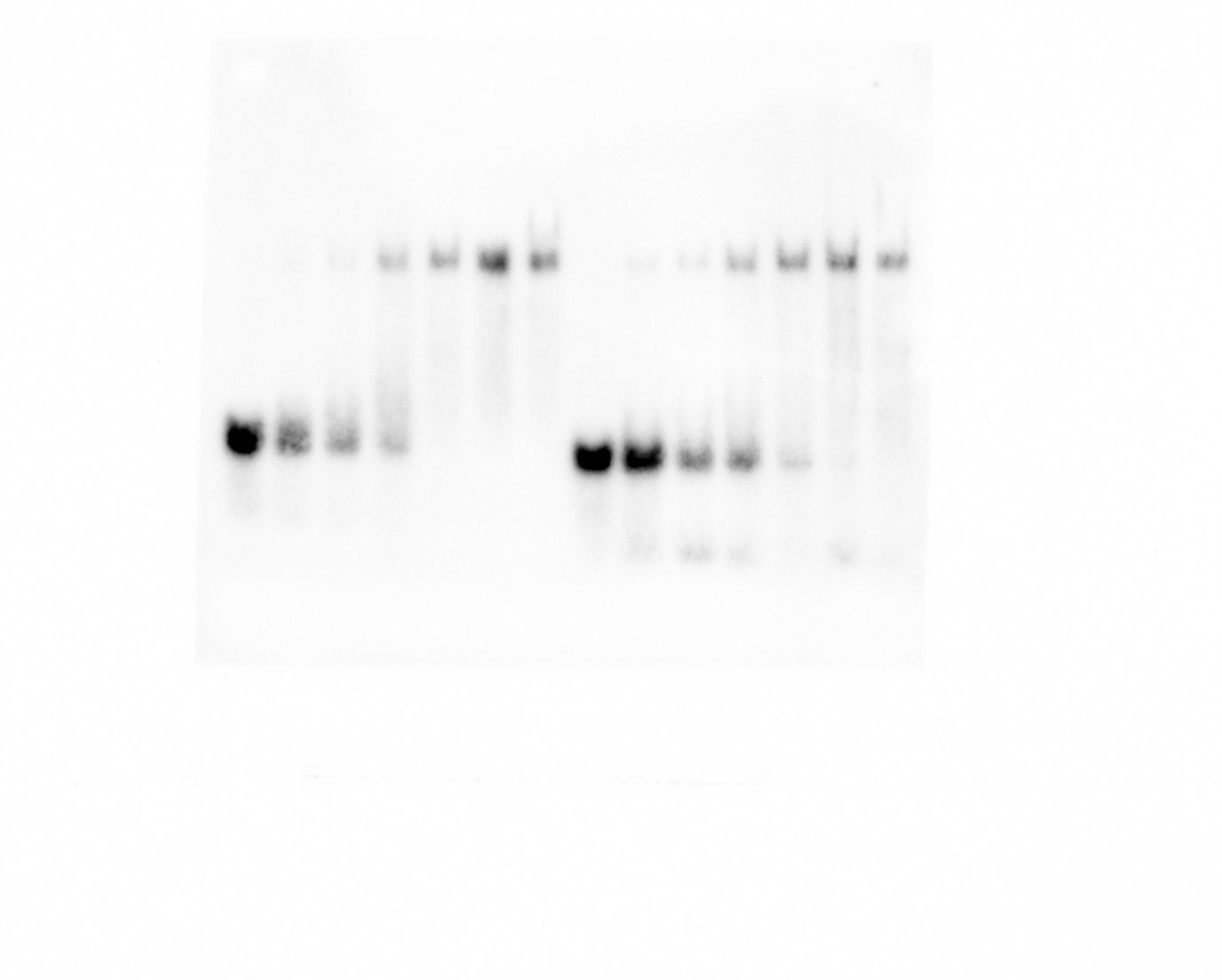

Supplement: Figure 6—source data 1. [file elife-70464-fig6-data1.zip › Figure 6-source data 1/Figure 6A-T0229-Original.tif]

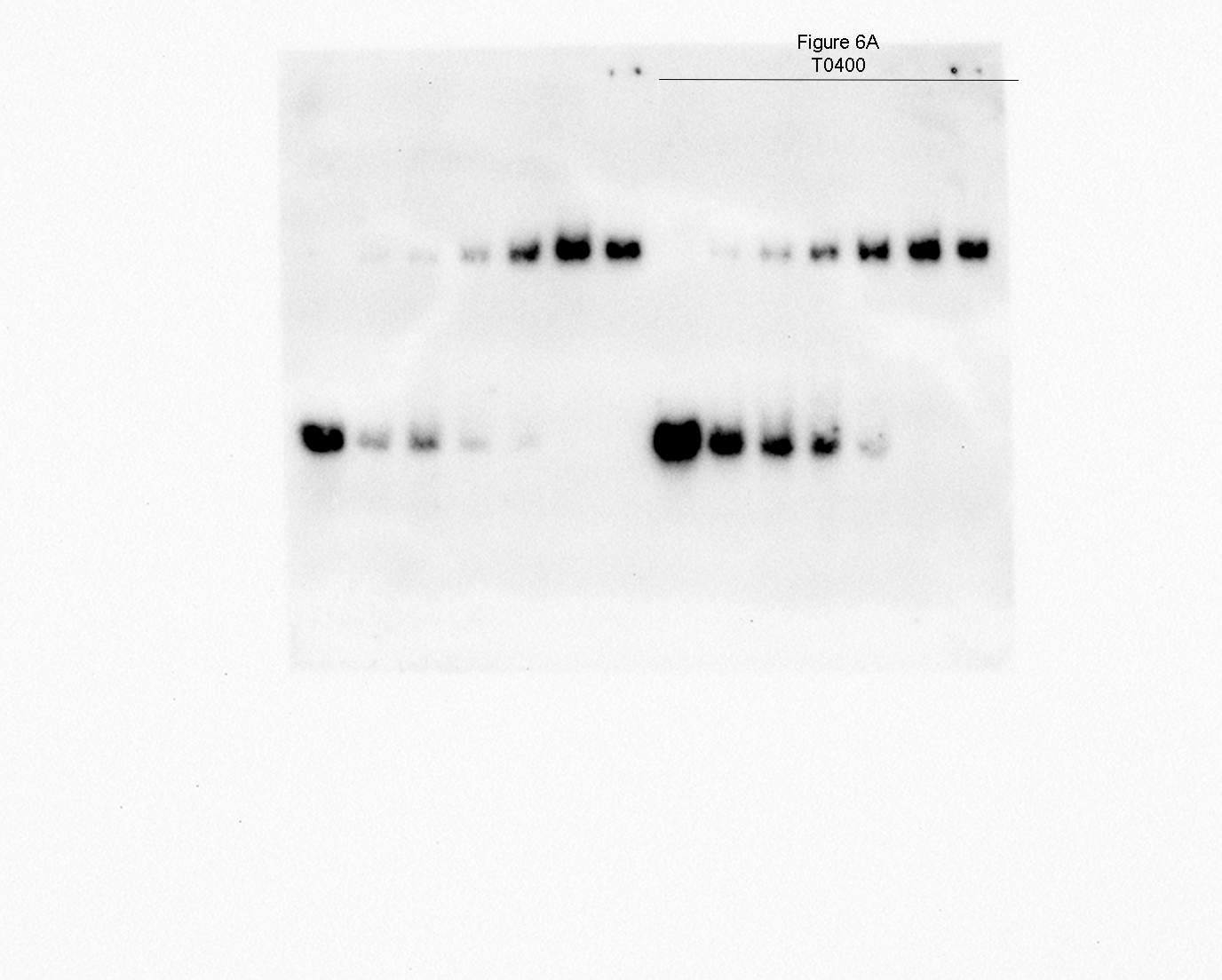

Supplement: Figure 6—source data 1. [file elife-70464-fig6-data1.zip › Figure 6-source data 1/Figure 6A-T0400-Labeled.tif]

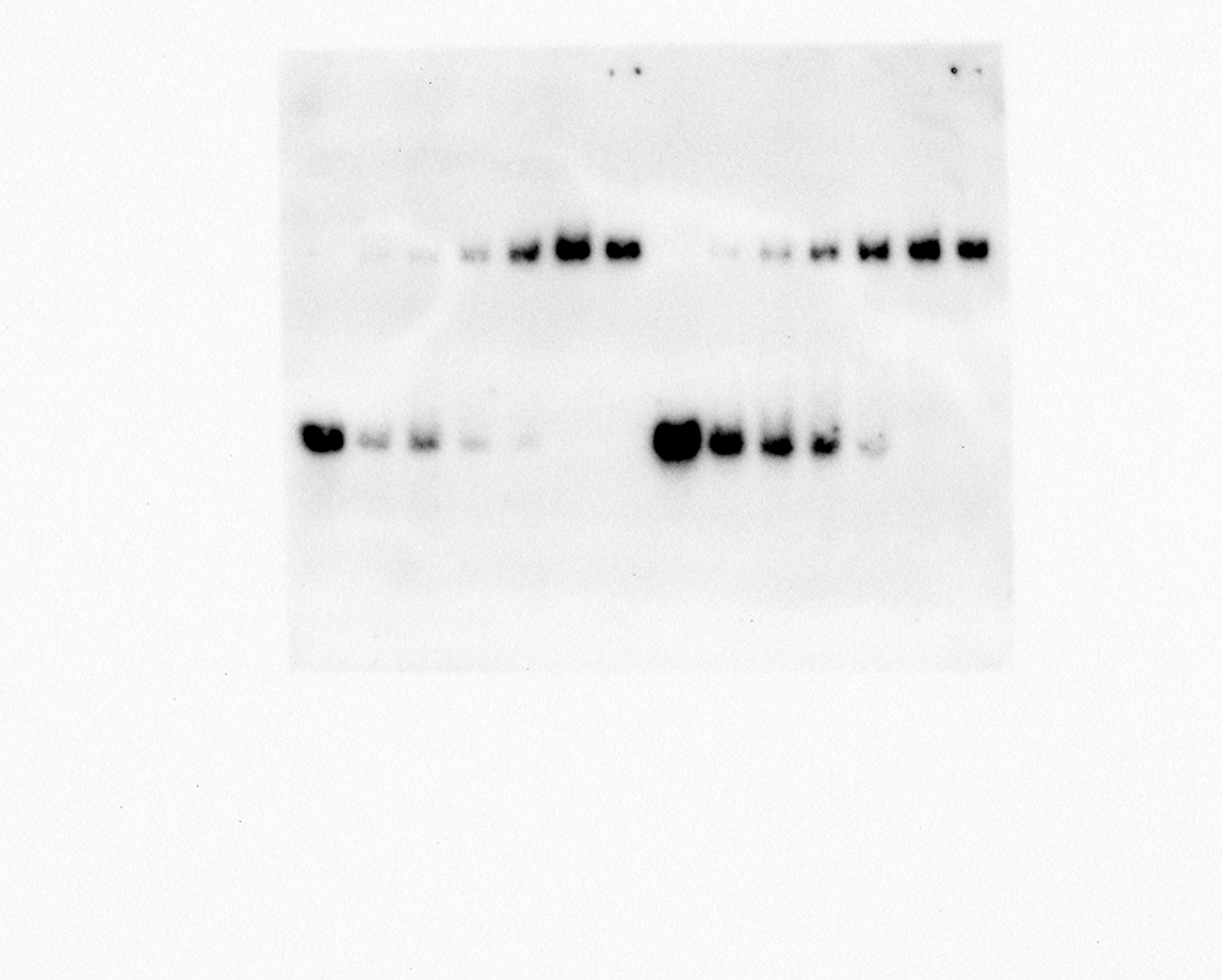

Supplement: Figure 6—source data 1. [file elife-70464-fig6-data1.zip › Figure 6-source data 1/Figure 6A-T0400-Original.tif]

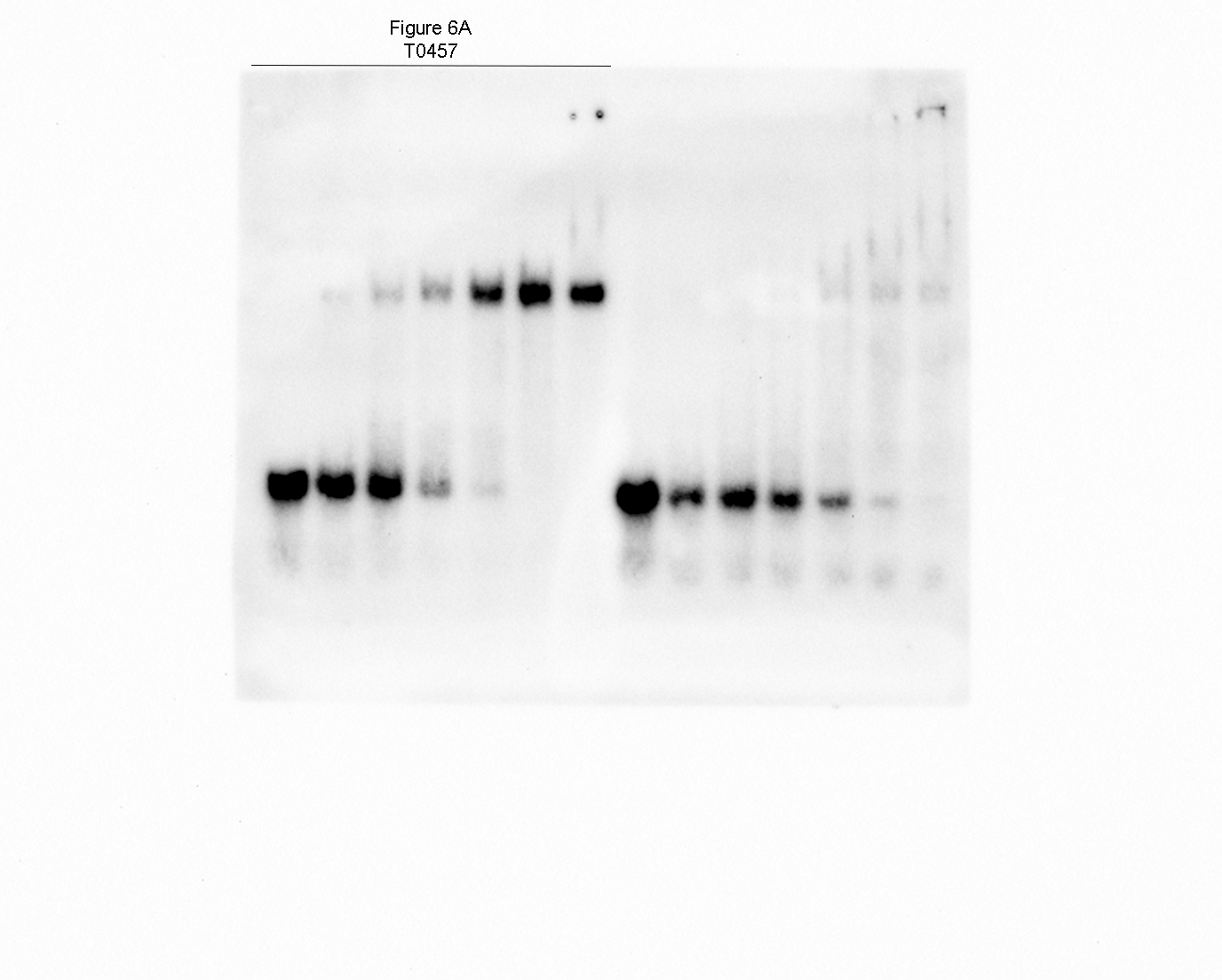

Supplement: Figure 6—source data 1. [file elife-70464-fig6-data1.zip › Figure 6-source data 1/Figure 6A-T0457-Labeled.tif]

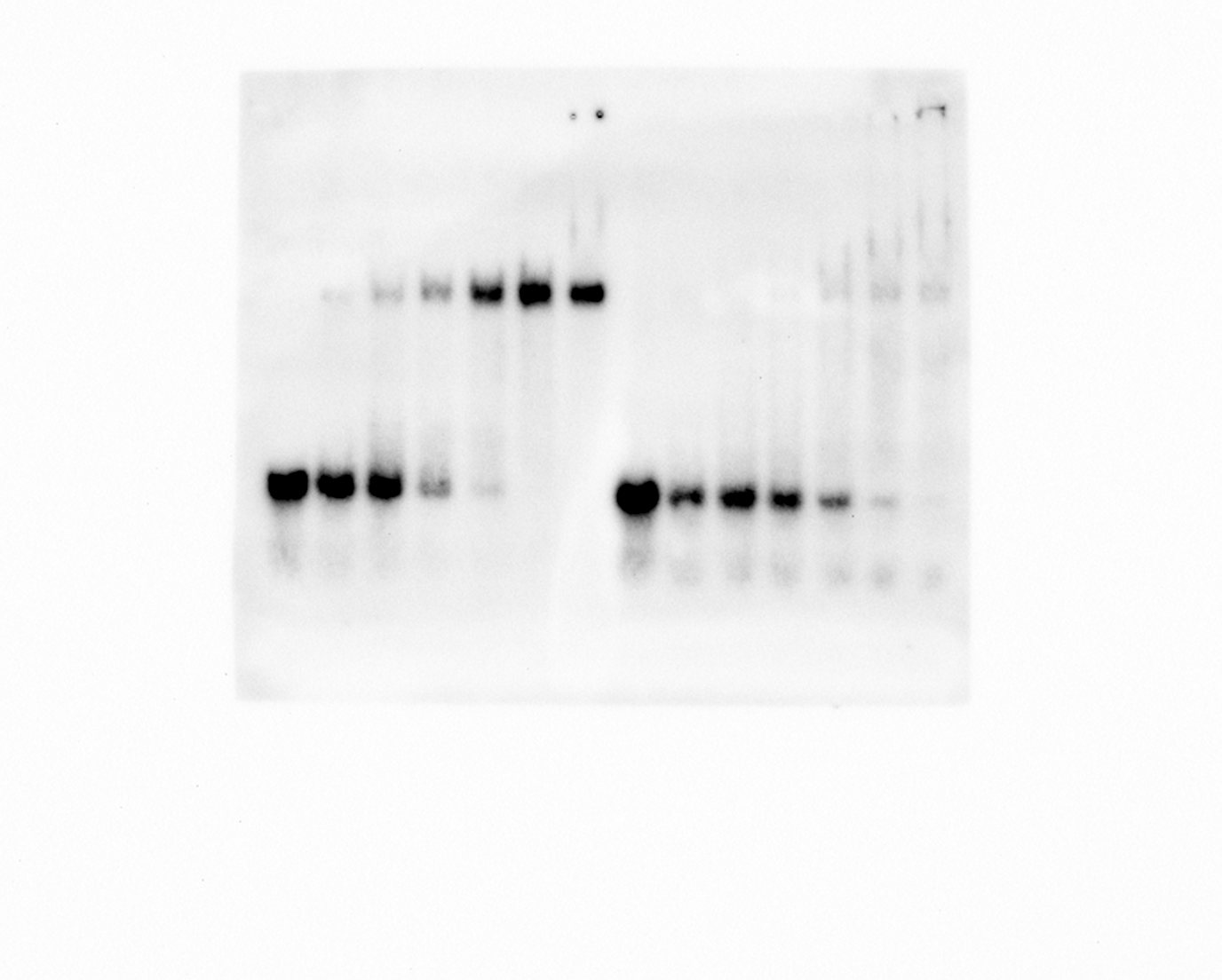

Supplement: Figure 6—source data 1. [file elife-70464-fig6-data1.zip › Figure 6-source data 1/Figure 6A-T0457-Original.tif]

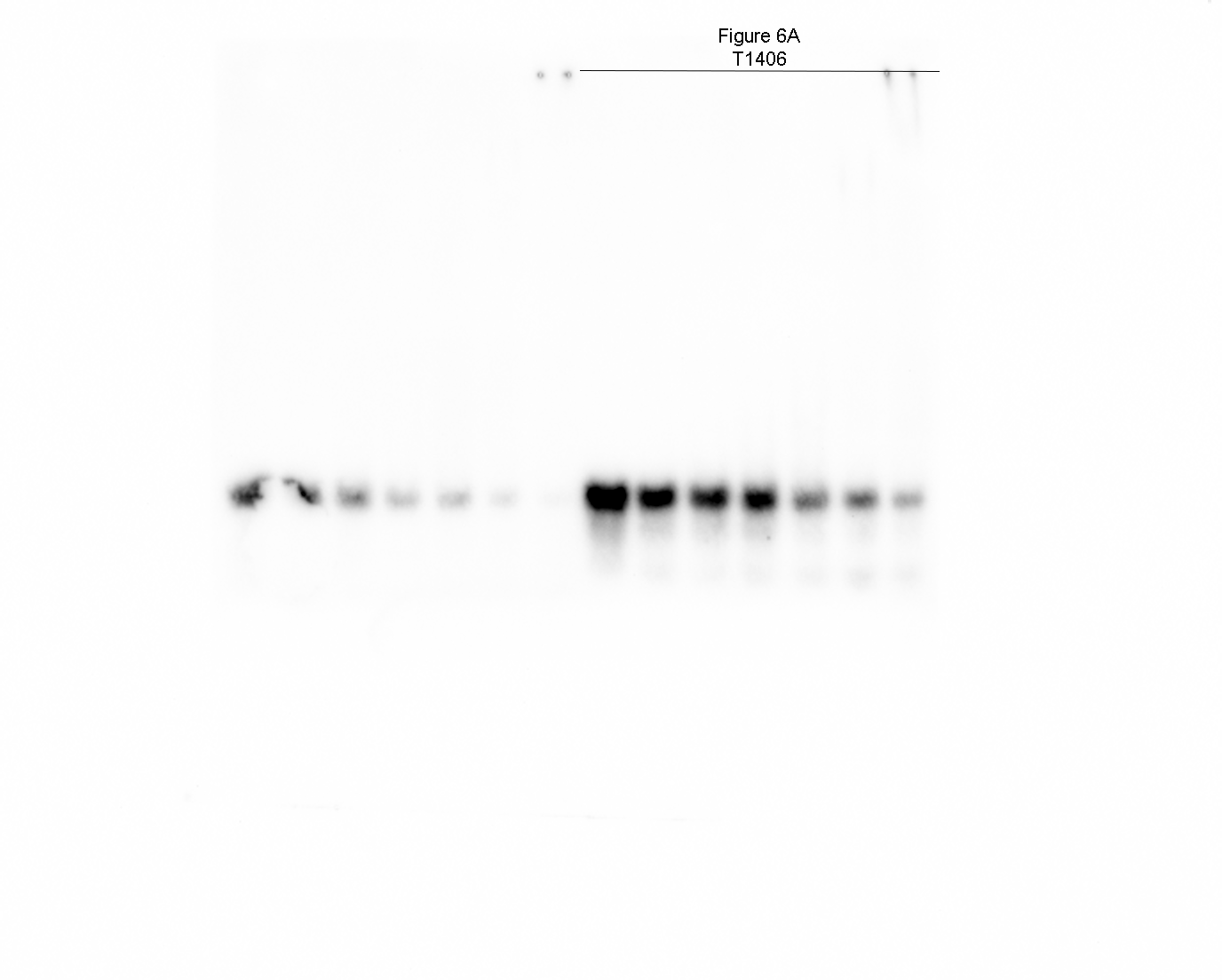

Supplement: Figure 6—source data 1. [file elife-70464-fig6-data1.zip › Figure 6-source data 1/Figure 6A-T1406-Labeled.tif]

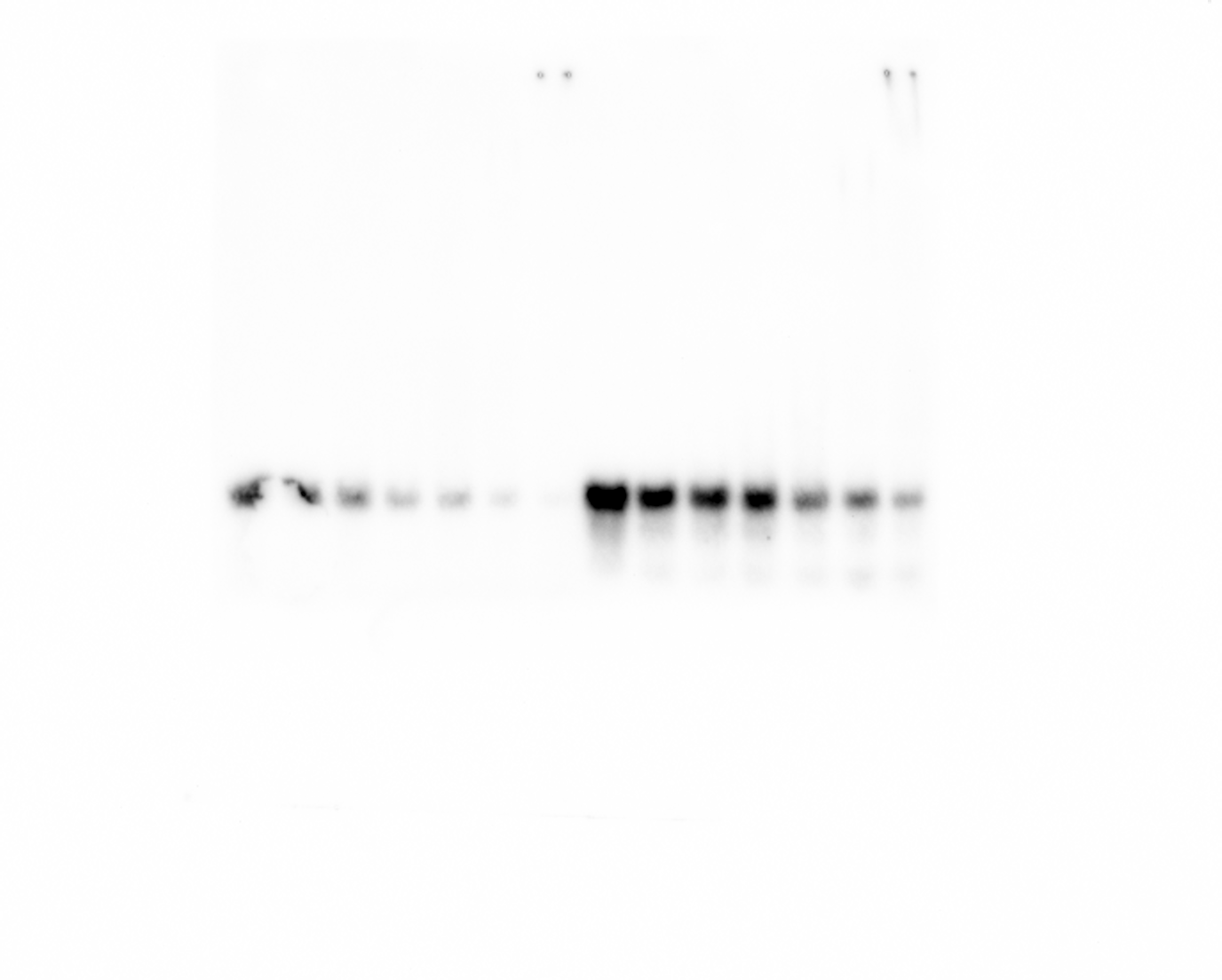

Supplement: Figure 6—source data 1. [file elife-70464-fig6-data1.zip › Figure 6-source data 1/Figure 6A-T1406-Original.tif]

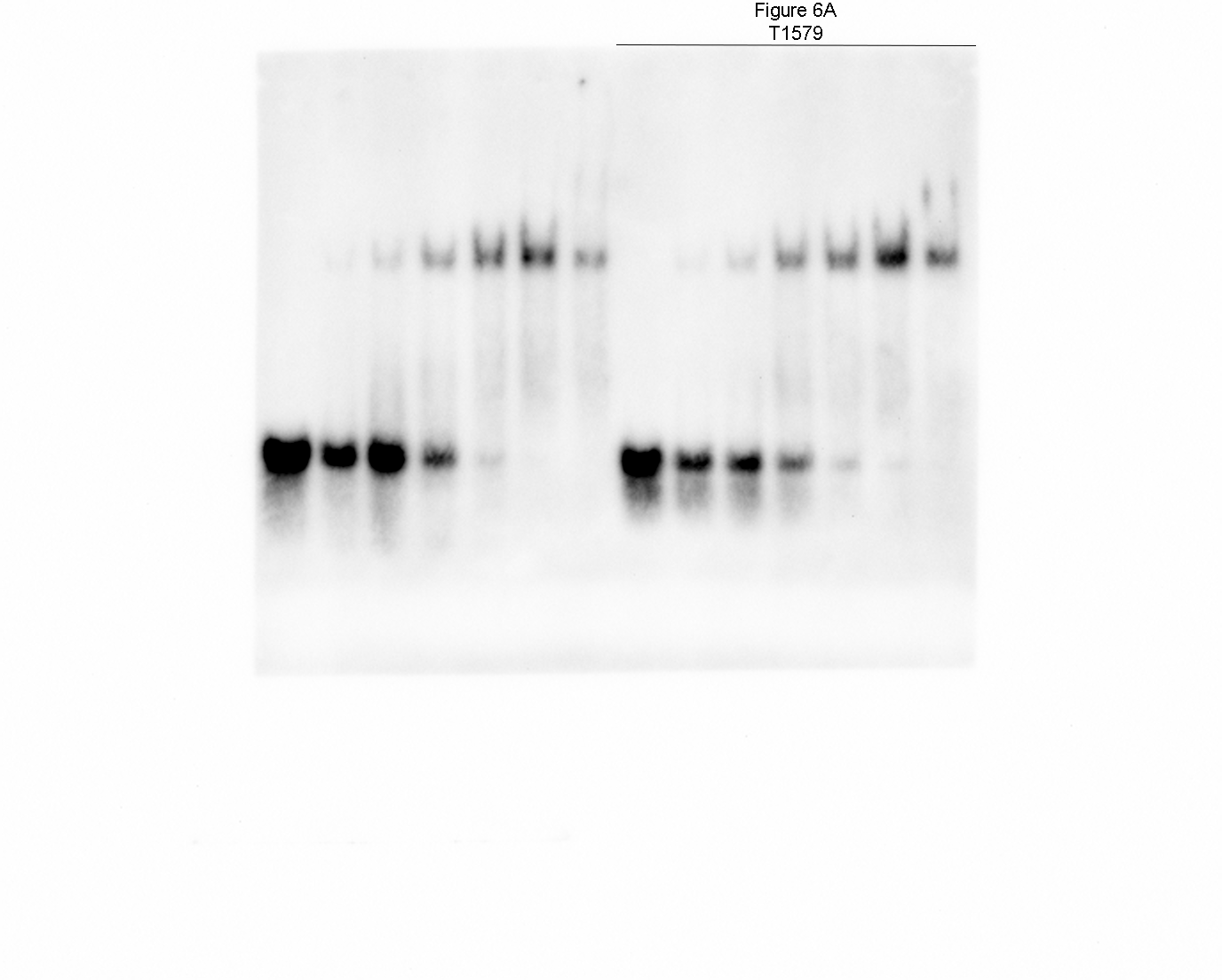

Supplement: Figure 6—source data 1. [file elife-70464-fig6-data1.zip › Figure 6-source data 1/Figure 6A-T1579-Labeled.tif]

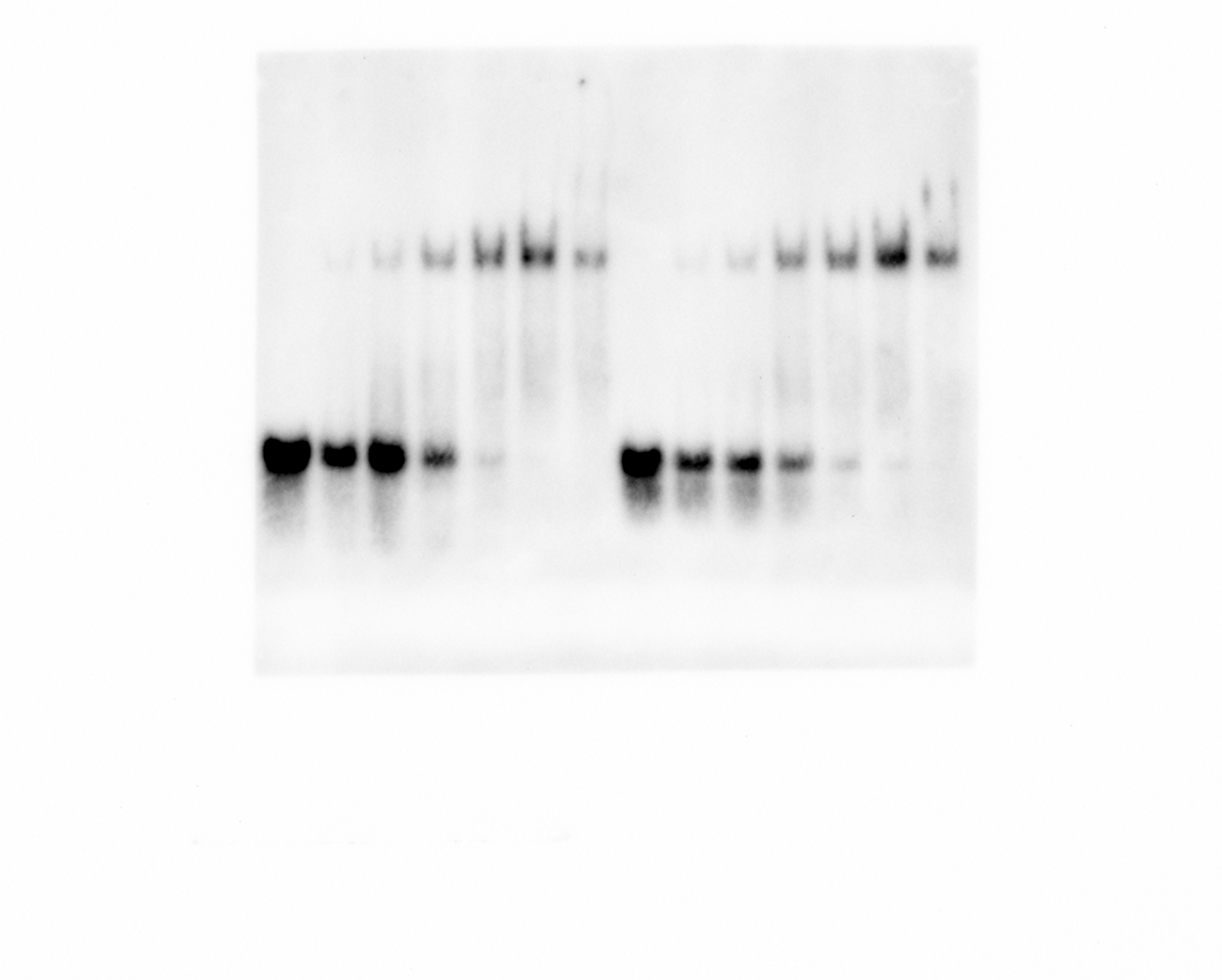

Supplement: Figure 6—source data 1. [file elife-70464-fig6-data1.zip › Figure 6-source data 1/Figure 6A-T1579-Original.tif]

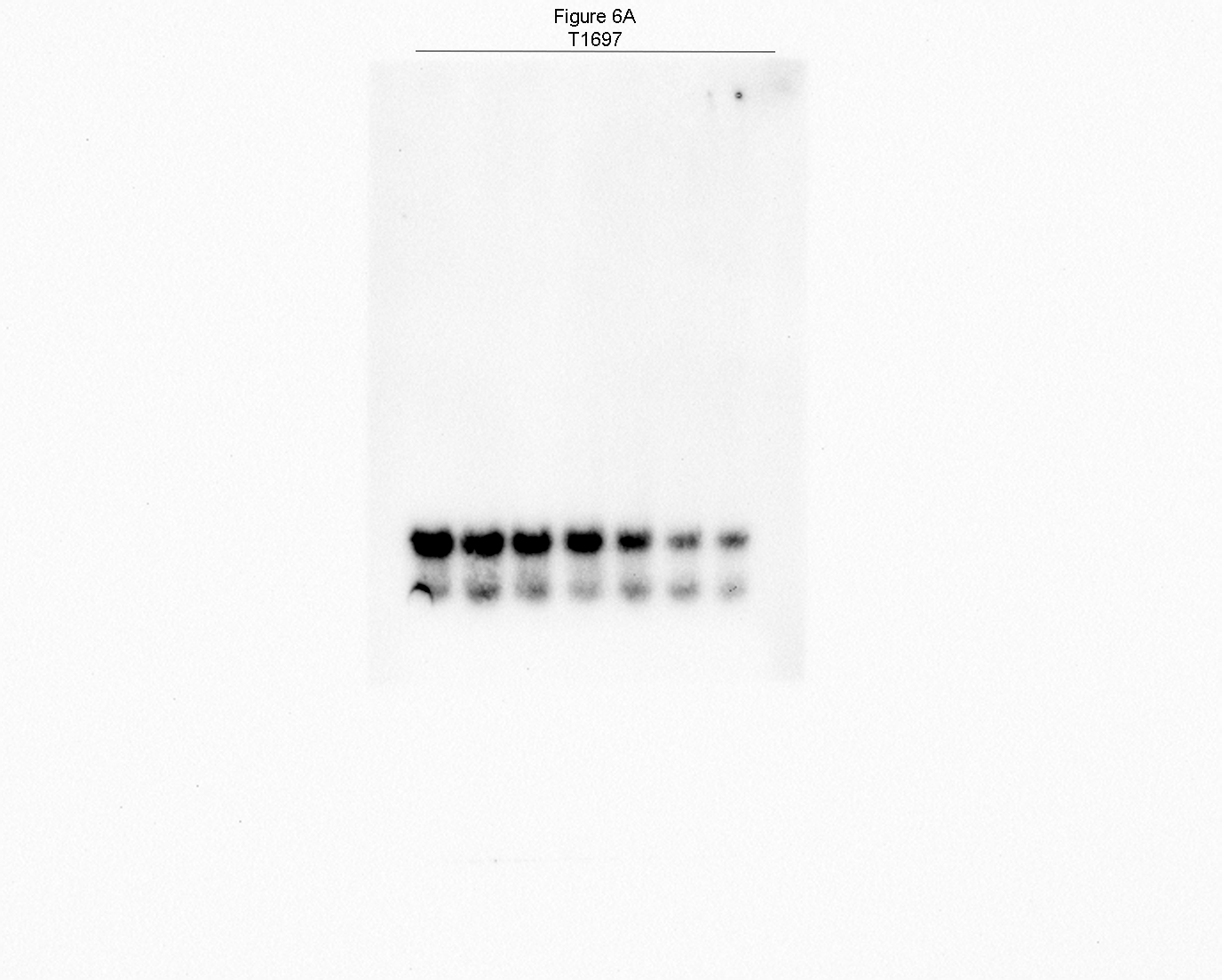

Supplement: Figure 6—source data 1. [file elife-70464-fig6-data1.zip › Figure 6-source data 1/Figure 6A-T1697-Labeled.tif]

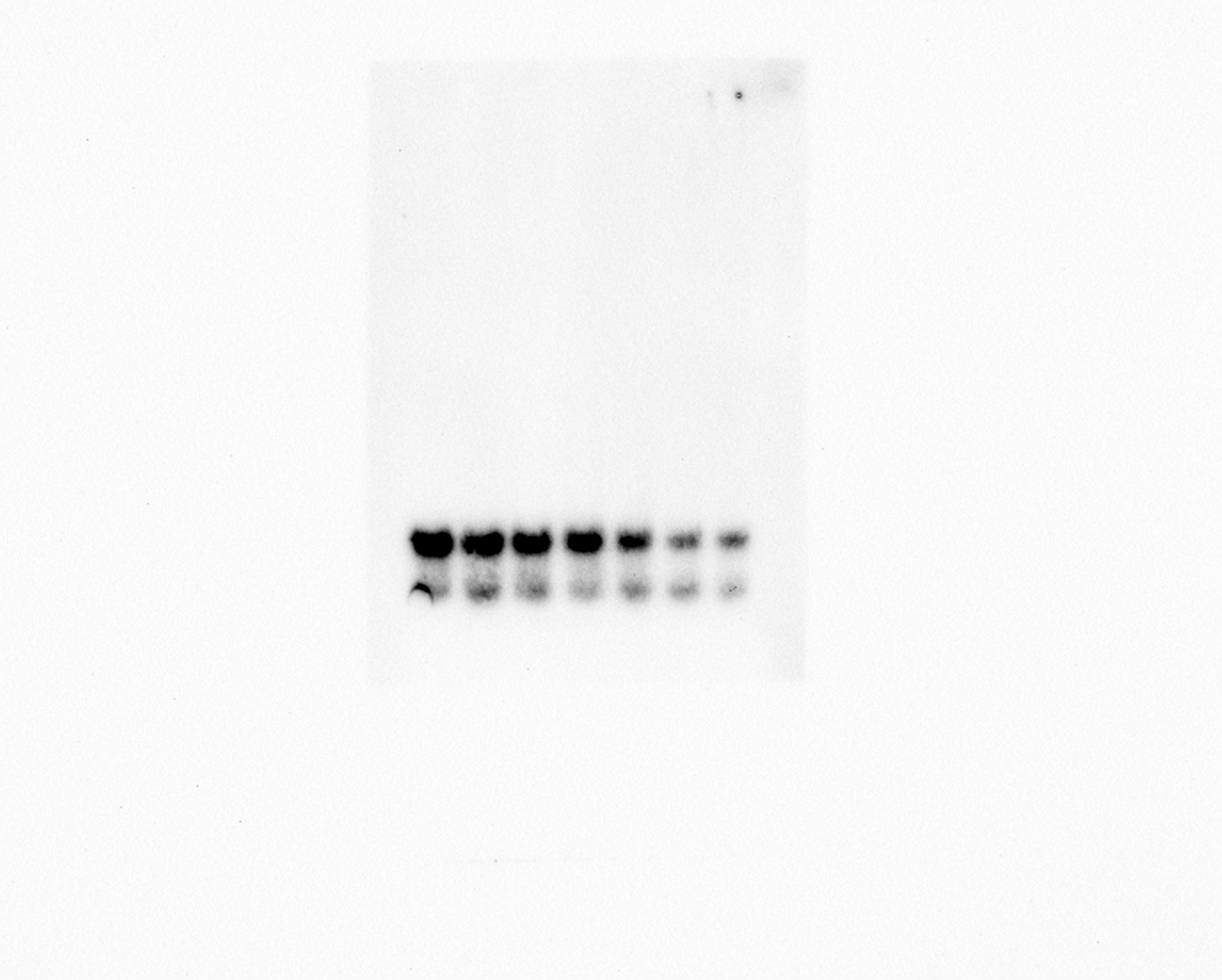

Supplement: Figure 6—source data 1. [file elife-70464-fig6-data1.zip › Figure 6-source data 1/Figure 6A-T1697-Original.tif]

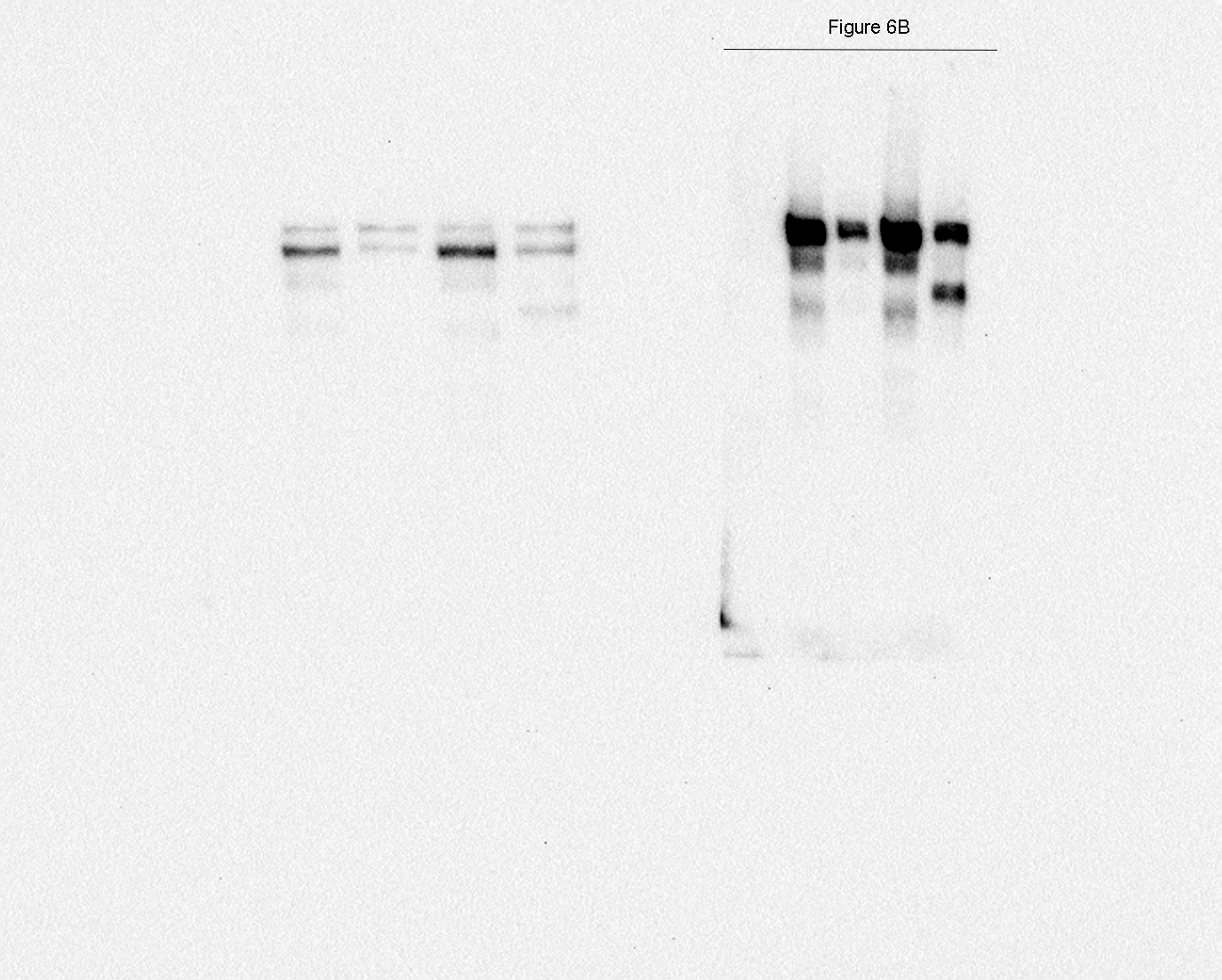

Supplement: Figure 6—source data 1. [file elife-70464-fig6-data1.zip › Figure 6-source data 1/Figure 6B-Labeled.tif]

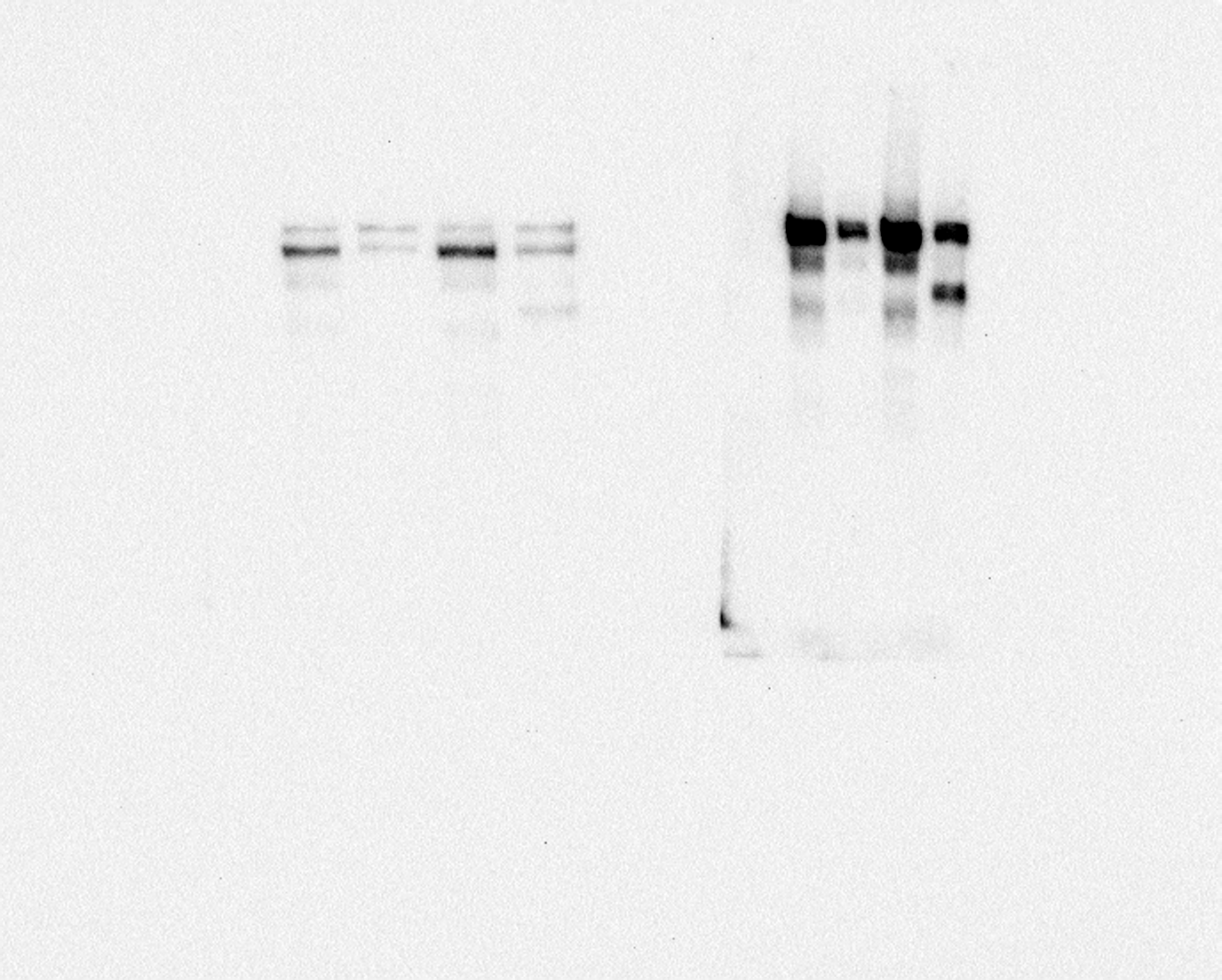

Supplement: Figure 6—source data 1. [file elife-70464-fig6-data1.zip › Figure 6-source data 1/Figure 6B-Original.tif]

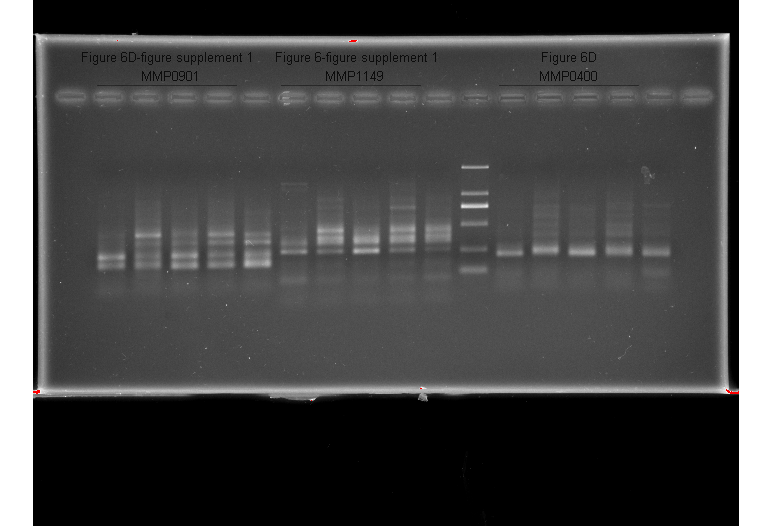

Supplement: Figure 6—source data 1. [file elife-70464-fig6-data1.zip › Figure 6-source data 1/Figure 6D-MMP0400 Figure 6-figure supplement 1-MMP0901 MMP1149-Labeled.tif]

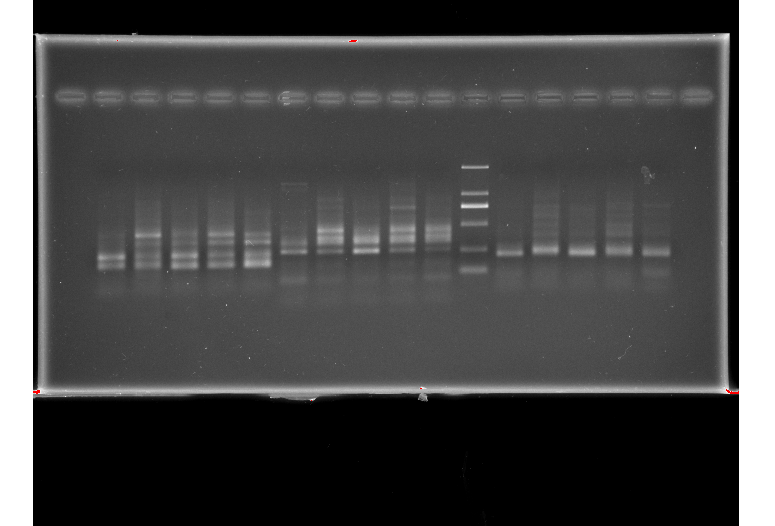

Supplement: Figure 6—source data 1. [file elife-70464-fig6-data1.zip › Figure 6-source data 1/Figure 6D-MMP0400 Figure 6-figure supplement 1-MMP0901 MMP1149-Original.tif]

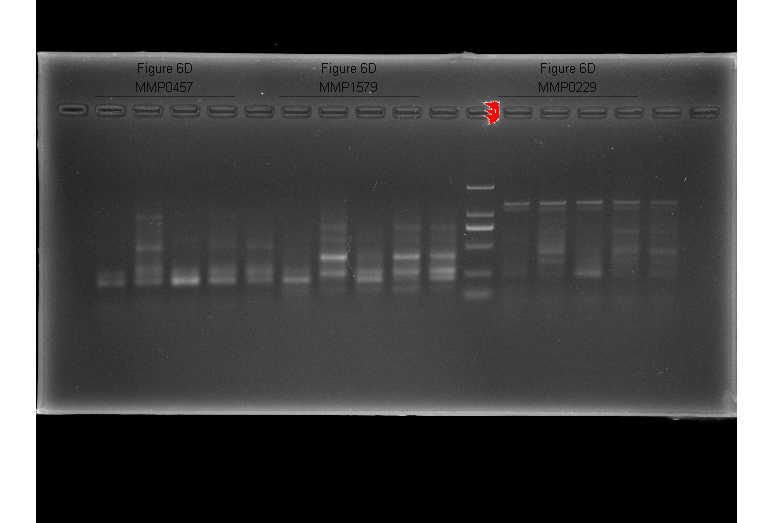

Supplement: Figure 6—source data 1. [file elife-70464-fig6-data1.zip › Figure 6-source data 1/Figure 6D-MMP0457 1579 0229-Labeled.tif]

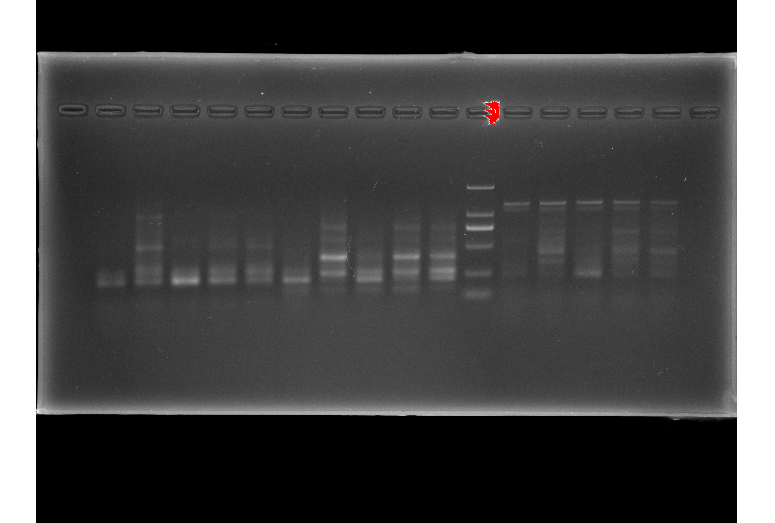

Supplement: Figure 6—source data 1. [file elife-70464-fig6-data1.zip › Figure 6-source data 1/Figure 6D-MMP0457 1579 0229-Original.tif]

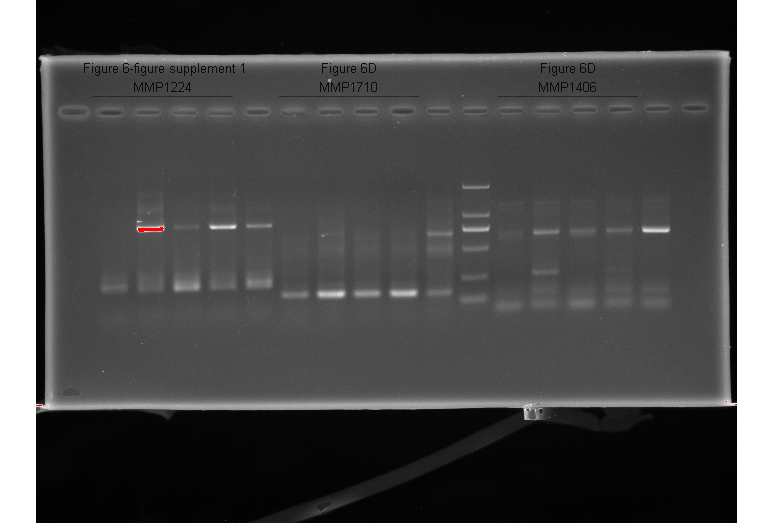

Supplement: Figure 6—source data 1. [file elife-70464-fig6-data1.zip › Figure 6-source data 1/Figure 6D-MMP1710 1406 Figure 6-figure supplement-MMP1224-Labeled.tif]

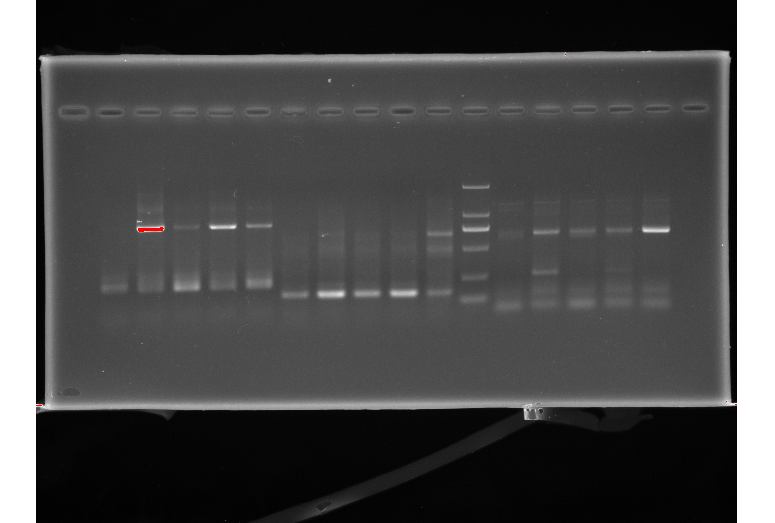

Supplement: Figure 6—source data 1. [file elife-70464-fig6-data1.zip › Figure 6-source data 1/Figure 6D-MMP1710 1406 Figure 6-figure supplement-MMP1224-Original.tif]

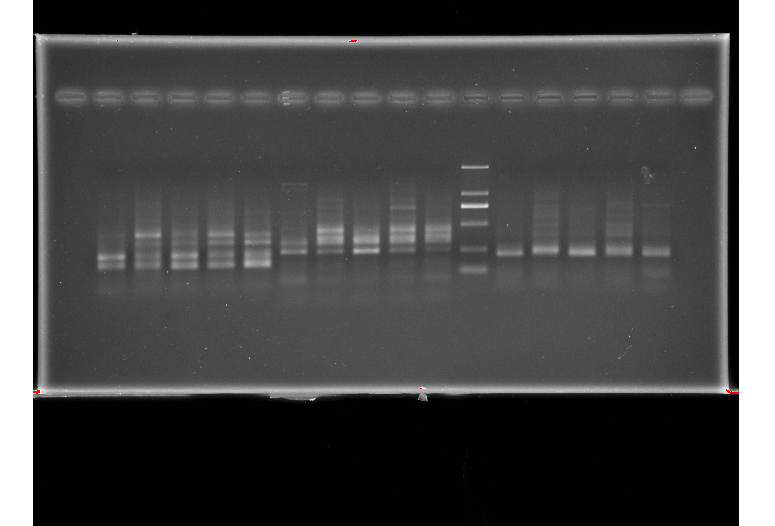

Supplement: Figure 6—figure supplement 1—source data 1. [file elife-70464-fig6-figsupp1-data1.zip › Figure 6-figure supplement 1-source data 1/Figure 6D-MMP0400 Figure 6-figure supplement 1-MMP0901 MMP1149-Original.tif]

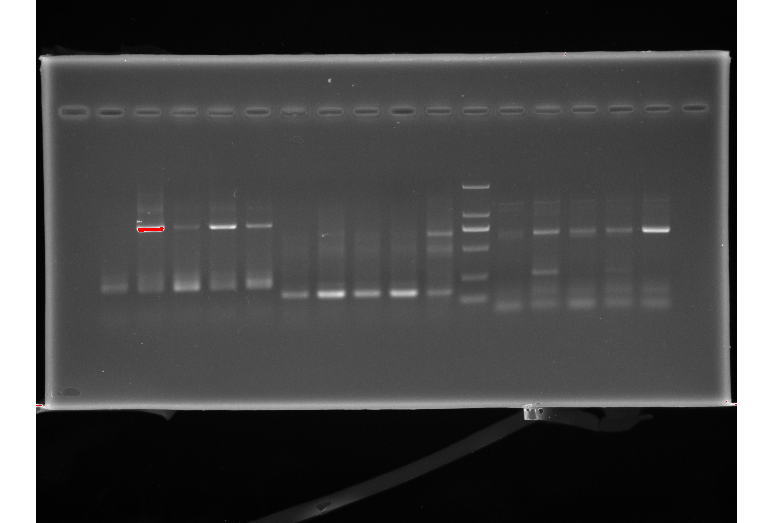

Supplement: Figure 6—figure supplement 1—source data 1. [file elife-70464-fig6-figsupp1-data1.zip › Figure 6-figure supplement 1-source data 1/Figure 6D-MMP1710 1406 Figure 6-figure supplement-MMP1224-Original.tif]

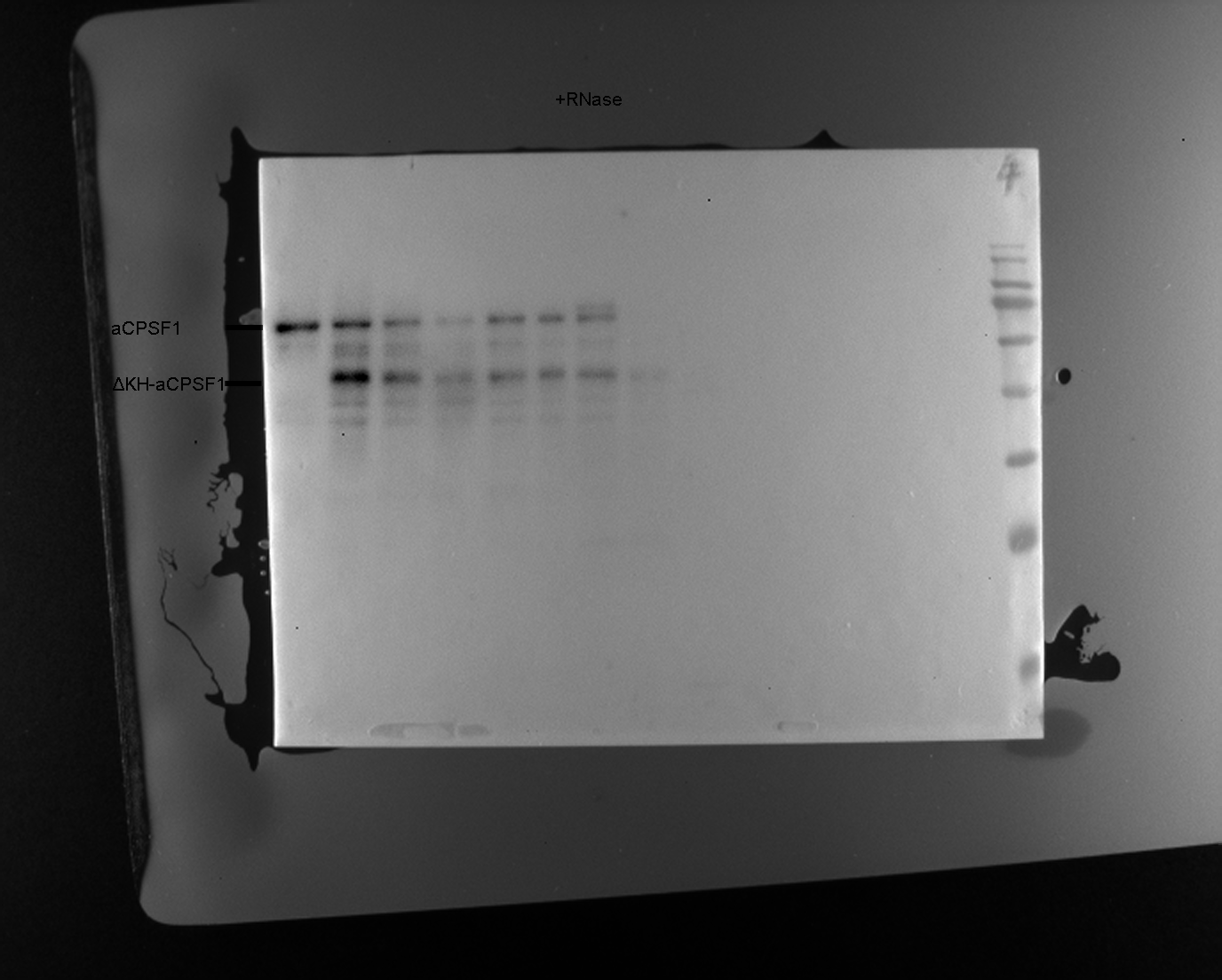

Supplement: Figure 6—figure supplement 2—source data 1. [file elife-70464-fig6-figsupp2-data1.zip › Figure 6-figure supplement 2-source data 1/Figure 6- figure supplement 2 +RNase- aCPSF1 ΔKH-aCPSF1-Labeled.tif]

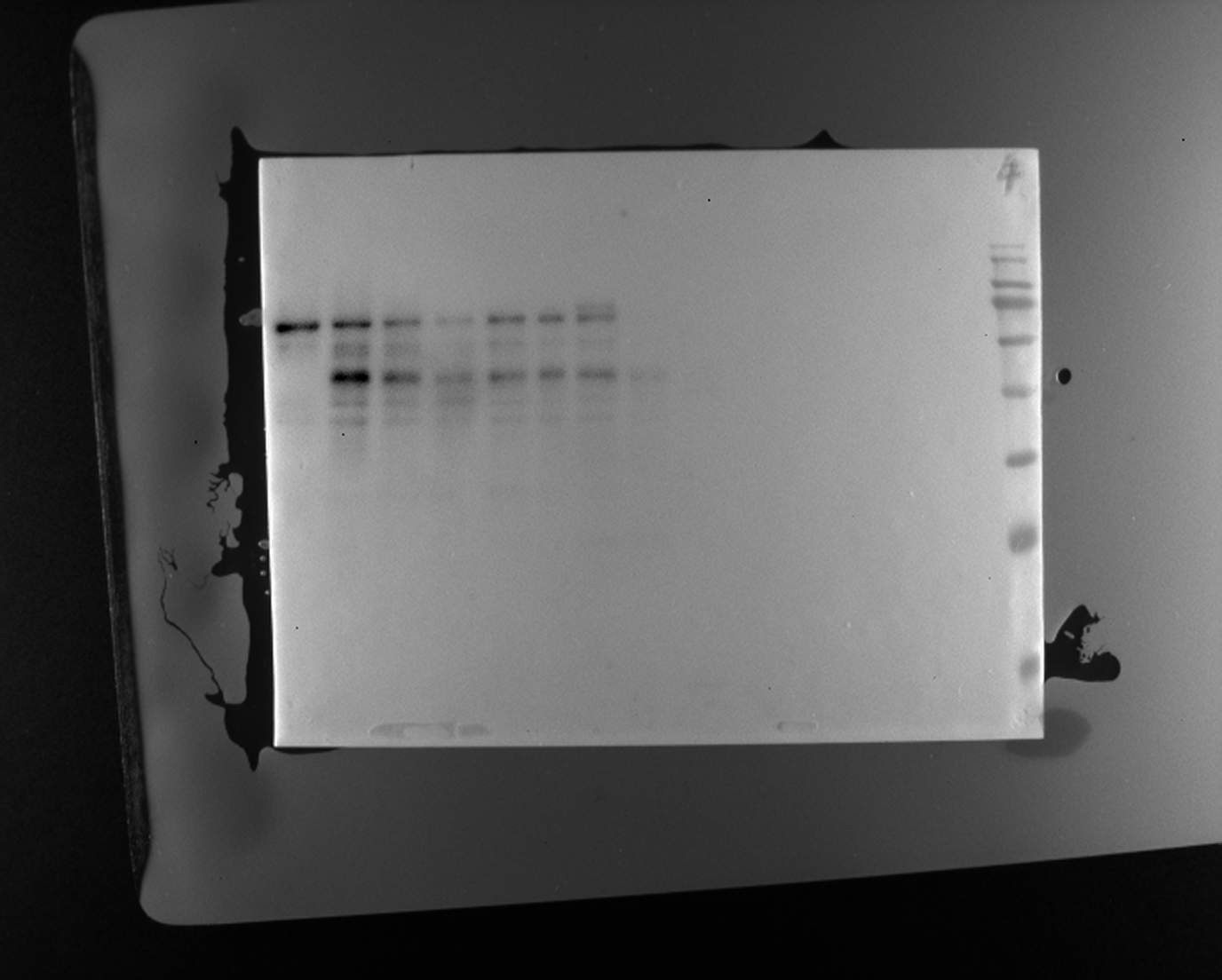

Supplement: Figure 6—figure supplement 2—source data 1. [file elife-70464-fig6-figsupp2-data1.zip › Figure 6-figure supplement 2-source data 1/Figure 6- figure supplement 2 +RNase- aCPSF1 ΔKH-aCPSF1-Original.tif]

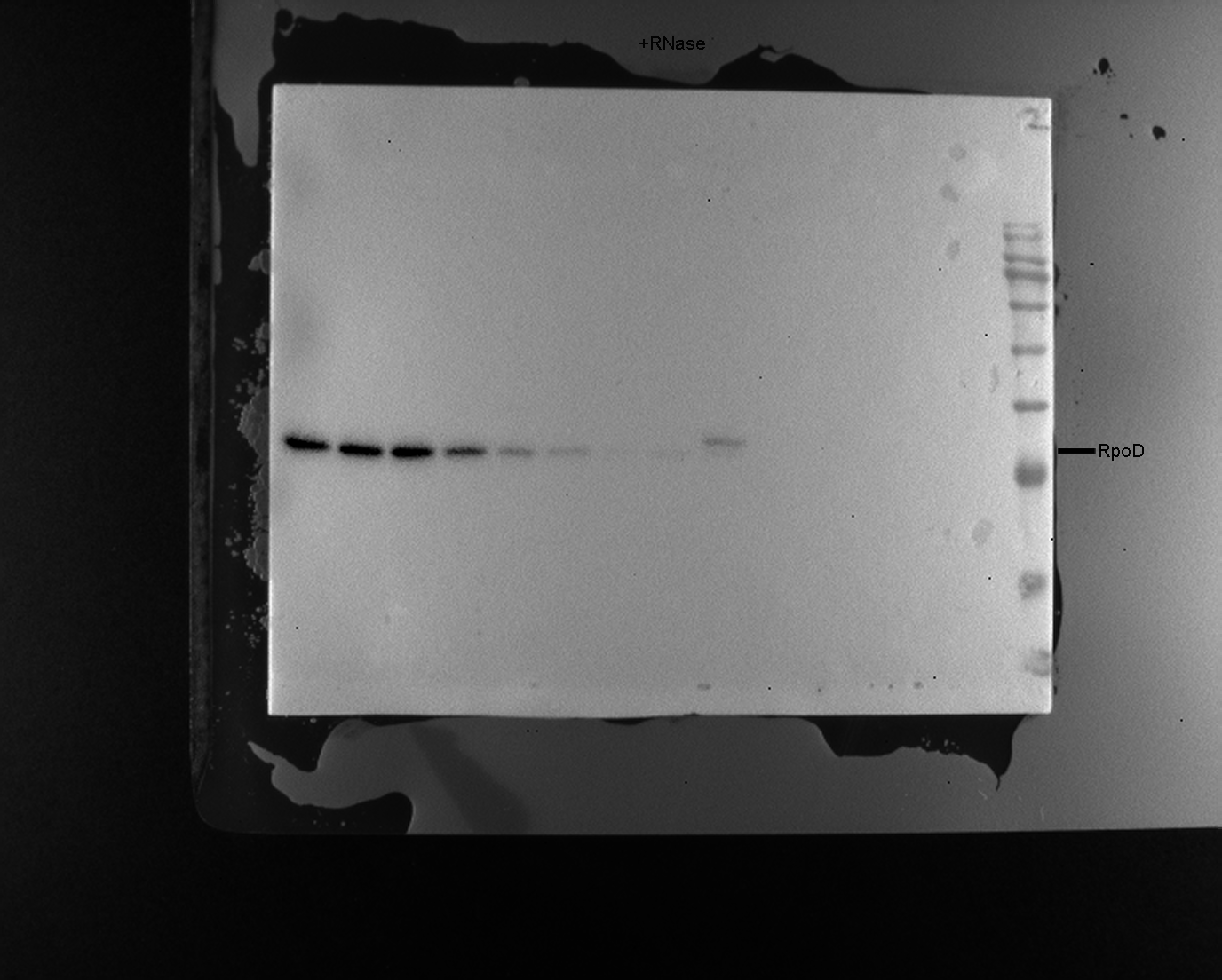

Supplement: Figure 6—figure supplement 2—source data 1. [file elife-70464-fig6-figsupp2-data1.zip › Figure 6-figure supplement 2-source data 1/Figure 6- figure supplement 2 +RNase-RpoD-Labeled.tif]

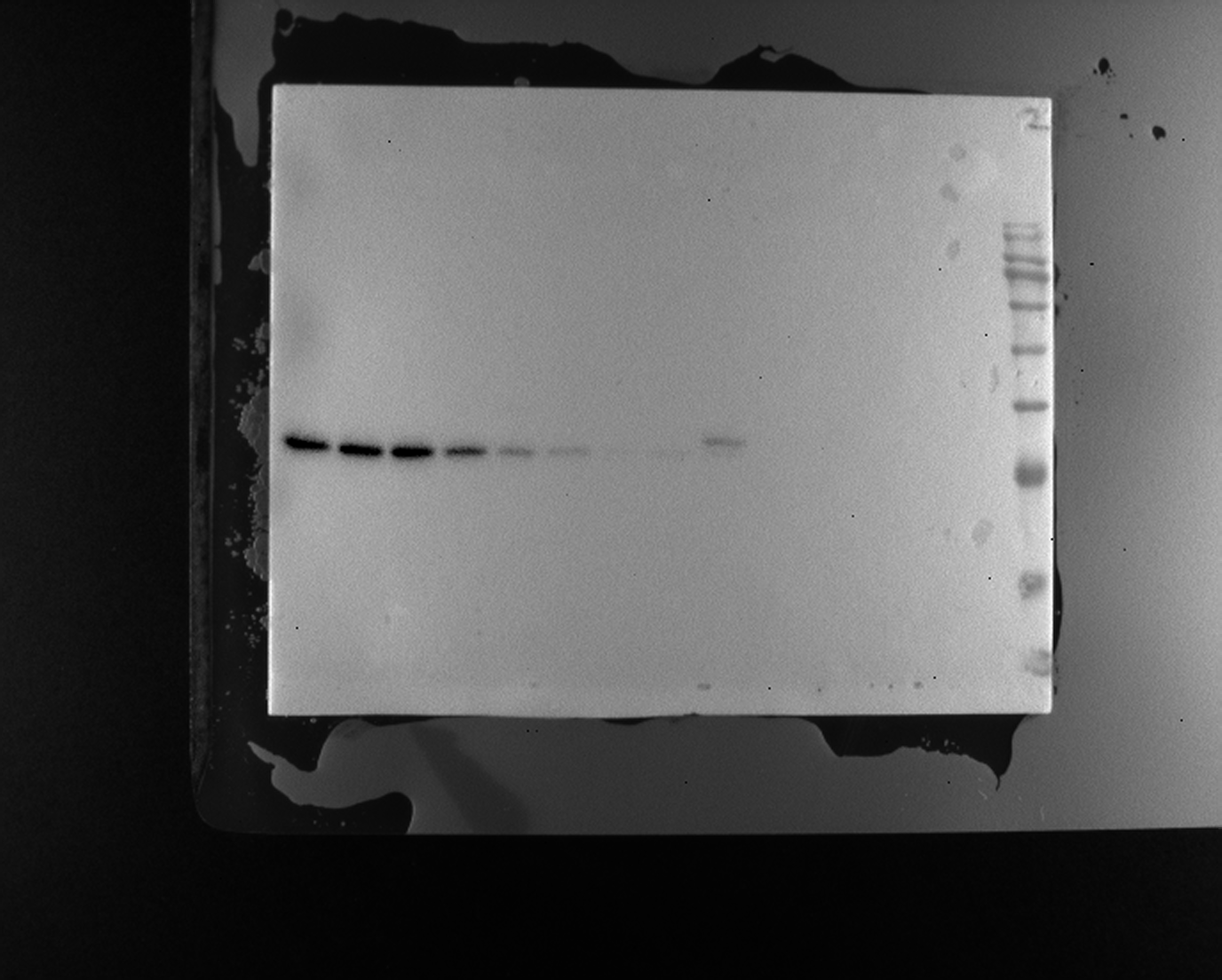

Supplement: Figure 6—figure supplement 2—source data 1. [file elife-70464-fig6-figsupp2-data1.zip › Figure 6-figure supplement 2-source data 1/Figure 6- figure supplement 2 +RNase-RpoD-Original.tif]

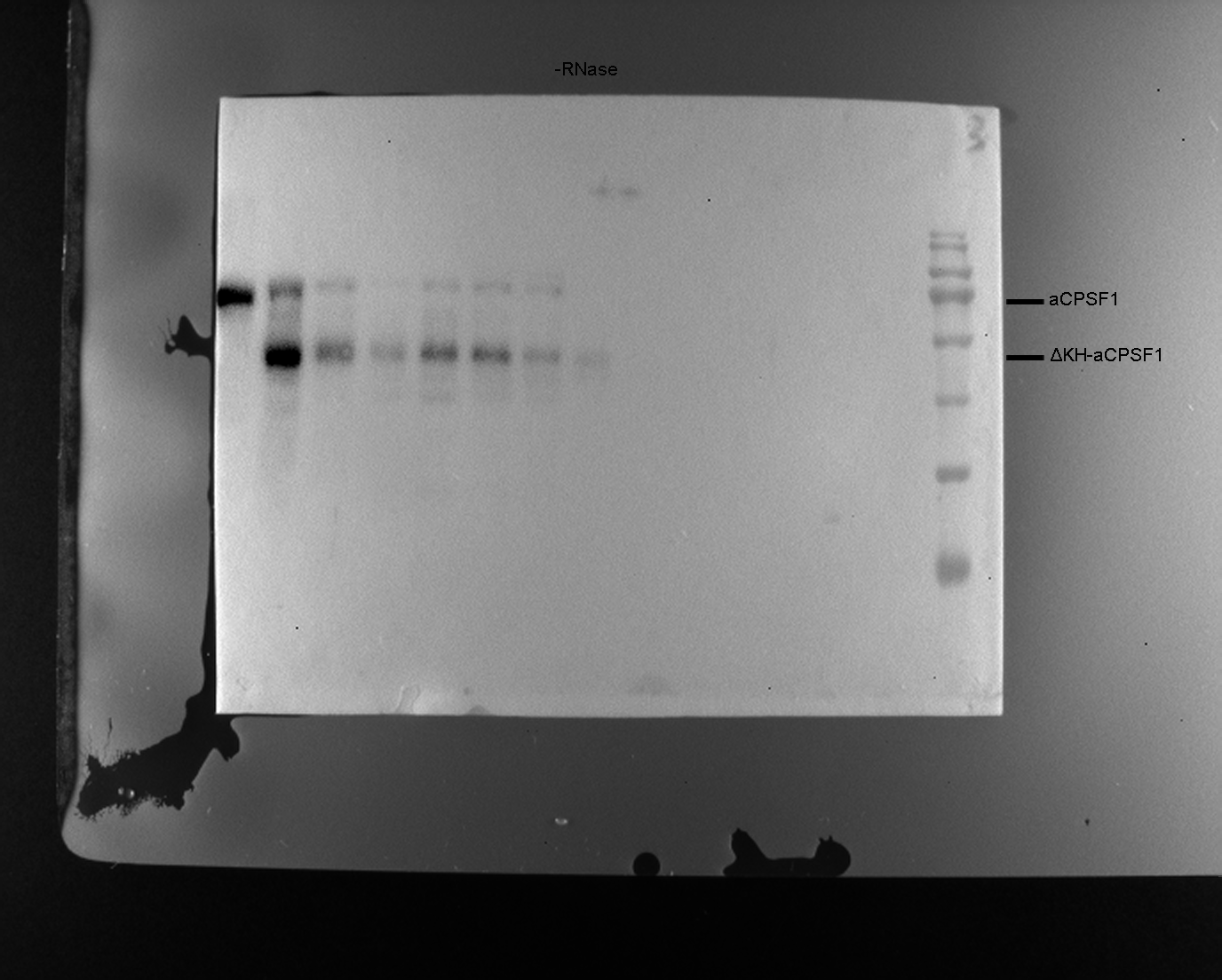

Supplement: Figure 6—figure supplement 2—source data 1. [file elife-70464-fig6-figsupp2-data1.zip › Figure 6-figure supplement 2-source data 1/Figure 6- figure supplement 2 -RNase- aCPSF1 ΔKH-aCPSF1-Labeled.tif]

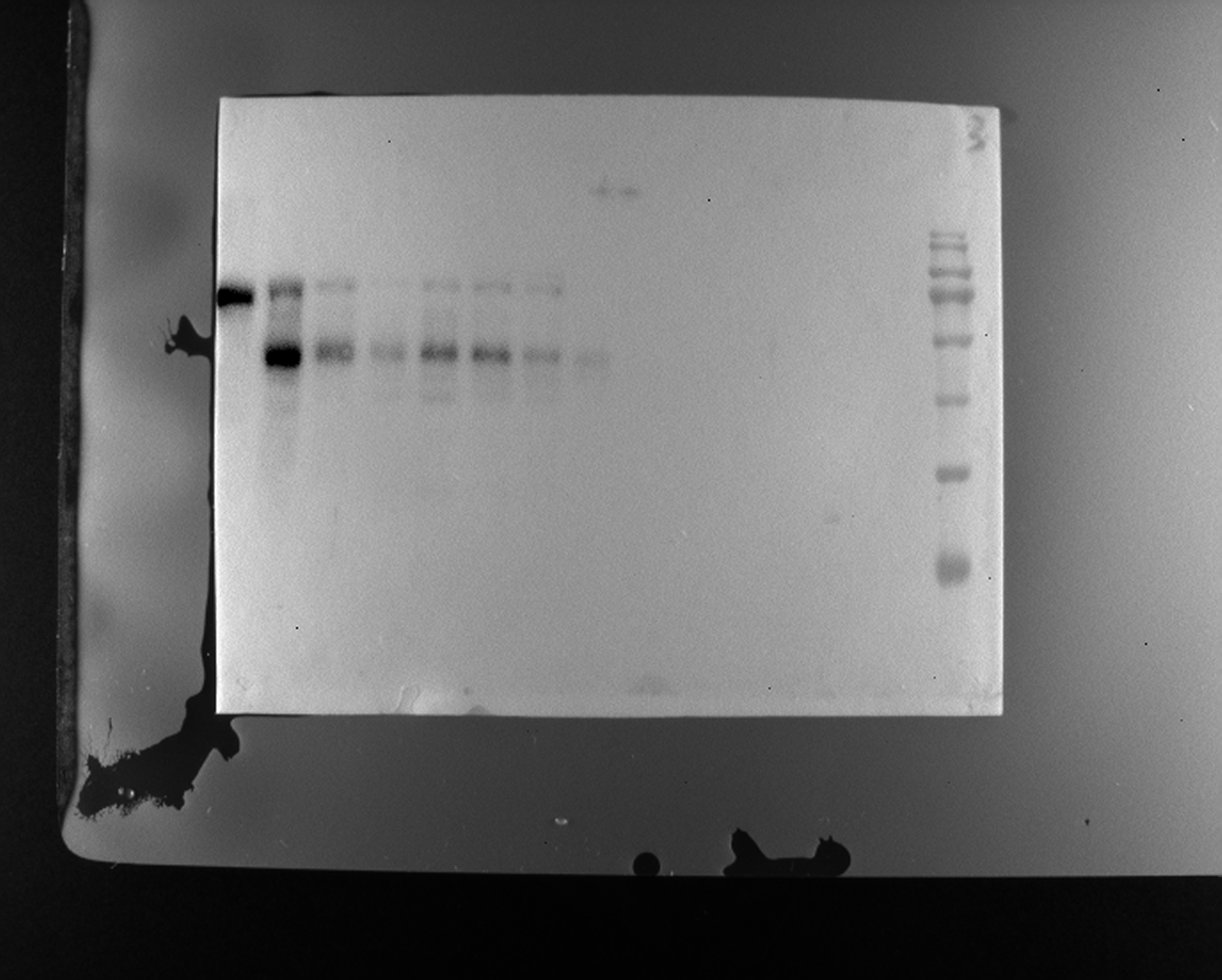

Supplement: Figure 6—figure supplement 2—source data 1. [file elife-70464-fig6-figsupp2-data1.zip › Figure 6-figure supplement 2-source data 1/Figure 6- figure supplement 2 -RNase- aCPSF1 ΔKH-aCPSF1-Original.tif]

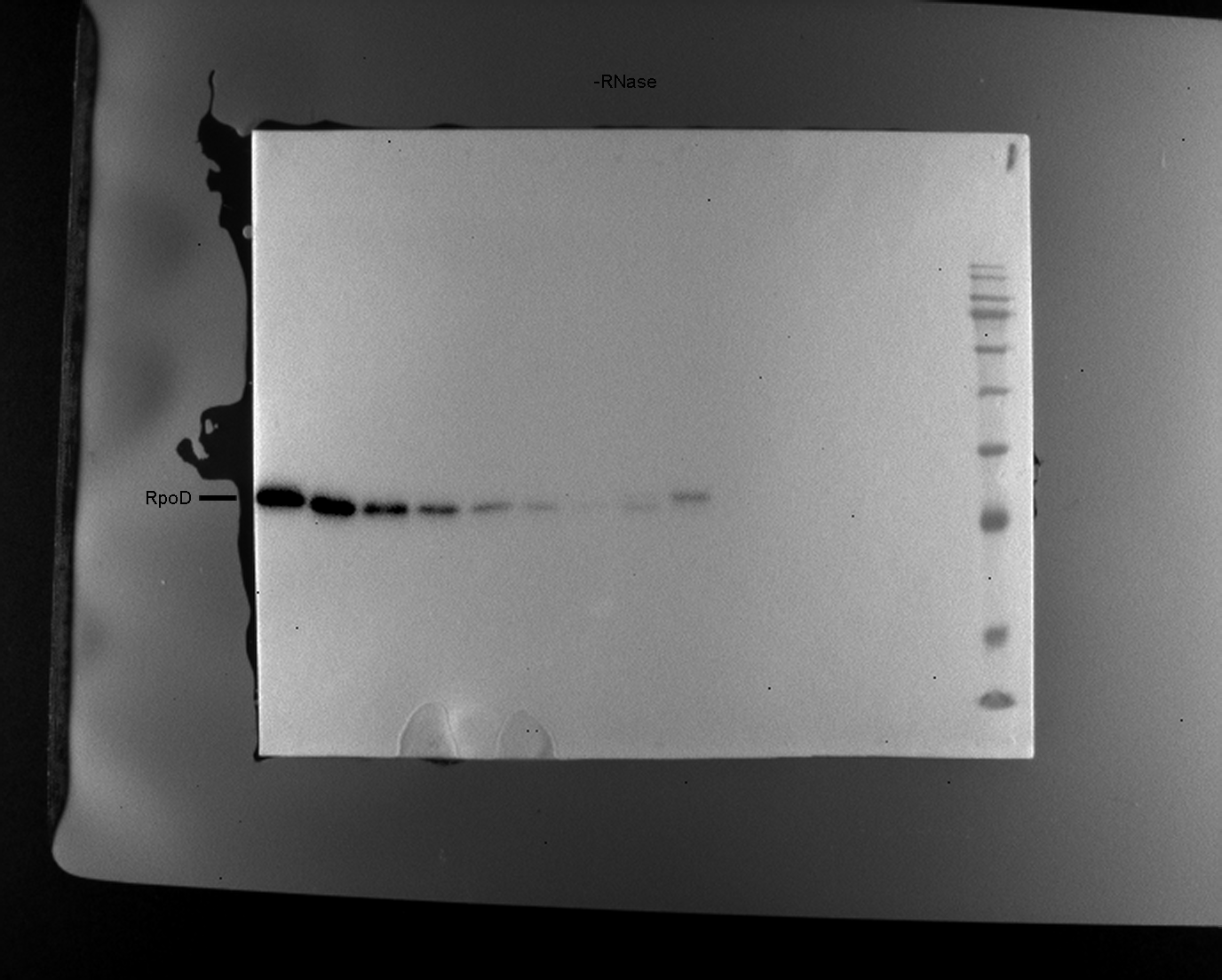

Supplement: Figure 6—figure supplement 2—source data 1. [file elife-70464-fig6-figsupp2-data1.zip › Figure 6-figure supplement 2-source data 1/Figure 6- figure supplement 2 -RNase-RpoD-Labeled.tif]

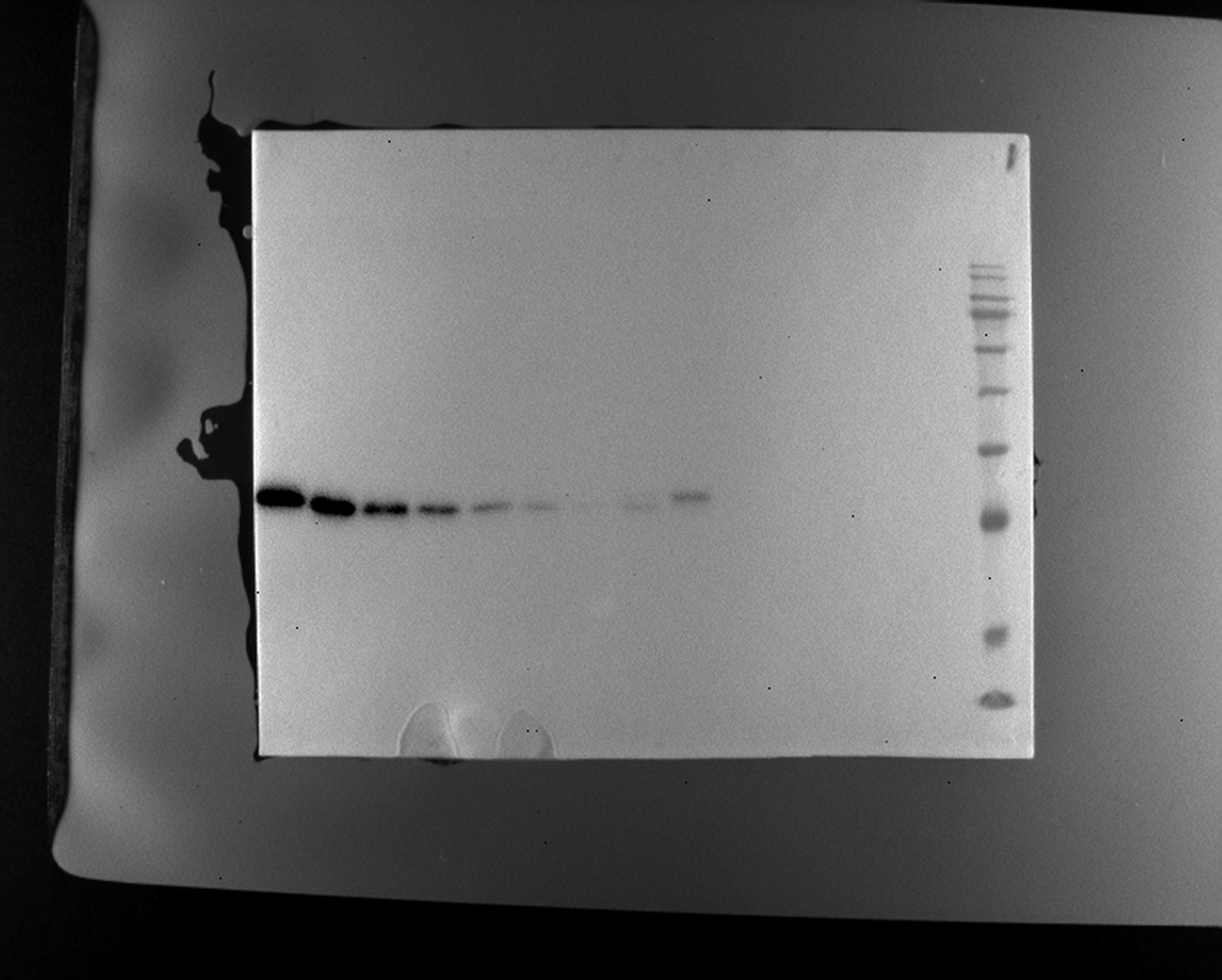

Supplement: Figure 6—figure supplement 2—source data 1. [file elife-70464-fig6-figsupp2-data1.zip › Figure 6-figure supplement 2-source data 1/Figure 6- figure supplement 2 -RNase-RpoD-Original.tif]

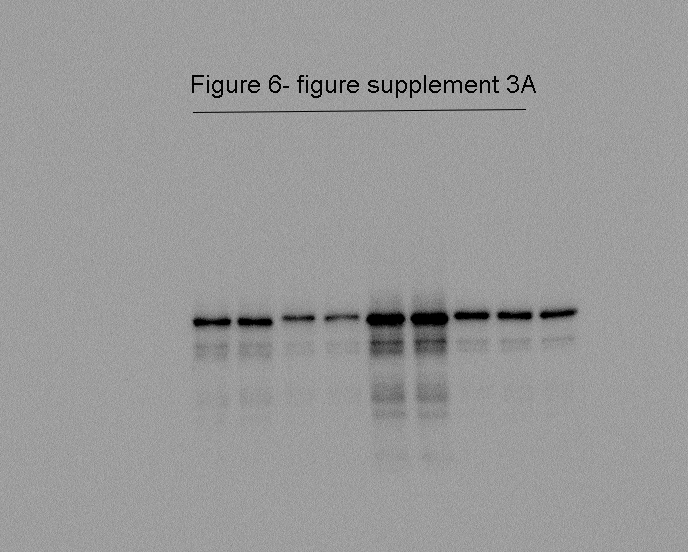

Supplement: Figure 6—figure supplement 3—source data 1. [file elife-70464-fig6-figsupp3-data1.zip › Figure 6-figure supplement 3-source data 1/Figure 6- figure supplement 3A-Labeled.tif]

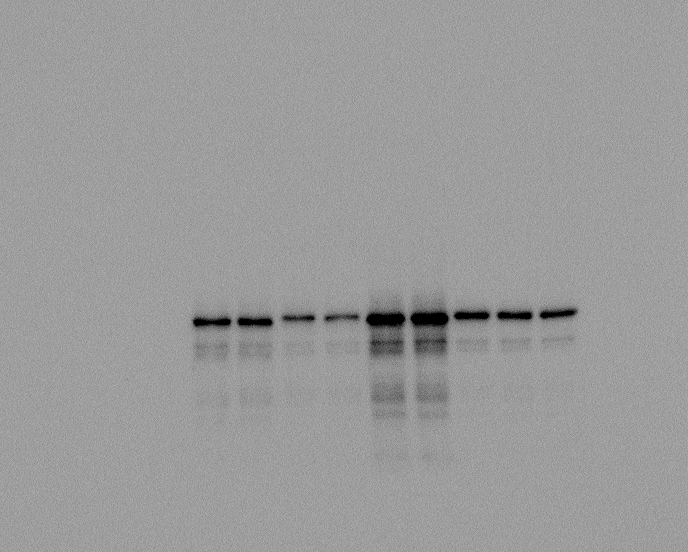

Supplement: Figure 6—figure supplement 3—source data 1. [file elife-70464-fig6-figsupp3-data1.zip › Figure 6-figure supplement 3-source data 1/Figure 6- figure supplement 3A-Original.tif]

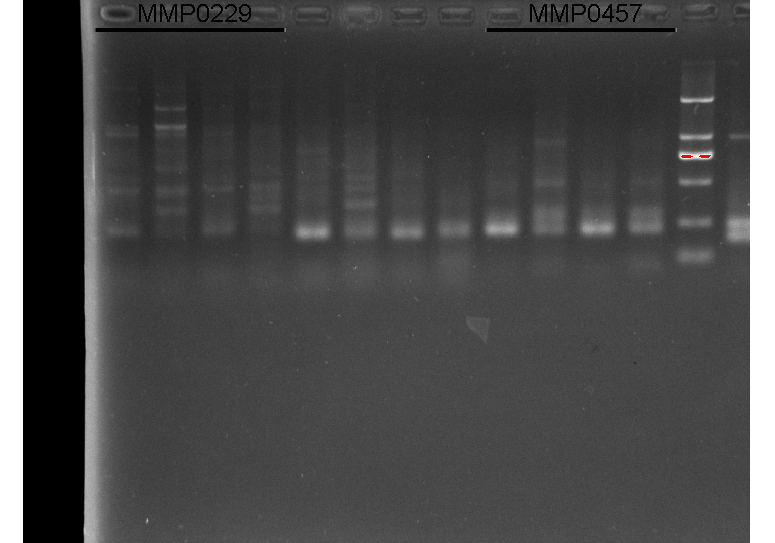

Supplement: Figure 6—figure supplement 3—source data 1. [file elife-70464-fig6-figsupp3-data1.zip › Figure 6-figure supplement 3-source data 1/Figure 6-figure supplement 3C-MMP0457 MMP0229-Labeled.tif]

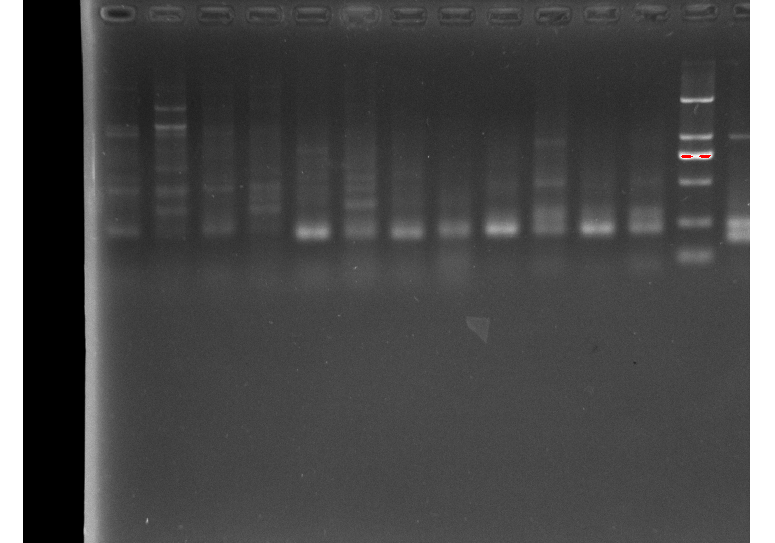

Supplement: Figure 6—figure supplement 3—source data 1. [file elife-70464-fig6-figsupp3-data1.zip › Figure 6-figure supplement 3-source data 1/Figure 6-figure supplement 3C-MMP0457 MMP0229-Original.tif]

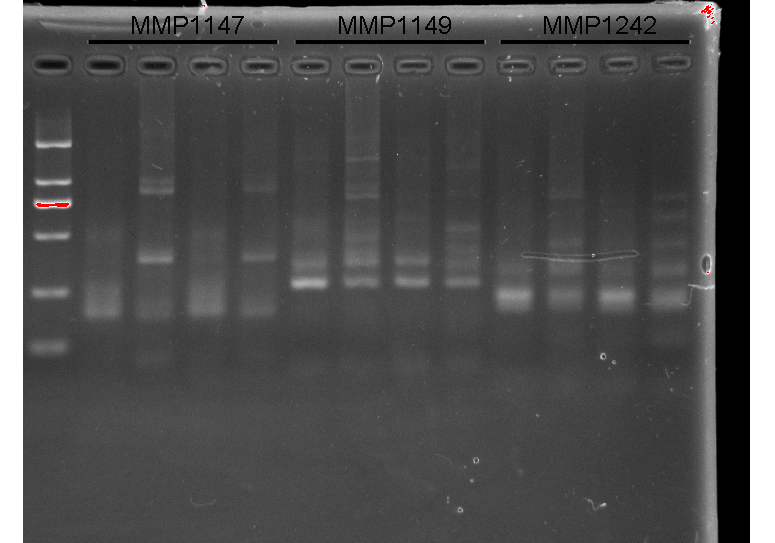

Supplement: Figure 6—figure supplement 3—source data 1. [file elife-70464-fig6-figsupp3-data1.zip › Figure 6-figure supplement 3-source data 1/Figure 6-figure supplement 3C-MMP114 MMP1149 MMP1242-Labeled.tif]

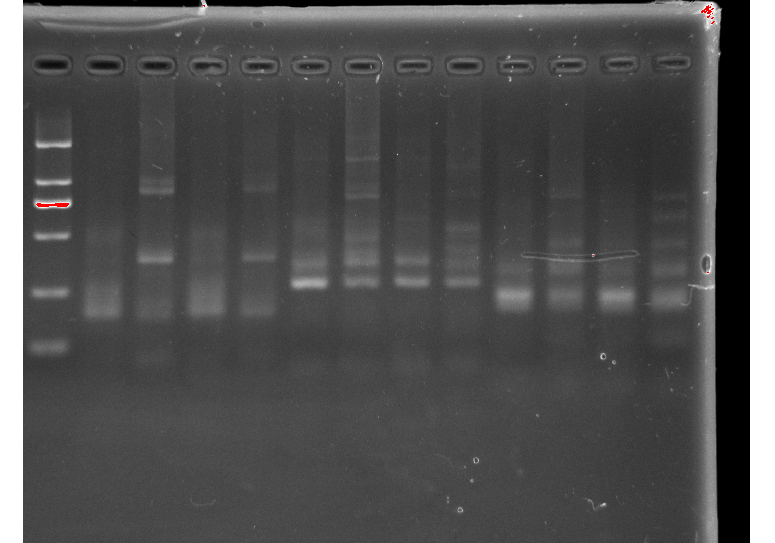

Supplement: Figure 6—figure supplement 3—source data 1. [file elife-70464-fig6-figsupp3-data1.zip › Figure 6-figure supplement 3-source data 1/Figure 6-figure supplement 3C-MMP114 MMP1149 MMP1242-Original.tif]
