# Supplementary figures and images for: aCPSF1 cooperates with terminator U-tract to dictate archaeal transcription termination efficacy (part 2 of 2)
Source: eLife. 2021 Dec 29;10:e70464. doi: 10.7554/eLife.70464 (PMC8716108; doi:10.7554/eLife.70464)

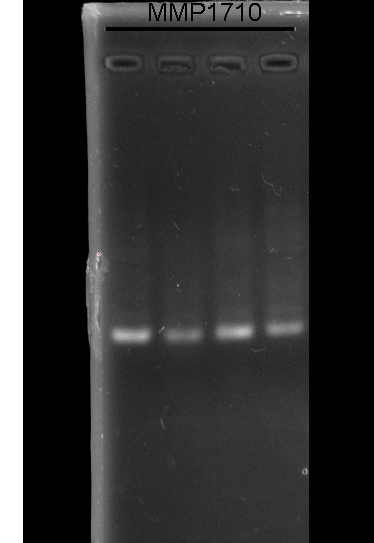

Supplement: Figure 6—figure supplement 3—source data 1. [file elife-70464-fig6-figsupp3-data1.zip › Figure 6-figure supplement 3-source data 1/Figure 6-figure supplement 3C-MMP1710-Labeled.tif]

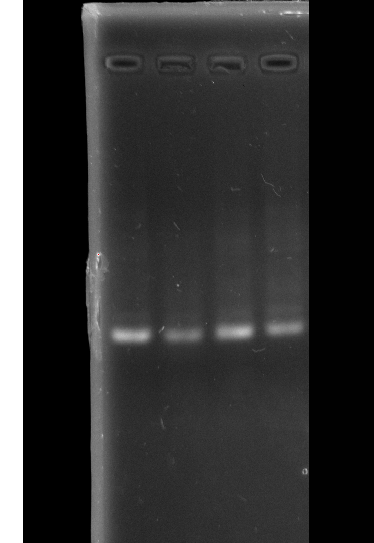

Supplement: Figure 6—figure supplement 3—source data 1. [file elife-70464-fig6-figsupp3-data1.zip › Figure 6-figure supplement 3-source data 1/Figure 6-figure supplement 3C-MMP1710-Original.tif]
