# Supplementary material for: aCPSF1 cooperates with terminator U-tract to dictate archaeal transcription termination efficacy
Source: eLife. 2021 Dec 29;10:e70464. doi: 10.7554/eLife.70464 (PMC8716108; doi:10.7554/eLife.70464)
Supplement: Supplementary file 4. [file elife-70464-supp4.docx]

**Supplementary File 4 for**

**aCPSF1 cooperates with terminator U-tract to dictate archaeal transcription termination efficacy**

Jie Li^1, *^^,†^, Lei Yue^1,2,†^, Zhihua Li^1,2, †^, Wenting Zhang^1,2,^, Bing Zhang^2,3^, Fangqing Zhao^2,3^, Xiuzhu Dong^1,2,*^

1, State Key Laboratory of Microbial Resources, Institute of Microbiology, Chinese Academy of Sciences, Beijing 100101, PR China

2, University of Chinese Academy of Sciences, No.19A Yuquan Road, Shijingshan District, Beijing 100049, China

3, Beijing Institutes of Life Science, Chinese Academy of Sciences, Beijing 100101, China

*, Correspondence to: Xiuzhu Dong, No.1 Beichen West Road, Beijing 100101. Tel. 86-10-6480 7413, Email: [dongxz@im.ac.cn；and](mailto:dongxz@im.ac.cn；and) Jie Li, No.1 Beichen West Road, Beijing 100101. Tel. 86-10-6480 7567. Email: [lijie824@im.ac.cn](mailto:lijie824@im.ac.cn).

†, these authors equally contributed.

**This file contains:**

**Supplementary File 4a to 4e**;

**Supplementary Tables**

**Supplementary File 4a. Strains and plasmids used in this study**

| **Strains and** **plasmids** | **Characteristics and descriptions** | | **Reference or sources** | | |
| --- | --- | --- | --- | --- | --- |
| **Strains** |  | |  | | |
| *E. coli* DH5α | F^-^φ80d *lac*ZΔM15 Δ(*lac*ZYA-*arg* F) U169 *end*A1 *rec*A1 *hsd*R17(r_k_^-^,m_k_^+^) *sup*E44λ- *thi* -1 *gyr*A96 *rel*A1 *pho*A | TransGen, Beijing | |  |  |
| *E. coli* BL21(DE3)pLysS | F^-^ *omp*T *hsd*S(r_B_^-^m_B_^-^) *gal dcm*(DE3)pLysS Cam^r^ | | TransGen, Beijing | |  |
| *M. maripaludis* S2 (*Mmp*) | Wild-type *M. maripaludis*, Pur^S^, Neo^S^ | | (Sarmiento et al., 2011) | |  |
| *Mmp-*▽*aCPSF1* | *hpt*::P*mcr*-*tetR*-T*mcr*-P*nif*-*tetO*-His_6_-*MMP0694*, *MMP0694*::*pac*, Pur^R^, strain *tetO*-*aCPSF1* with the indigenous *MMP0694* deletion | | (Yue et al., 2020) | |  |
| *Mmp-*com(*Mmp*-C1) | *hpt*::P*mcr*-*tetR*-T*mcr*-P*nif*-*tetO*-His_6_-*MMP0694*, *MMP0694*::*pac*, pMEV2-*MMP0694*, Pur^R^, Neo^R^, ▽*aCPSF1* with *MMP0694* complement | | (Yue et al., 2020) | |  |
| *Mmp-*com(*Mmp*-C1mu) | *hpt*::P*mcr*-*tetR*-T*mcr*-P*nif*-*tetO*-His_6_-*MMP0694*, *MMP0694*::*pac*, pMEV2-*MMP0694*H243/246A, Pur^R^, Neo^R^, ▽*aCPSF1* with *MMP0694*H243/246A complement | | (Yue et al., 2020) | |  |
| *Mmp-*com(*Loki*-C1) | *hpt*::P*mcr*-*tetR*-T*mcr*-P*nif*-*tetO*-His_6_-*MMP0694*, *MMP0694*::*pac*, pMEV2-*Lokiarch44440*, Pur^R^, Neo^R^, ▽*aCPSF1* with *Lokiarch44440* complement | | (Yue et al., 2020) | |  |
| *Mmp-*com(*Csy*-C1) | *hpt*::P*mcr*-*tetR*-T*mcr*-P*nif*-*tetO*-His_6_-*MMP0694*, *MMP0694*::*pac*, pMEV2-*CENSYa1545*, Pur^R^, Neo^R^, ▽*aCPSF1* with *CENSYa1545* complement | | (Yue et al., 2020) | |  |
| *Mmp-*S2*-*pMEV2 | pMEV2, Neo^R^, S2 with pMEV2 | | (Yue et al., 2020) | |  |
| *Mmp-*com(*Mmp*-C1-ΔKH) | *hpt*::P*mcr*-*tetR*-T*mcr*-P*nif*-*tetO*-His_6_-*MMP0694*, *MMP0694*::*pac*, pMEV2-*MMP0694*-ΔKH, Pur^R^, Neo^R^, ▽*aCPSF1* with *MMP0694*-ΔKH complement | | This study | |  |
| *Mmp-*com(*Mmp*-C1-ΔC13) | *hpt*::P*mcr*-*tetR*-T*mcr*-P*nif*-*tetO*-His_6_-*MMP0694*, *MMP0694*::*pac*, pMEV2-*MMP0694*-ΔC13, Pur^R^, Neo^R^, ▽*aCPSF1* with *MMP0694*-ΔC13 complement | | This study | |  |
| *Mmp-*T0204 | Wild-type *M. maripaludis* with the fragment P*1697*-*luciferase*-T*0204*-*mCherry*-T*0952* inserted into the IGR between *MMP0852* and *MMP0853* | | This study | |  |
| *Mmp-*T0229 | Wild-type *M. maripaludis* with the fragment P*1697*-*luciferase*-T*0229*-*mCherry*-T*0952* inserted into the IGR between *MMP0852* and *MMP0853* | | This study | |  |
| *Mmp-*T0911 | Wild-type *M. maripaludis* carrying the fragment P*1697*-*luciferase*-T*0911*-*mCherry*-T*0952* inserted into the IGR between *MMP0852* and *MMP0853* | | This study | |  |
| *Mmp-*T1149 | Wild-type *M. maripaludis* with the fragment P*1697*-*luciferase*-T*1149*-*mCherry*-T*0952* inserted into the IGR between *MMP0852* and *MMP0853* | | This study | |  |
| *Mmp-*T1710 | Wild-type *M. maripaludis* with the fragment P*1697*-*luciferase*-T*1710*-*mCherry*-T*0952* inserted into the IGR between *MMP0852* and *MMP0853* | | This study | |  |
| *Mmp-*▽*aCPSF1-*T0204 | *Mmp-*▽*aCPSF1* with the fragment P*1697*-*luciferase*-T*0204*-*mCherry*-T*0952* inserted into the IGR between *MMP0852* and *MMP0853* | | This study | |  |
| *Mmp-*▽*aCPSF1-*T0229 | *Mmp-*▽*aCPSF1* with the fragment P*1697*-*luciferase*-T*0229*-*mCherry*-T*0952* inserted into the IGR between *MMP0852* and *MMP0853* | | This study | |  |
| *Mmp-*▽*aCPSF1-*T0911 | *Mmp-*▽*aCPSF1* with the fragment P*1697*-*luciferase*-T*0911*-*mCherry*-T*0952* inserted into the IGR between *MMP0852* and *MMP0853* | | This study | |  |
| *Mmp-*▽*aCPSF1-*T1149 | *Mmp-*▽*aCPSF1* with the fragment P*1697*-*luciferase*-T*1149*-*mCherry*-T*0952* inserted into the IGR between *MMP0852* and *MMP0853* | | This study | |  |
| *Mmp-*▽*aCPSF1-*T1710 | *Mmp-*▽*aCPSF1* with the fragment P*1697*-*luciferase*-T*1710*-*mCherry*-T*0952* inserted into the IGR between *MMP0852* and *MMP0853* | | This study | |  |
| *Mmp-*com(*Mmp*-C1)*-*T0204 | *Mmp-*com(*Mmp*-C1) with the fragment P*1697*-*luciferase*-T*0204*-*mCherry*-T*0952* inserted into the IGR between *MMP0852* and *MMP0853* | | This study | |  |
| *Mmp-*com(*Mmp*-C1)*-*T0229 | *Mmp-*com(*Mmp*-C1) with the fragment P*1697*-*luciferase*-T*0229*-*mCherry*-T*0952* inserted into the IGR between *MMP0852* and *MMP0853* | | This study | |  |
| *Mmp-*com(*Mmp*-C1)*-*T0911 | *Mmp-*com(*Mmp*-C1) with the fragment P*1697*-*luciferase*-T*0911*-*mCherry*-T*0952* inserted into the IGR between *MMP0852* and *MMP0853* | | This study | |  |
| *Mmp-*com(*Mmp*-C1)*-*T1149 | *Mmp-*com(*Mmp*-C1) with the fragment P*1697*-*luciferase*-T*1149*-*mCherry*-T*0952* inserted into the IGR between *MMP0852* and *MMP0853* | | This study | |  |
| *Mmp-*com(*Mmp*-C1)*-*T1710 | *Mmp-*com(*Mmp*-C1) with the fragment P*1697*-*luciferase*-T*1710*-*mCherry*-T*0952* inserted into the IGR between *MMP0852* and *MMP0853* | | This study | |  |
| *Mmp-*com(*Mmp*-C1mu)*-*T0204 | *Mmp-*com(*Mmp*-C1mu) with the fragment P*1697*-*luciferase*-T*0204*-*mCherry*-T*0952* inserted into the IGR between *MMP0852* and *MMP0853* | | This study | |  |
| *Mmp-*com(*Mmp*-C1mu)*-*T0229 | *Mmp-*com(*Mmp*-C1mu) with the fragment P*1697*-*luciferase*-T*0229*-*mCherry*-T*0952* inserted into the IGR between *MMP0852* and *MMP0853* | | This study | |  |
| *Mmp-*com(*Mmp*-C1mu)*-*T0911 | *Mmp-*com(*Mmp*-C1mu) with the fragment P*1697*-*luciferase*-T*0911*-*mCherry*-T*0952* inserted into the IGR between *MMP0852* and *MMP0853* | | This study | |  |
| *Mmp-*com(*Mmp*-C1mu)*-*T1149 | *Mmp-*com(*Mmp*-C1mu) with the fragment P*1697*-*luciferase*-T*1149*-*mCherry*-T*0952* inserted into the IGR between *MMP0852* and *MMP0853* | | This study | |  |
| *Mmp-*com(*Mmp*-C1mu)*-*T1710 | *Mmp-*com(*Mmp*-C1) with the fragment P*1697*-*luciferase*-T*1710*-*mCherry*-T*0952* inserted into the IGR between *MMP0852* and *MMP0853* | | This study | |  |
| *Mmp-*com(*Loki*-C1)*-*T0204 | *Mmp-*com(*Loki*-C1) with the fragment P*1697*-*luciferase*-T*0204*-*mCherry*-T*0952* inserted into the IGR between *MMP0852* and *MMP0853* | | This study | |  |
| *Mmp-*com(*Loki*-C1)*-*T0229 | *Mmp-*com(*Loki*-C1) with the fragment P*1697*-*luciferase*-T*0229*-*mCherry*-T*0952* inserted into the IGR between *MMP0852* and *MMP0853* | | This study | |  |
| *Mmp-*com(*Loki*-C1)*-*T0911 | *Mmp-*com(*Loki*-C1) with the fragment P*1697*-*luciferase*-T*0911*-*mCherry*-T*0952* inserted into the IGR between *MMP0852* and *MMP0853* | | This study | |  |
| *Mmp-*com(*Loki*-C1)*-*T1149 | *Mmp-*com(*Loki*-C1) with the fragment P*1697*-*luciferase*-T*1149*-*mCherry*-T*0952* inserted into the IGR between *MMP0852* and *MMP0853* | | This study | |  |
| *Mmp-*com(*Loki*-C1)*-*T1710 | *Mmp-*com(*Loki*-C1) with the fragment P*1697*-*luciferase*-T*1710*-*mCherry*-T*0952* inserted into the IGR between *MMP0852* and *MMP0853* | | This study | |  |
| *Mmp-*com(*Csy*-C1)*-*T0204 | *Mmp-*com(*Loki*-C1) with the fragment P*1697*-*luciferase*-T*0204*-*mCherry*-T*0952* inserted into the IGR between *MMP0852* and *MMP0853* | | This study | |  |
| *Mmp-*com(*Csy*-C1)*-*T0229 | *Mmp-*com(*Loki*-C1) with the fragment P*1697*-*luciferase*-T*0229*-*mCherry*-T*0952* inserted into the IGR between *MMP0852* and *MMP0853* | | This study | |  |
| *Mmp-*com(*Csy*-C1)*-*T0911 | *Mmp-*com(*Loki*-C1) with the fragment P*1697*-*luciferase*-T*0911*-*mCherry*-T*0952* inserted into the IGR between *MMP0852* and *MMP0853* | | This study | |  |
| *Mmp-*com(*Csy*-C1)*-*T1149 | *Mmp-*com(*Loki*-C1) with the fragment P*1697*-*luciferase*-T*1149*-*mCherry*-T*0952* inserted into the IGR between *MMP0852* and *MMP0853* | | This study | |  |
| *Mmp-*com(*Csy*-C1)*-*T1710 | *Mmp-*com(*Loki*-C1) with the fragment P*1697*-*luciferase*-T*1710*-*mCherry*-T*0952* inserted into the IGR between *MMP0852* and *MMP0853* | | This study | |  |
| *Mmp-*com(*Mmp*-C1-ΔKH)*-*T0204 | *Mmp-*com(*Loki*-C1) with the fragment P*1697*-*luciferase*-T*0204*-*mCherry*-T*0952* inserted into the IGR between *MMP0852* and *MMP0853* | | This study | |  |
| *Mmp-*com(*Mmp*-C1-ΔKH)*-*T0229 | *Mmp-*com(*Loki*-C1) with the fragment P*1697*-*luciferase*-T*0229*-*mCherry*-T*0952* inserted into the IGR between *MMP0852* and *MMP0853* | | This study | |  |
| *Mmp-*com(*Mmp*-C1-ΔKH)*-*T0911 | *Mmp-*com(*Loki*-C1) with the fragment P*1697*-*luciferase*-T*0911*-*mCherry*-T*0952* inserted into the IGR between *MMP0852* and *MMP0853* | | This study | |  |
| *Mmp-*com(*Mmp*-C1-ΔKH)*-*T1149 | *Mmp-*com(*Loki*-C1) with the fragment P*1697*-*luciferase*-T*1149*-*mCherry*-T*0952* inserted into the IGR between *MMP0852* and *MMP0853* | | This study | |  |
| *Mmp-*com(*Mmp*-C1-ΔKH)*-*T1710 | *Mmp-*com(*Loki*-C1) with the fragment P*1697*-*luciferase*-T*1710*-*mCherry*-T*0952* inserted into the IGR between *MMP0852* and *MMP0853* | | This study | |  |
| *Mmp-*T0204-24 | Wild-type *M. maripaludis* with the fragment P*1697*-*luciferase*-T*0204*-24-*mCherry*-T*0952* inserted into the IGR between *MMP0852* and *MMP0853* | | This study | |  |
| *Mmp-*T0204-24-M1 | Wild-type *M. maripaludis* with the fragment P*1697*-*luciferase*-T*0204*-24-M1-*mCherry*-T*0952* inserted into the IGR between *MMP0852* and *MMP0853* | | This study | |  |
| *Mmp-*T0204-24-M2 | Wild-type *M. maripaludis* with the fragment P*1697*-*luciferase*-T*0204*-24-M2-*mCherry*-T*0952* inserted into the IGR between *MMP0852* and *MMP0853* | | This study | |  |
| *Mmp-*T0204-24-M3 | Wild-type *M. maripaludis* with the fragment P*1697*-*luciferase*-T*0204*-24-M3-*mCherry*-T*0952* inserted into the IGR between *MMP0852* and *MMP0853* | | This study | |  |
| *Mmp-*▽*aCPSF1-*T0204-24 | *Mmp-*▽*aCPSF1* with the fragment P*1697*-*luciferase*-T*0204*-24-*mCherry*-T*0952* inserted into the IGR between *MMP0852* and *MMP0853* | | This study | |  |
| *Mmp-*▽*aCPSF1-*T0204-24-M1 | *Mmp-*▽*aCPSF1* with the fragment P*1697*-*luciferase*-T*0204*-24-M1-*mCherry*-T*0952* inserted into the IGR between *MMP0852* and *MMP0853* | | This study | |  |
| *Mmp-*▽*aCPSF1-*T0204-24-M2 | *Mmp-*▽*aCPSF1* with the fragment P*1697*-*luciferase*-T*0204*-24-M2-*mCherry*-T*0952* inserted into the IGR between *MMP0852* and *MMP0853* | | This study | |  |
| *Mmp-*▽*aCPSF1-*T0204-24-M3 | *Mmp-*▽*aCPSF1* with the fragment P*1697*-*luciferase*-T*0204*-24-M3-*mCherry*-T*0952* inserted into the IGR between *MMP0852* and *MMP0853* | | This study | |  |
| **Plasmids** |  | |  | | |
| pMD19-T | Amp^R^ | | Takara, Japan | | |
| pMD19-T-T0204 | pMD19-T with *MMP0852*up- P*1697*-*luciferase*-T*0204*-*mCherry*-T*0952-MMP0853*down inserted into T-overhang end, Amp^R^ | | This study | | |
| pMD19-T-T0229 | pMD19-T with *MMP0852*up- P*1697*-*luciferase*-T*0229*-*mCherry*-T*0952-MMP0853*down inserted into T-overhang end, Amp^R^ | | This study | | |
| pMD19-T-T0911 | pMD19-T with *MMP0852*up- P*1697*-*luciferase*-T*0911*-*mCherry*-T*0952-MMP0853*down inserted into T-overhang end, Amp^R^ | | This study | | |
| pMD19-T-T1149 | pMD19-T with *MMP0852*up- P*1697*-*luciferase*-T*1149*-*mCherry*-T*0952-MMP0853*down inserted into T-overhang end, Amp^R^ | | This study | | |
| pMD19-T-T1710 | pMD19-T with *MMP0852*up- P*1697*-*luciferase*-T*1710*-*mCherry*-T*0952-MMP0853*down inserted into T-overhang end, Amp^R^ | | This study | | |
| pMD19-T-T0204-24 | pMD19-T with *MMP0852*up- P*1697*-*luciferase*-T*0204*-24-*mCherry*-T*0952-MMP0853*down inserted into T-overhang end, Amp^R^ | | This study | | |
| pMD19-T-T0204-24-M1 | pMD19-T with *MMP0852*up- P*1697*-*luciferase*-T*0204*-24-M1-*mCherry*-T*0952-MMP0853*down inserted into T-overhang end, Amp^R^ | | This study | | |
| pMD19-T-T0204-24-M2 | pMD19-T with *MMP0852*up- P*1697*-*luciferase*-T*0204*-24-M2-*mCherry*-T*0952-MMP0853*down inserted into T-overhang end, Amp^R^ | | This study | | |
| pMD19-T-T0204-24-M3 | pMD19-T with *MMP0852*up- P*1697*-*luciferase*-T*0204*-24-M3-*mCherry*-T*0952-MMP0853*down inserted into T-overhang end, Amp^R^ | | This study | | |
| pMEV2 | Amp^R^, Neo^R^ | | (Sarmiento et al., 2011) | | |
| pMEV2-*MMP0694-*ΔKH | pMEV2 with *MMP0695* promoter, 5′ UTR and ORF of *MMP0694-*ΔKH inserted between *Xho* Ⅰ and *Bgl* Ⅱ, Amp^R^, Neo^R^ | | This study | | |
| pET28a | Kan^R^ | |  | | |
| pET28a *MMP0694-*KH | pET28a with N-terminal KH domain (1-147aa) of *Mmp*-aCPSF1 inserted into the expression region, Kan^R^ | | This study | | |
| pSB1s-His_6_-SUMO-*aCPSF1* | pSB1s with His_6_-SUMO and *MMP0694* genes inserted into the expression region, Str^R^ | |  | | |

**Supplementary File 4b. Primers used in this study**

| Primer | Sequence (5′-3′) | Purpose |
| --- | --- | --- |
| pMEV2-aCPSF1-ΔKH-F | gactattatgTGGGTTAGAACTTCGTTTTTAG | Construction of complementary strain |
| pMEV2-aCPSF1-ΔKH-R | ttctaacccaCATAATAGTCCCTCCTAATATTAATC | Construction of complementary strain |
| pMEV2-aCPSF1-ΔC113-F | ttaaaaaagaaTAAAGATCTCATGATATCTAGATC | Construction of complementary strain |
| pMEV2-aCPSF1-ΔC13-R | gagatctttaTTCTTTTTTAAATAACTTGTATGCC | Construction of complementary strain |
| pET28a-F | TGAGATCCGGCTGCTAACAAAG | Protein expression of *Mmp*-aCPSF1-KH |
| pET28a-R | CATGCTAGCCATATGGCTG | Protein expression of *Mmp*-aCPSF1-KH |
| aC1-KH-(28a)-F | gcagccatatggctagcatgAGCGCGGAAGATATCCTG | Protein expression of *Mmp*-aCPSF1-KH |
| aC1-KH-(28a)-R | ttgttagcagccggatctcaGCGGATCGCTTTAATGGTATC | Protein expression of *Mmp*-aCPSF1-KH |
| MMP0852up-F | CACAAGCGCAGAATTATAAGCA | Construction of reporter system |
| MMP0853down-R | CGATGTTGTCTCTTGGAACAAAG | Construction of reporter system |
| 19T-0852/3-F | TTATTTTTTTGATAGGGGCG | Construction of reporter system |
| 19T-0852/3-R | AATTAAAAAAATCAGGGAAATTTAC | Construction of reporter system |
| Ter-reporter-(0852/3)-F | tttccctgatttttttaattATAATCTATAGTAATTACAAAAATATATAAAAAAAGG | Construction of reporter system |
| Ter-reporter-(0852/3)-R | cgcccctatcaaaaaaataaCGATAATAGTCCCATAAAATTTAAAAAAAG | Construction of reporter system |
| T0229-Gibson-F | ttaaggatccATATAACATAAAAATAAATTAATATTTTTTTGAATGGAGCTCACTAGAGTGCAG | Construction of reporter system |
| T0229-Gibson-R | tagtgagctcCATTCAAAAAAATATTAATTTATTTTTATGTTATATGGATCCTTAAACTGCAATTTTTC | Construction of reporter system |
| T0911-Gibson-F | ttaaggatccGAAATTATAATACATTCATAATTTATTTTTTAATTAGAGCTCACTAGAGTGCAG | Construction of reporter system |
| T0911-Gibson-R | tagtgagctcTAATTAAAAAATAAATTATGAATGTATTATAATTTCGGATCCTTAAACTGCAATTTTTC | Construction of reporter system |
| T1149-Gibson-F | ttaaggatccAAACCTTATTTTTTTACTTTTTTTAAATTTTAAAGTGAGCTCACTAGAGTGCAG | Construction of reporter system |
| T1149-Gibson-R | tagtgagctcACTTTAAAATTTAAAAAAAGTAAAAAAATAAGGTTTGGATCCTTAAACTGCAATTTTTC | Construction of reporter system |
| T1710-Gibson-F | ttaaggatccAAGAAAAGCTAGTGAACTGAAATGGGGTCAAAGACAGAGCTCACTAGAGTGCAG | Construction of reporter system |
| T1710-Gibson-R | tagtgagctcTGTCTTTGACCCCATTTCAGTTCACTAGCTTTTCTTGGATCCTTAAACTGCAATTTTTC | Construction of reporter system |
| T0204-24-Gibson-F | ttaaggatccCTACTTTTTTTTATTTTTAAACGAGAGCTCACTAGAGTGCAG | Construction of reporter system |
| T0204-24-Gibson-R | tagtgagctcTCGTTTAAAAATAAAAAAAAGTAGGGATCCTTAAACTGCAATTTTTC | Construction of reporter system |
| T0204-24M1-Gibson-F | ttaaggatccCTACccccccccATTTTTAAACGAGAGCTCACTAGAGTGCAG | Construction of reporter system |
| T0204-24M1-Gibson-R | tagtgagctcTCGTTTAAAAATggggggggGTAGGGATCCTTAAACTGCAATTTTTC | Construction of reporter system |
| T0204-24M2-Gibson-F | ttaaggatccCTACTTTTTTTTAcccccAAACGAGAGCTCACTAGAGTGCAG | Construction of reporter system |
| T0204-24M2-Gibson-R | tagtgagctcTCGTTTgggggTAAAAAAAAGTAGGGATCCTTAAACTGCAATTTTTC | Construction of reporter system |
| T0204-24M3-Gibson-F | ttaaggatccCTACccatccatAcccccAAACGAGAGCTCACTAGAGTGCAG | Construction of reporter system |
| T0204-24M3-Gibson-R | tagtgagctcTCGTTTGGGGGTATGGATGGGTAGGGATCCTTAAACTGCAATTTTTC | Construction of reporter system |
| 0400-3′R-N1-F | AAAGCTGTCGAAGAAGT | 3′RACE |
| 0400-3′R-N2-F | CCAGTAATATGCTTAAACAT | 3′RACE |
| 0457-3′R-N1-F | ATTAGTGGATCACATTTCAAGA | 3′RACE |
| 0457-3′R-N2-F | AAAGGAAGAGACATCGAC | 3′RACE |
| 1579-3′R-N1-F | AGAAGATCTCTTCAACTTAATGA | 3′RACE |
| 1579-3′R-N2-F | AACTTGAGAAACCACTTGG | 3′RACE |
| 0229-3′R-N1-F | TATGAAGGAATGACGATG | 3′RACE |
| 0229-3′R-N2-F | TAACTCACGAAATGGGT | 3′RACE |
| 1406-3′R-N1-F | AAGAAGGTTTGGTAAGCT | 3′RACE |
| 1406-3′R-N2-F | CTTTGATGCGGACTTGA | 3′RACE |
| 1710-3′R-N1-F | GTAAGAGTCATCACTAAGTTCC | 3′RACE |
| 1710-3′R-N2-F | ACAGTATGTTTTACACAAAAATGA | 3′RACE |
| 1147-3′R-N1-F | GGAAGAAGAGCATTCCACGTAA | 3′RACE |
| 1147-3′R-N2-F | TATGTGCAGCATGCGGATTT | 3′RACE |
| 1242-3′R-N1-F | GAACTGACGCAATCTGCAAA | 3′RACE |
| 1242-3′R-N2-F | CGATGAAAAACATAACTTTAGAAAAAATAG | 3′RACE |
| 1149-3′R-N1-F | CATGCTTGAAGCTATTGATGAAGG | 3′RACE |
| 1149-3′R-N2-F | CCATCATGTTCTGCATGTATGG | 3′RACE |
| 0901-3′R-N1-F | CGTTCCAATTTTAGTTGCACTTAC | 3′RACE |
| 0901-3′R-N2-F | GAAGAAGATCTTGGAGAATATGAAGT | 3′RACE |
| 1224-3′R-N1-F | GTTACTGATCAGATCCAGC | 3′RACE |
| 1224-3′R-N2-F | TAATTTTGAATCCAGATTGATTTTC | 3′RACE |
| luciferase- mCherry-SC-F | TCATGCAAGAGATCCTATTTTTGG | qRT-PCR |
| luciferase- mCherry-SC-R | GCTTCCATTAATCTTCTTAATGGTGT | qRT-PCR |
| mCherry-qPCR-F | tggaaggttcagttaatggtcac | qRT-PCR |
| mCherry-qPCR-R | gctgggtgtttaacgtatgc | qRT-PCR |
| luciferase-qPCR-F | GTAGCACCAGCAGAATTAGAATCAA | qRT-PCR |
| luciferase-qPCR-R | GCTGTTGTAACTTGTGATGCAAC | qRT-PCR |

**Supplementary File 4c. The statistical significance of TTEs in the four terminator groups carrying different U4-tract numbers shown in Figure 1D.**

| **Group** | **>2** | **2** | **1** | **0** |
| --- | --- | --- | --- | --- |
| **>2** | NA | 0.033893 | 4.57E-09 | 2.05E-19 |
| **2** | 0.033892841 | NA | 6.44E-06 | 1.74E-17 |
| **1** | 4.57E-09 | 6.44E-06 | NA | 9.95E-06 |
| **0** | 2.05E-19 | 1.74E-17 | 9.95E-06 | NA |

The statistical significance analysis was performed by Wilcoxon rank sum test to calculate P-values for TTEs of terminators carrying more than 2 (>2), 2, 1 and 0 U4-tracts, respectively. P-value smaller than 0.05 was thought to be statistical significance. NA, not applicable.

**Supplementary File 4d**. **The statistical significance analysis on TQRRs of the four terminator groups carrying different U4-tract numbers shown in Figure 2D.**

| **Group** | **>2** | **2** | **1** | **0** |
| --- | --- | --- | --- | --- |
| **>2** | NA | 0.190083 | 3.08E-06 | 2.54E-23 |
| **2** | 0.190083 | NA | 1.14E-05 | 1.05E-27 |
| **1** | 3.08E-06 | 1.14E-05 | NA | 6.63E-10 |
| **0** | 2.54E-23 | 1.05E-27 | 6.63E-10 | NA |

The statistical significance analysis was performed by Wilcoxon rank sum test to calculate P-values for TQRRs of terminators carrying more than 2 (>2), 2, 1 and 0 U4-tracts, respectively. P-value smaller than 0.05 was thought to be statistical significance. NA, not applicable.

**Supplementary File 4e.** **The statistical significance analysis on the qRT-PCR data of the tested terminators in the terminator reporter assay shown in Figure 5B.**

|  | WT vs ▽*aCPSF1* | WT vs Com(wt) | WT vs Com(Mu) | WT vs Com(ΔKH) | WT vs Com(*Csy*-aCPSF1) | WT vs Com (*Loki*-aCPSF1) |
| --- | --- | --- | --- | --- | --- | --- |
| **T1149** | 0.0008 | 0.0022 | 0.0002 | 0.0028 | 0.0711 | 0.1592 |
| **T0204** | 0.0016 | 0.0704 | 0.0070 | 0.0048 | 0.2572 | 0.0194 |
| **T0911** | 0.0021 | 0.7313 | 0.0032 | 0.0187 | 0.0113 | 0.0507 |
| **T0229** | 0.0001 | 0.4670 | 0.0001 | 0.0128 | 0.7948 | 0.0610 |
| **T1710** | 0.7944 | 0.8791 | 0.4807 | 0.3544 | 0.4639 | 0.0368 |

The statistical significance analysis was implemented by T-test to calculate P-values the qRT-PCR data in different genetic strains Vs. WT shown in Figure 5B, respectively. The strains were named in the same nomenclatures shown in Figure 5B. P-value smaller than 0.05 was thought to be statistical significance and p-value smaller than 0.01 was thought to be extremely statistical significance.
